# Small extracellular vesicles of hypoxic endothelial cells regulate the therapeutic potential of adipose-derived mesenchymal stem cells via miR-486-5p/PTEN in a limb ischemia model

**DOI:** 10.1186/s12951-022-01632-1

**Published:** 2022-09-24

**Authors:** Zekun Shen, Weiyi Wang, Jinxing Chen, Bingyi Chen, Yanan Tang, Jiaxuan Hou, Jiayan Li, Shuang Liu, Yifan Mei, Liwei Zhang, Shaoying Lu

**Affiliations:** Department of Vascular Surgery, The First Affiliated Hospital of Xi’an Jiaotong University, Xi’an, 710061 China

## Abstract

**Background:**

Patients with critical limb ischemia (CLI) are at great risk of major amputation and cardiovascular events. Adipose-derived mesenchymal stem cell (ADSC) therapy is a promising therapeutic strategy for CLI, but the poor engraftment and insufficient angiogenic ability of ADSCs limit their regenerative potential. Herein, we explored the potential of human umbilical vein endothelial cells (HUVECs)-derived small extracellular vesicles (sEVs) for enhancing the therapeutic efficacy of ADSCs in CLI.

**Results:**

sEVs derived from hypoxic HUVECs enhanced the resistance of ADSCs to reactive oxygen species (ROS) and further improved the proangiogenic ability of ADSCs in vitro. We found that the hypoxic environment altered the composition of sEVs from HUVECs and that hypoxia increased the level of miR-486-5p in sEVs. Compared to normoxic sEVs (nsEVs), hypoxic sEVs (hsEVs) of HUVECs significantly downregulated the phosphatase and tensin homolog (PTEN) via direct targeting of miR-486-5p, therefore activating the AKT/MTOR/HIF-1α pathway and influencing the survival and pro-angiogenesis ability of ADSCs. In a hindlimb ischemia model, we discovered that hsEVs-primed ADSCs exhibited superior cell engraftment, and resulted in better angiogenesis and tissue repair.

**Conclusion:**

hsEVs could be used as a therapeutic booster to improve the curative potential of ADSCs in a limb ischemia model. This finding offers new insight for CLI treatment.

**Graphical Abstract:**

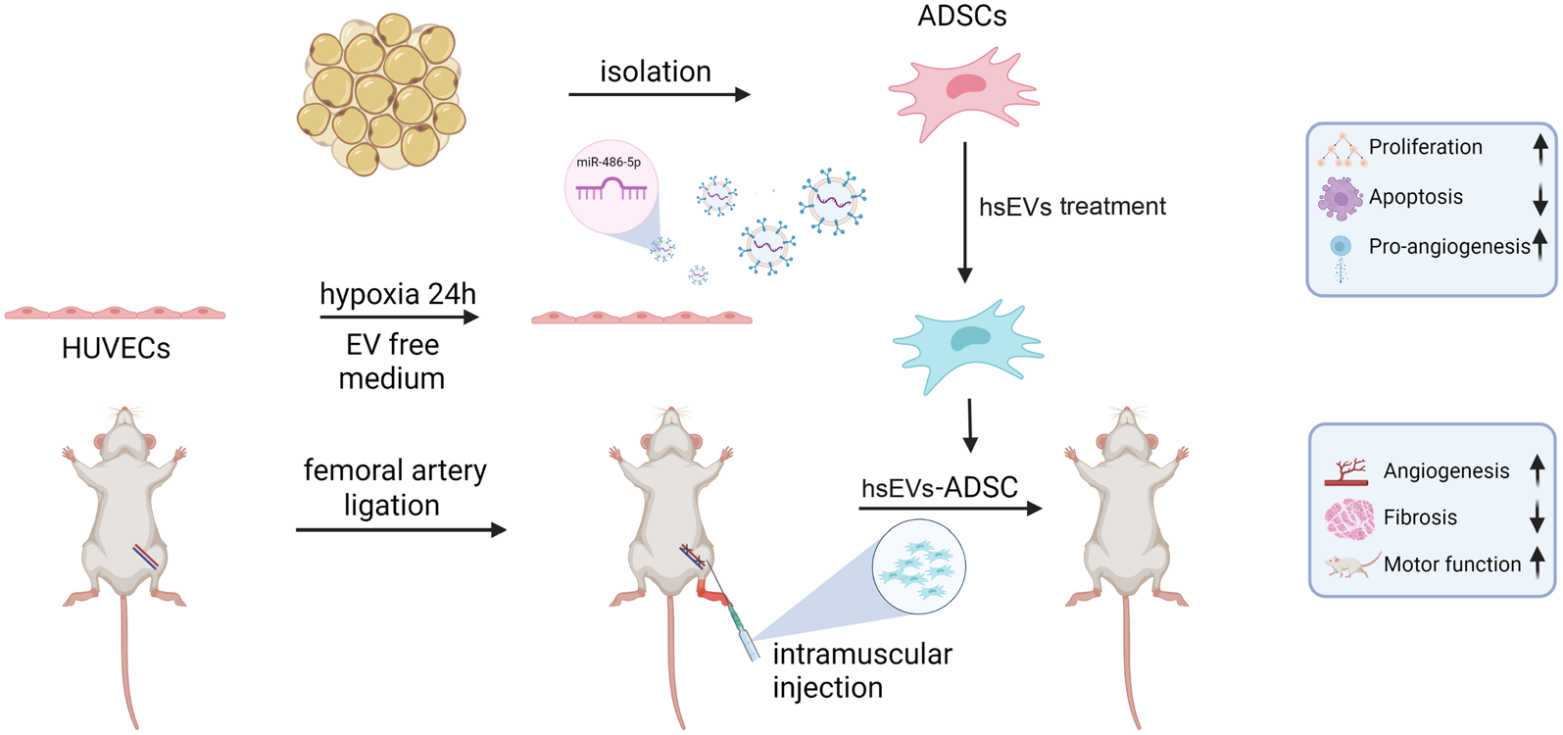

**Supplementary Information:**

The online version contains supplementary material available at 10.1186/s12951-022-01632-1.

## Introduction

Critical limb ischemia (CLI) is the end-stage of peripheral arterial disease and is characterized by chronic ischemic rest pain and tissue loss [1]. Persistent malperfusion of the lower extremities results in inevitable amputation and even death. With increasing risk factors, including old age, diabetes, and smoking, CLI will cause a more significant societal and financial burden [2]. Although surgical and endovascular revascularization improve symptoms and reduce major amputation rates [3], up to 20% of CLI patients are unsuitable for revascularization. The treatment of “no-option” patients is still challenging. Therapeutic angiogenesis is seen as an alternative strategy for these patients because of its potential to increase tissue perfusion [4, 5].

In recent years, cell-based therapies, including endothelial progenitor cells, mesenchymal stem cells (MSCs) and induced pluripotent stem cells (iPSCs), have been widely used to promote tissue repair, partly due to their ability to induce angiogenesis [6]. In 2002, Tsutomu Imaizumi successfully achieved therapeutic angiogenesis in patients with limb ischemia through autologous bone marrow-derived mononuclear cell transplantation [7]. Intramuscular injection of bone marrow-derived mononuclear cells improved the ankle brachial index (ABI), transcutaneous oxygen pressure, pain-free walk time and rest pain. Since then, several cell-based therapy studies have been carried out in which autologous cell transplantation successfully promotes tissue repair [8–10]. MSCs are the most commonly used cells in treating CLI, and MSCs exert proangiogenic and tissue regeneration through paracrine and immunomodulatory effects in a CLI model [11]. However, the harsh environment of injection sites, such as ischemia and inflammation, leads to early cell death and limits the efficiency of cell-based therapy [12]. Therefore, improving the survival of MSCs after transplantation and increasing their potential in treating CLI has attracted intense interest [13, 14]. Among all MSC populations, adipose-derived mesenchymal stem cells (ADSCs) are one of the most promising cell types because ADSCs can be extracted from less invasive approaches and obtained in large amounts.

Preconditioning and genetic modification are commonly used MSC enhancement strategies [15]. MSCs can “remember” the environment they are exposed to, and preconditioning by different stimuli in vitro is able to prepare MSCs for the in vivo environment. Hypoxic preconditioning has also been proven to upregulate superoxide dismutase 2 (SOD2), making ADSCs more resistant to ROS generated from ischemia and improving retention in vivo [16]. Inflammatory cytokines, such as interferon gamma (IFN-γ) [17, 18] and tumor necrosis factor alpha (TNF-α) [19, 20], have been considered able to mimic the inflammatory environment after transplantation and to boost the immunomodulatory effects of ADSCs. Genetic manipulation of apoptosis [21], proliferation and migration-related genes [22] has also been effective in promoting the therapeutic effects of ADSCs. Given the lentivirus and adenovirus used in genetic modification, preconditioning is a relatively safer choice.

A recent study discovered that small extracellular vesicles (sEVs) of human umbilical vein endothelial cells (HUVECs) protected human heart-on-chip from ischemia/reperfusion damage [23]. The sEVs of HUVECs also have protective effects on cardiomyocytes, neural cells and neural stem cells [24–28]. In addition, researchers have discovered that sEVs from HUVECs enhance the angiogenic activity of endothelial progenitor cells [29]. The mixture of MSCs and HUVECs presents a stronger regenerative ability in a limb ischemia model, potentially via cell–cell communication [30]. However, the effects of sEVs from HUVECs on ADSCs remain unclear.

We used normoxic sEVs (nsEVs) and hypoxic sEVs (hsEVs) to pretreat ADSCs and tested the proliferation and apoptosis under reactive oxygen species (ROS) stress and the proangiogenic ability of ADSCs in vitro. Next, firefly luciferase and enhanced green fluorescent protein (Fluc-eGFP) double-report ADSCs were used to study the survival of ADSCs in a hindlimb ischemia model. Finally, sEVs-treated ADSCs were transplanted into hindlimb ischemic mice, and tissue regeneration was evaluated by laser Doppler, histopathology and motor and tissue damage scoring. Mechanistically, we found that hsEVs transferred miR-486-5p and activated the AKT signaling pathway by targeting PTEN in ADSCs. Our data suggest that hsEVs priming is a novel approach for improving the therapeutic effects of ADSCs in CLI.

## Materials and methods

### Isolation and characteristics of ADSCs

Human subcutaneous adipose tissue was obtained through liposuction with the patients’ written informed consent in the first affiliated hospital of Xi’an Jiaotong University. The human ethics committee approved the use of human-derived cells. Fresh isolated adipose tissue was cut into pieces, washed with phosphate buffered saline (PBS) and centrifuged at 1000 rpm for 5 min at room temperature. The lower part was discarded, and then the residual adipose tissue was digested with collagenase I (#SCR103, Sigma–Aldrich, St Louis, MI, USA) for 1 h at 37 ℃. The equivalent volume of complete Dulbecco’s modified Eagle’s medium F-12 (DMEM/F-12) (Gibco, Amarillo, TX, USA) was added into the tube to suspend digestion. Cells were collected after washing with PBS and then seeded in a 60 mm cell culture dish (Corning, NY, USA). Once reached confluence, adherent cells (passage 0) were digested with 0.125% trypsin-ethylenediaminetetraacetic acid (EDTA) and passaged at a 1:3 split ratio in 60 mm cell culture dishes. Cells of passage 5–10 were used in this study. ADSCs were stained with fluorescence-labelled monoclonal antibodies against CD29, CD44, CD90, CD105, CD45 and CD34 (eBioscience, San Diego, CA, USA) for flow cytometry (FCM) to analyze their surface phenotypes.

### Cell culture and reagents

HUVEC was purchased from the American type culture collection (ATCC, Manassas, VR, USA). The medium for HUVECs was endothelial cell medium (ECM; ScienCell, Carlsbad, CA, USA) containing 5% fetal bovine serum (FBS; ScienCell, Carlsbad, CA, USA) and supplemented with endothelial cell growth supplement (ScienCell, Carlsbad, CA, USA). ADSCs were maintained in DMEM/F12 with the following components: 10% FBS and 100 unit/mL penicillin–streptomycin. All cells were negative in mycoplasma tests and maintained in a humidified incubator with 5% CO_2_ at 37 ℃. MK2206 (HY-108232), rapamycin (HY-10219) and PX-478(HY-10231) were purchased from MCE (MCE, Monmouth Junction, New Jersey, USA).

### Isolation and characterization of sEVs

sEVs were isolated from the supernatants of HUVECs under normoxic (21% O_2_, 5% CO_2_) or hypoxic (1% O_2_, 5% CO_2_) condition through differential centrifugation as previously described [31]. In brief, FBS was ultracentrifuged at 100,000*g* for 18 h to deplete extracellular vesicles and acquire EV-depleted FBS. HUVECs were changed to EV-depleted ECM after reaching 70% of confluence, and then HUVECs were moved to hypoxia gas chamber for 24 h (1% O_2_, 5% CO_2_; Baker, Bridgend Industrial Estate, Bridgend, South Wales). Afterwards, the culture medium was collected and centrifugated at 300*g* for 10 min to remove any cell contaminations, followed by centrifugation of 2000*g* for 10 min to remove dead cells; then, the supernatant was centrifugated at 10,000*g* for 30 min to remove cell debris. Eventually, sEVs were collected by ultracentrifugation at 100,000*g* for 70 min at 4℃ for two times. The pellet was suspended by PBS and stored at – 80 ℃. A BCA kit (Thermo Fisher Scientific, Waltham, MA, USA) was used to quantify the protein concentration of sEVs in PBS Identification of sEVs followed the Minimal Information for Studies of Extracellular Vesicles 2018 (MISEV2018) guideline [32]. The particle size of sEVs was determined by a flow nanoanalyzer (nanoFCM, Xiamen, Fujian, China). The morphology of the sEVs was identified by transmission electron microscopy (TEM; Hitachi, Chiyoda-ku, Tokyo, Japan).

### In vitro internalization of dil-labeled sEVs

To visualize the internalization of sEVs by ADSCs, a red lipophilic fluorescent dye 1,1′-dioctadecyl-3,3,3′,3′-tetramethylindocarbocyanine perchlorate (Dil; Beyotime, Shanghai, China) was used to label sEVs. Isolated sEVs were incubated with 10 µM Dil for 10 min at room temperature. Free dye was discarded by ultracentrifugation at 100,000*g* for 70 min at 4 ℃, and the labelled sEVs were suspended by complete DMEM/F12 medium to a final concentration of 20 µg/mL. After incubating with the medium containing Dil-sEVs for 12 h, ADSCs were washed by PBS three times. And then, ADSCs were fixed with 4% paraformaldehyde for 15 min at room temperature and washed by PBS three times. Anti-Tracker Green-488 (Beyotime, Shanghai, China) and 4′-6-diamidino-2-phenylindole (DAPI, Vector Laboratories, Burlingame, CA, USA) were used to stain the cytoskeleton and nuclei of ADSCs. Stained ADSCs were observed under a confocal microscope (Lecia, Wetzlar, Germany).

### Cell proliferation analysis and apoptosis assays

A Cell Counting Kit-8 (CCK-8; TargetMol, Boston, MA, USA) was used following the manufacturer’s protocol. ADSCs (3 × 10^3^ cells/well) were seeded in 96-well plates with FPS-free medium in the presentation of sEVs at different concentrations for 24 h and 48 h. Then the culture medium in each well was replaced by a basic culture medium containing 10% CCK-8 solution. Plates were subsequently incubated at 37 ℃ for 2 h. Proliferation rates were calculated by the absorbance values at 450 nm using a microplate reader (Bio-Rad, Hercules, CA, USA).

We performed a live/dead assay to assess the protective ability of sEVs. In brief, ADSCs were seeded in 24-well plates at 2 × 10^4^ cells/well density. PBS, nsEVs and hsEVs were suspended separately in complete DMEM/F12 medium and added into wells for 12 h. Then, ADSCs were changed to a serum-free medium with 300 µM H_2_O_2_ for 12 h. After the culture medium was discarded, PBS containing 2 µM calcein-AM (KeyGEN, Nanjing, Jiangsu, China) and 8 µM PI (KeyGEN) were added to the wells. Stained cells were observed in a fluorescence microscope (Nikon, Minato-ku, Tokyo, Japan). The survival rate was quantified by ImageJ software.

Cell apoptosis was analyzed using the FCM with an Annexin V-PE/7-AAD apoptosis detection kit (BD Biosciences, Franklin Lakes, NJ, USA). ADSCs were cultured in 6-well plates at a density of 1 × 10^5^ cells/well. After priming ADSCs with PBS, nsEVs, hsEVs and hsEVs + PX478 (25 µM) for 12 h, ADSCs were exposed to H_2_O_2_ stress at a concentration of 300 mM in a serum-free medium for 12 h. Then ADSCs were digested into single cells and stained by Annexin V-PE and 7-ADD following the manufacturer’s protocols.

### Western blotting assay

Total protein of ADSCs or sEVs was extracted with RIPA buffer (Beyotime, Shanghai, China) containing a cocktail of protease of phosphate inhibitors and quantified using a BCA protein assay kit. Samples (30 µg protein/lane) were separated by SDS-PAGE and then transferred onto polyvinylidene fluoride (PVDF, Millipore, Darmstadt, Germany) membrane. After being blocked with 5% non-fat milk for 1 h, the membranes were incubated with primary antibodies overnight at 4 ℃. Then membranes were incubated with secondary antibody for 1 h at room temperature. Finally, the signal was detected by enhanced chemiluminescence western blotting substrate (Millipore). The primary antibodies against CD63 (#ab134045), ALIX (#ab186429), GRP94 (#ab10860), were purchased from Abcam (Cambridge, United Kingdom); TSG101 (#28283-1-AP), HIF-1α(#20960-1-AP), CASPASE3 (#19677-1-ap), BAX (#50599-2-lg), BCL-2 (#12789-1-ap) were purchased from Proteintech (Wuhan, Hubei, China); β-actin (#4970), PTEN (#9188), AKT (#9272), p-AKT (#4060), MTOR (#2983), p-MTOR (#5536), 4EBP (#9452), p-4EBP (#9455), P70S6K (#9202), p-P70S6K (#97596) were purchased from Cell Signaling Technology (Boston, MA, USA). All the raw images of western blotting are listed in Additional file 7.

### RNA extraction and quantitative real-time PCR analysis (qRT–PCR)

Total RNA of cells and sEVs was extracted by TRIzol agent (Invitrogen) following the manufacturer’s protocols. mRNA was reverse-transcribed to complementary DNA (cDNA) using a Prime Script RT–PCR kit (Takara Bio, Dalian, China). qRT–PCR using SYBR-Green PCR Master Mix (Takara Bio) was performed on a CFX96 Real-Time PCR system (Bio-Rad) to calculate the relative expression of mRNA. For micro RNA (miRNA) detection, reverse-transcription and qRT–PCR were accomplished by an ALL-in-One miRNA qRT–PCR detection Kit (GeneCopoeia, Rockville, MD, USA). The gene expression level was calculated according to the 2^−ΔΔCt^ method and normalized to β-actin or U6 snRNA. The specific primers used in this paper are listed in Additional file 1: Table S1.

### Scratch wound healing assay

After preconditioning with different sEVs (20 µg/mL) and PX478 for 12 h, the cultural media of ADSCs were switched to serum-free DMEM/F12 for 24 h to collect conditioned medium (CM). HUVECs were seeded into 24-well plates and grown to a 90% confluence. A sterile plastic 200 µL micropipette was used to generate scratch wounds in the cell monolayers. The medium of HUVECs was changed to the collected CM for 24 h. Five fields were randomly chosen in each well. The wound area was quantified using ImageJ software 1.51.

### Matrigel tube formation assay

50 µL of thawed matrigel (Corning, NY, USA) was layered in a 96-well plate and incubated at 37℃ for 30 min to solidify. 1.5 × 10^4^ HUVECs were suspended in 100 µL CM and seeded on the matrigel-coated plate for 4 h. Photos were taken using an inverted microscope (Olympus), and the numbers of nodes/field were counted.

### Transfection

For PTEN overexpression, a lentivirus vector containing PTEN (pLV-CMV-PTEN-IRES-Puro) and a control lentivirus vector were constructed (Genechem, Shanghai, China). ADSCs were seeded on a 6-well plate in complete DMEM-F12 media, and lentivirus and HitransG were used according to the manufacturer’s instructions. For microRNA knockdown and overexpression, miR-486-5p mimics, inhibitors and their scramble control oligos were purchased from GenePharma (Shanghai, China). HUVECs (5 × 10^5^ cells/well) were seeded on 6-well plates the day before transfection. Each well received 75 pmol of microRNA mimics/inhibitor or control oligos and 5 µL of lipofectamine3000 (Invitrogen, Waltham, MA, USA). After 12 h of transfection, HUVECs were switched to sEVs depleted medium for sEVs collection. For tracking the survival of ADSCs in vivo, ADSCs were transfected with a self-inactivating lentiviral vector that carried the ubiquitin promoter driving firefly luciferase and enhanced green fluorescent protein double fusion reporter gene as previously reported.

### Dual-luciferase reporter assay

The fragments of the 3’ untranslated region (3′ UTR) of PTEN containing the putative binding sites of miR-486-5p or its mutant binding sites were synthesized and inserted in the pmirGLO vector. NC mimic or miR-486-5p mimic and wild type or mutant plasmids were co-transfected to HEK293. Forty-eight hours later, HEK293 was harvested, and relative luciferase activity was measured using Dual-Glo Luciferase Assay system (Promega, Fitchburg, WI, USA).

### Induction of hindlimb ischemia

Hindlimb ischemia was induced by left femoral artery resection using 6–8 week-old male BALB/c mice anesthetized by 1.5% isoflurane as previously reported. 1 × 10^6^ ADSCs suspended in 100 µL PBS was injected into the ischemic limb’s gastrocnemius (GC) muscle after ligation (n = 10 for each group). The same volume of PBS was injected as control.

### Bioluminescence imaging (BLI)

A Xenogen IVIS SPECTR imaging system (Xenogen Corporation, Hopkinto, MA, USA) was used to evaluate the survival of transplanted ADSCs^Fluc/eGFP^ in ischemic limb at Days 1, 3, 5, 7 and 10. Mice were anesthetized by isoflurane and injected with D-luciferin (150 mg/kg; Beyotime) intraperitoneally. After 10 min, the signal was captured by the IVIS system. BLI signals were measured by the average radiance of the region of interest (ROI) over the left hindlimb.

### Laser Doppler perfusion

Laser Doppler perfusion imager (LDPI) (Moor Instruments, Devon, United Kingdom) was used to monitor the blood recovery of the ischemic limb at Days 1, 3, 7, 14, 21 and 28. Mice were anesthetized by isoflurane and placed on a heating pad during scanning. Each mouse was scanned three times. Average blood flow values of ROI were calculated.

### Tissue histochemical and immunofluorescence staining

After euthanasia, GC muscle of the ischemic limb was harvested at Days 7, 14 and 28. Muscle was fixed in 4% paraformaldehyde and embedded in paraffin. 5 µm of sections were stained with hematoxylin and eosin (H&E) or Masson Staining Kit (Solarbio, Beijing, China). H&E staining was carried out to assess the cell degeneration, cell death and inflammatory infiltration. Masson’s trichrome staining measured the extent of fibrosis. ImageJ calculated the average collagen contents in five random fields. For immunofluorescence staining, tissue was embedded in the optimal cutting temperature (OCT) compound (Sakura Finetek, Tokyo, Japan) and snap-frozen. Frozen sections (6 µm) were blocked with goat serum at room temperature for 1 h, then incubated with CD31 antibody (1:500, Abcam) for another hour. After washing, sections were incubated with Alexa Fluor 594 labeled secondary antibody at room temperature for 1 h, followed by DAPI staining. To monitor the survival of ADSCs^Fluc/eGFP^, muscle tissues were cut into 6-µm sections and stained by DAPI. Images were captured by fluorescence microscopy (Olympus, Tokyo, Japan). 5 fields were randomly chosen, and ImageJ was used for quantification.

### Semiquantitative assessment of tissue damage and motor function

A scoring system was used to evaluate ischemic damage and motor ability of the hindlimbs. Tissue damage (6 = full recovery, 5 = minor necrosis or nail loss, 4 = partial toe amputation, 3 = total toe amputation, 2 = partial/total foot amputation, 1 = partial/total limb amputation) and motor function (5 = unrestricted, 4 = no active use of toe or spreading, 3 = restricted foot use, 2 = no use of foot, 1 = no use of limb) were graded at postoperative Day 0, 1, 7, 14, 21 and 28. 10 mice for each group [33], scores were calculated by GraphPad 8.0. Results were analyzed by Mann–Whitney test.

### Statistical analysis

All results presented were conducted at least three independent experiments. Statistical analysis was performed on GraphPad Prism 8.0. One-way ANOVA and Student’s t-test were used to determine the significance of different groups. Data are expressed as mean ± SEM, with *p < 0.05 being seen as significant.

## Results

### The effects of the HUVEC secretome on ADSCs

**Fig. 1:**
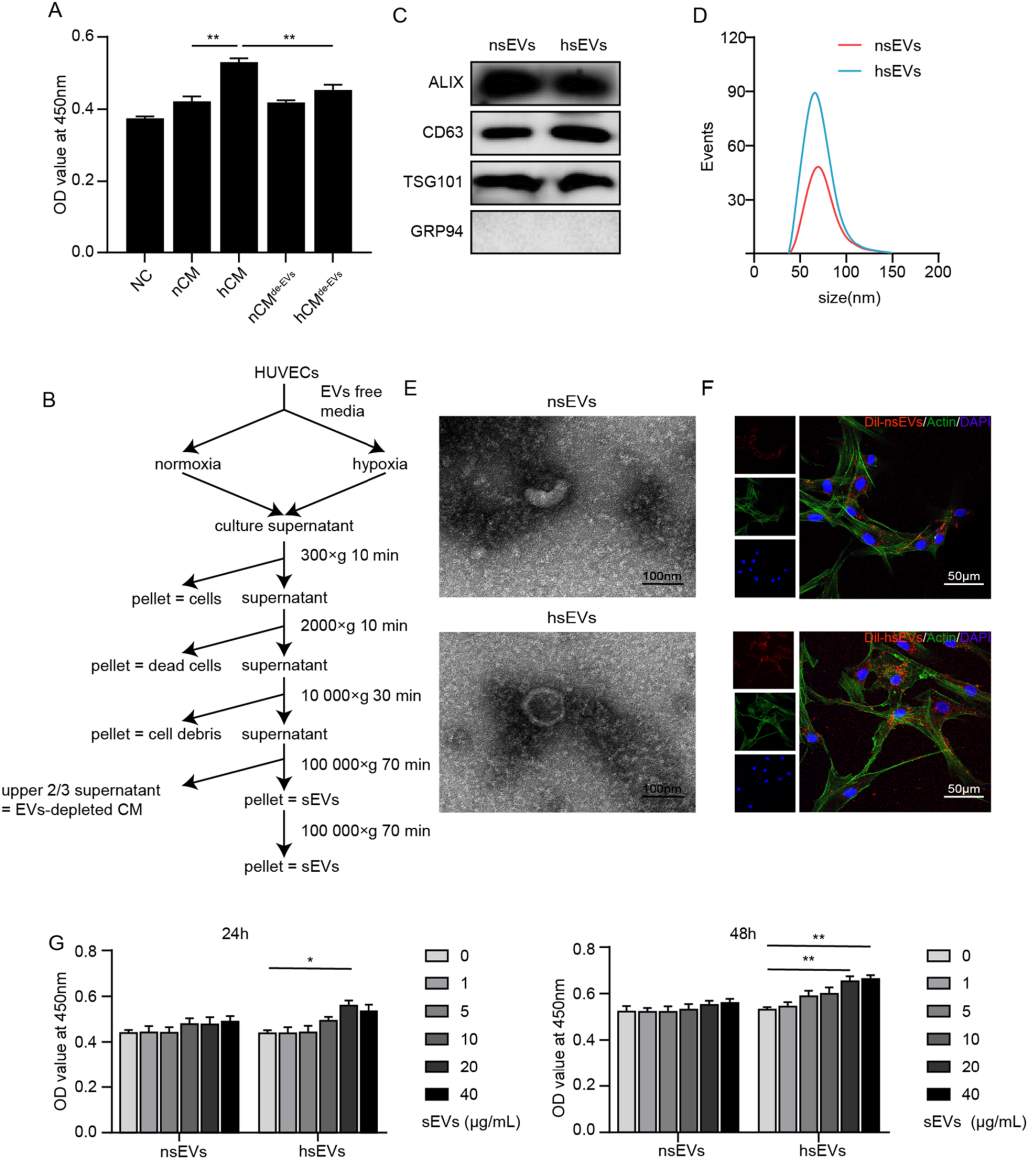
The HUVEC secretome increased the proliferation of ADSCs. **A** Cell proliferation of ADSCs was detected by CCK8 assay. **B** Procedure of sEVs isolation and sEVs-depleted CM collection. **C** The expression of ALIX, CD63, TSG101 and GRP94 in nsEVs and hsEVs was analyzed by western blotting. **D** The size distribution of nsEVs and hsEVs was measured by NanoFCM. **E** TEM was used to detect the morphology of nsEVs and hsEVs. Scale bars, 100 nm. **F** The internalization of Dil-labeled nsEVs and Dil-labeled hsEVs by ADSCs was observed under fluorescence microscopy. Scale bars, 50 µm. **G** Proliferation of ADSCs affected by the concentrations of nsEVs and hsEVs was measured by CCK8 assay. All data are representative of three independent experiments and are shown as the mean ± SEM. (n = 3; *P < 0.05; **P < 0.01)

The isolated ADSCs showed fibroblast-like and spindle morphology (see Additional file 2: Fig. S1A). As shown in Additional file 2: Fig. S1B, FCM demonstrated that ADSCs were negative for hematopoietic markers (CD34, CD45) but positive for MSC surface markers (CD29, CD44, CD90, CD105) [34]. It was previously found that the secretome of HUVECs could promote ADSC proliferation [35]. Therefore, we collected conditioned media from HUVECs under normoxia and hypoxia conditions [36]. No severe cell death was observed during hypoxia exposure. ADSCs were switched to the CM of HUVECs. Through the CCK-8 assay (Fig. 1A), normoxic CM (nCM) facilitated ADSC proliferation, while hypoxic CM (hCM) further promoted the proliferation of ADSCs compared to the nCM group. Since hypoxia has been reported to increase the production of sEVs from HUVCEs [37], we further explored whether sEVs played a role in the pro-proliferative activity. The CM of both groups was subjected to ultracentrifugation to deplete EVs, and nCM^EV−de^ and hCM^EV−de^ were obtained (Fig. 1B). The pro-proliferative effect of hCM was significantly compromised by EV depletion (Fig. 1A), which indicated that hypoxic EVs(hEVs) might play an essential role in the proliferation of ADSCs.

### Isolation and characterization of sEVs

To further illustrate the effects of endothelial sEVs, we isolated sEVs from HUVECs under normoxia and hypoxia conditions by ultracentrifugation (Fig. 1B) [38]. Western blotting verified that both groups of sEVs contained the well-established sEVs markers ALIX, CD63 and TSG101 but did not contain the negative marker GRP94 (Fig. 1C). Flow nanoanalysis showed that the average diameter of nsEVs and hsEVs was approximately 70 nm (Fig. 1D). Furthermore, sEVs showed a cup-shaped structure in TEM images (Fig. 1E). To confirm the internalization of sEVs by ADSCs, we labeled sEVs with Dil, a lipophilic fluorescent dye that stains the exosome membrane. After incubation with Dil-sEVs (20 µg/mL) for 12 h, ADSCs successfully internalized Dil-sEVs into the cytoplasm (Fig. 1F). A cell proliferation assay revealed that hsEVs promoted ADSC proliferation in a concentration-dependent manner at 24 h and 48 h, while this effect was not observed in the nsEVs group (Fig. 1G).

### The anti-apoptosis potential of sEVs in vitro

**Fig. 2:**
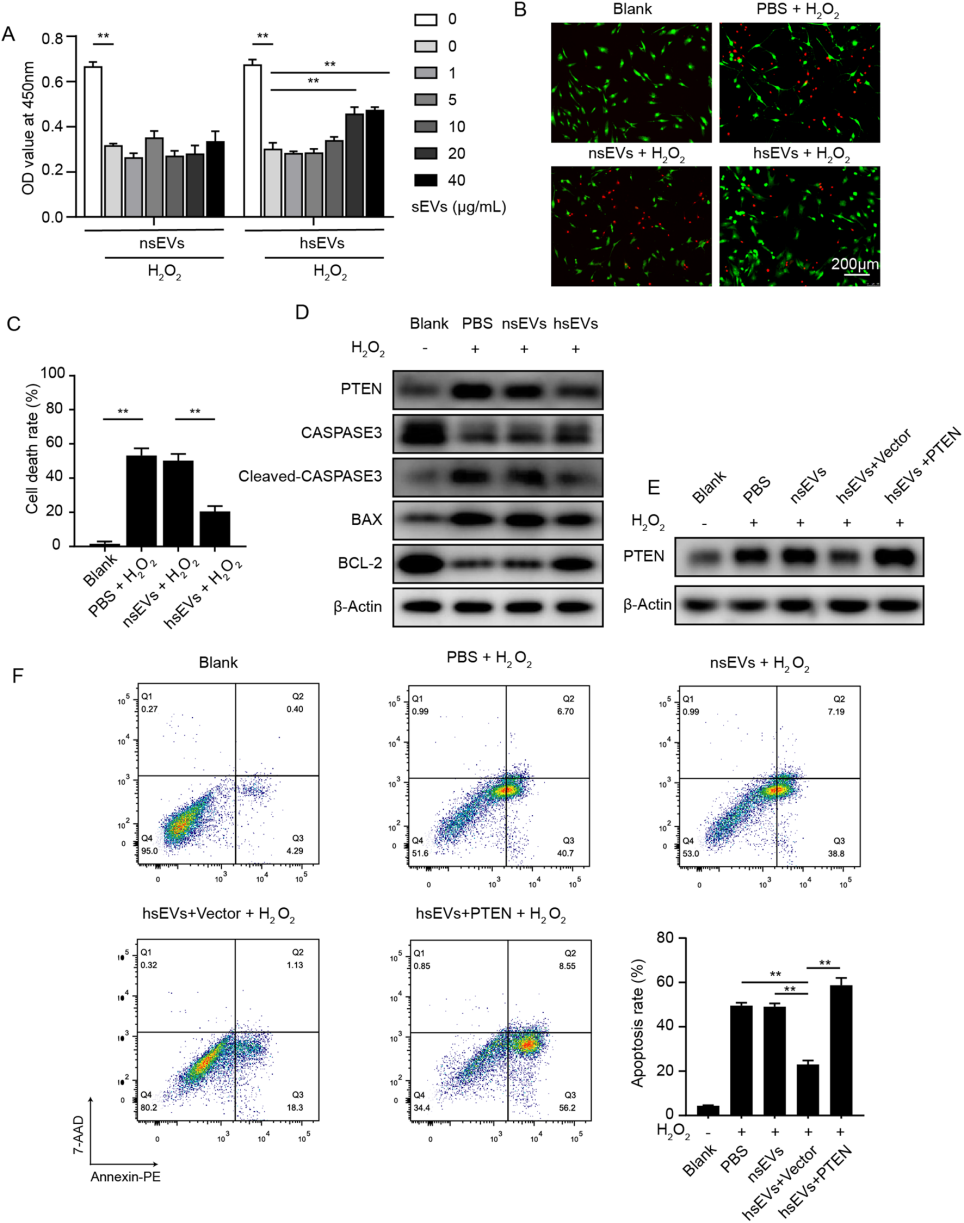
hsEVs attenuated ADSC cell death caused by H_2_O_2_ stress. **A** A CCK8 assay was conducted to detect the viability of ADSCs in the presence of different concentrations of nsEVs and hsEVs under exposure to 300 µM of H_2_O_2_ for 12 h. **B** A live/dead assay was performed to detect cell death of ADSCs under ROS pressure after preconditioned by PBS, nsEVs (20 µg/mL) and hsEVs (20 µg/mL). **C** Quantitative analysis of the live/dead assay performed in **B**. **D** The protein level was detected in ADSCs, ADSCs under H_2_O_2_-induced stress, ADSCs preconditioned by nsEVs or hsEVs for 12 h and then exposed to H_2_O_2_ (300 nM). **E** The expression of PTEN was detected by western blotting. **F** The apoptotic rate of ADSCs was measured by FCM. All data are representative of three independent experiments and are shown as the mean ± SEM. (n = 3; *P < 0.05; **P < 0.01).

ADSCs encounter hostile environments, such as hypoxia, nutrient deprivation and excessive ROS, when they are injected into ischemic muscle. Recently, researchers reported that sEVs from HUVECs could serve as a new therapeutic agent in the treatment of ischemia–reperfusion injury of cardiomyocytes by reducing cell death [23]. To examine whether sEVs from HUVECs exhibit cell-protective effects against ADSCs, ADSCs were pretreated with nsEVs or hsEVs and then exposed to H_2_O_2_ (300 µM) for 12 h. Cell viability was detected by CCK8 assay. H_2_O_2_ exposure significantly reduced the viability of ADSCs, indicative of successful ROS insult (Fig. 2A). Interestingly, hsEVs attenuated H_2_O_2_-induced cell death of ADSCs, and the peak concentration was 20 µg/mL (Fig. 2A). This concentration of sEVs was used in the following experiments. The live/dead assay showed similar results (Fig. 2B, C). Compared to the blank group, H_2_O_2_ caused a dramatic increase in the proportion of red cells (dead cells); however, hsEVs decreased the proportion of red cells, but nsEVs did not alter the proportion of dead cells. To further explore whether hsEVs reduce the cell death of ADSCs by alleviating apoptosis, the expression of apoptosis-related proteins, including PTEN, CASP3, BAX and BCL2, was examined. Anti-apoptosis proteins, such as BCL2, were downregulated by ROS exposure (Fig. 2D) but elevated by hsEVs preconditioning. Likewise, the expression of proapoptotic proteins, such as PTEN, cleaved-CAPS3 and BAX, was decreased by hsEVs priming.

PTEN is a lipid phosphatase that catalyzes phosphatidylinositol 3,4,5-trisphosphate [PI (3,4,5) P_3_] into phosphatidylinositol 3,4-bisphosphate [PI (3,4) P_2_]; therefore, PTEN counteracts PI3K and is seen as an antagonist of PI3K [39]. A previous report emphasized that the PI3K pathway influenced the survival, angiogenesis and oxidative stress of MSCs in ischemic environment [40]. Next, to identify whether PTEN is necessary for the anti-apoptosis effects of hsEVs, we performed lentivirus-mediated PTEN overexpression in ADSCs. Western blotting analysis confirmed the successful overexpression of PTEN (Fig. 2E). We used FCM to evaluate the extent of apoptosis (early apoptosis and late apoptosis). As shown in Fig. 2F, H_2_O_2_ significantly increased the proportion of apoptotic cells to approximately 45%, while hsEVs preconditioning decreased the proportion of apoptotic cells to approximately 20%. Additionally, PTEN overexpression in ADSCs reversed the anti-apoptosis potential of hsEVs. Therefore, we speculated that the hsEVs-mediated anti-apoptosis effects are related to hsEVs-induced PTEN repression.

### hsEVs upregulated the expression of HIF-1α through the AKT/MTOR pathway

**Fig. 3:**
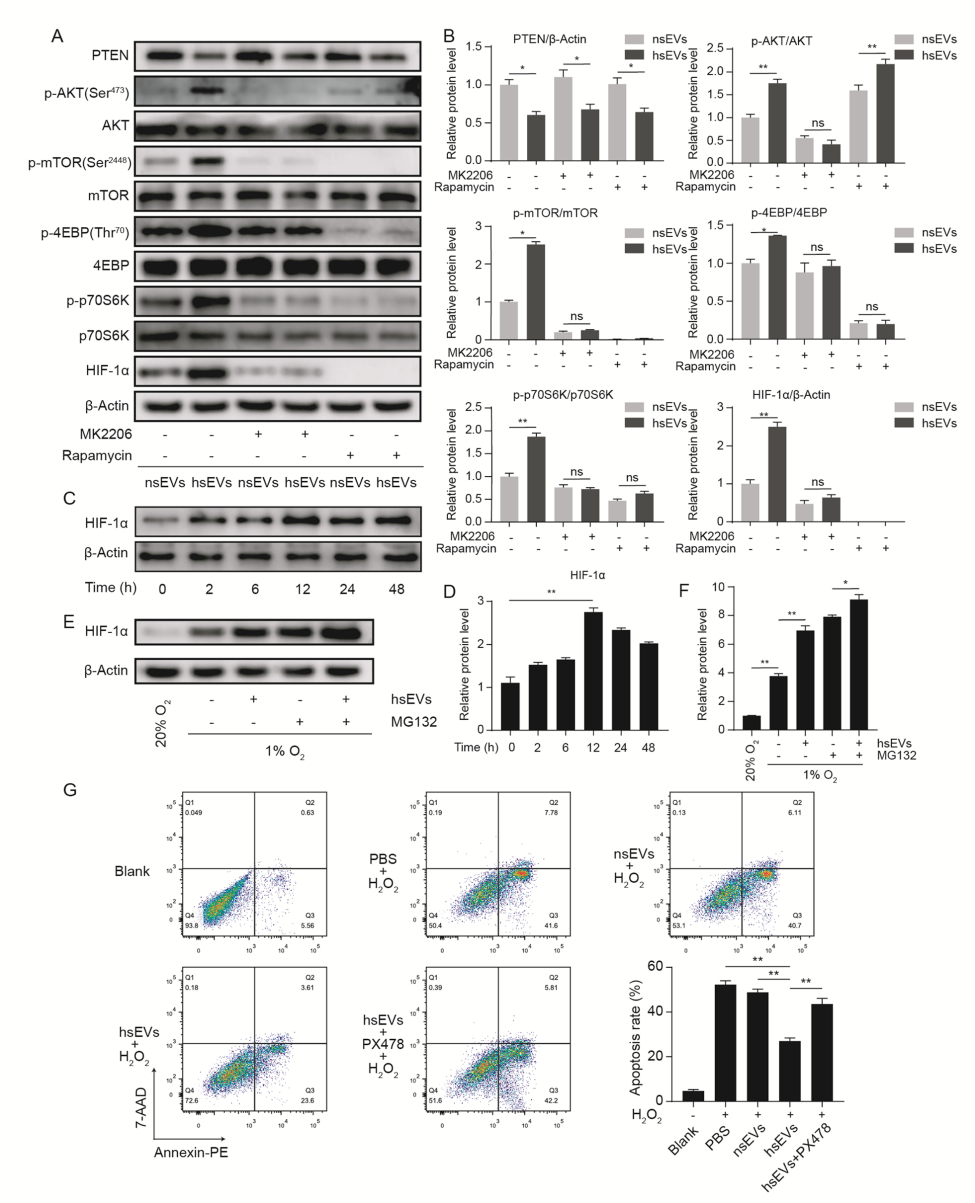
hsEVs activated the AKT/MTOR pathway by PTEN inhibition. **A**, **B** ADSCs were incubated with nsEVs (20 µg/mL), hsEVs (20 µg/mL), MK2206 (5 µM) and rapamycin (15 µM) as indicated for 12 h, and total proteins were collected for western blotting. The protein levels of PTEN, p-AKT, AKT, p-MTOR, MTOR, p-4EBP, 4EBP, p-p70S6K, p70S6K, HIF-1α and β-actin were determined. **C**, **D** ADSCs were harvested at the indicated times for western blotting to detect HIF-1α expression. **E**, **F** ADSCs were cultured with hsEVs (20 µg/mL) and/or MG132 (10 µM) under normoxia (21% O_2_) or hypoxia (1% O_2_) for 12 h, and then, proteins were harvested for western blotting to detect HIF-1α expression. **G** ADSCs were fed PBS, nsEVs (20 µg/mL), hsEVs (20 µg/mL) and hEV + PX478 (25 µM) for 12 h and then cultured with medium containing H_2_O_2_ (300 nM) for another 12 h. Cell apoptosis was measured by FCM. All data are representative of three independent experiments and are shown as the mean ± SEM. (n = 3; *P < 0.05; **P < 0.01).

In Fig. 3A, B, western blotting confirmed that hsEVs inhibited PTEN and upregulated p-AKT, while the AKT inhibitor MK2206 successfully inhibited the phosphorylation of AKT by hsEVs. p-MTOR was also upregulated by hsEVs; however, the phosphorylation of MTOR in ADSCs was significantly attenuated in response to hsEVs in the presence of MK2206, which indicated that the upregulation of p-MTOR was AKT dependent. Additionally, the phosphorylation of the downstream molecules 4EBP and p70S6K was also upregulated in hsEVs-treated ADSCs. Similarly, their upregulation was abrogated by MK2206 (Lane 3 compared to Lane 4) and rapamycin (lane 5 compared to lane 6). Furthermore, in parallel with the phosphorylation of AKT, MTOR, 4EBP and p70S6K, the expression of hypoxia inducible factor 1 subunit alpha (HIF-1α) was increased following hsEVs treatment in ADSCs, while MK2206 and rapamycin completely abrogated the hsEVs-induced upregulation of HIF-1α.

We also found that the elevation of HIF-α was time-dependent and peaked at 12 h (Fig. 3C, D). However, qRT–PCR showed that hsEVs did not elevate the mRNA level of HIF-1α compared to PBS or nsEVs (see Additional file 3: Fig. S2A). The level of HIF-1α is influenced by protein synthesis and protein degradation, and the degradation of HIF-1α is mainly affected by oxygen levels [41]. To explore whether hsEVs affect HIF-1α expression through protein synthesis and degradation, ADSCs were treated with hsEVs and MG132 as indicated. In Fig. 3E, F, hsEVs could still increase the expression of HIF-1α in ADSCs under hypoxia (Lane 2 compared to Lane 3). Then, we used MG132 to block the proteolytic activity of the proteasome complex. Interestingly, hsEVs still increased the level of HIF-1α under the synergistic effect of MG132 and hypoxia (Lane 4 compared to Lane 5). These results indicated that hsEVs enhanced the de novo synthesis of HIF-1α. It has been reported that activation of 4EBP and p70S6K results in elevation of protein translation [42]. Taken together, we found that hsEVs activated the AKT/MTOR signaling pathway, hence increasing the synthesis of HIF-1α. It has been reported that HIF-1α overexpression in ADSCs could enhance their survival and paracrine function in vitro and in vivo [43]. Therefore, we hypothesized that HIF-1α played an important role in hsEVs-mediated cell protection. We used a HIF-1α inhibitor, PX478, to test this hypothesis. Figure 3G demonstrated that H_2_O_2_ caused a significant increase in the rate of apoptosis in ADSCs. The proportion of apoptotic cells decreased following hsEVs treatment, but PX478 significantly attenuated the protective effect of hsEVs. The inhibitory efficiency of PX478 on HIF-1α was examined by western blotting (see Additional file 3: Fig. S2B). These findings implied that hsEVs protected ADSCs from apoptosis induced by H_2_O_2_ and that the AKT/MTOR/HIF-1α pathway played a critical role in these effects.

### hsEVs enhanced the proangiogenic ability of ADSCs

**Fig. 4:**
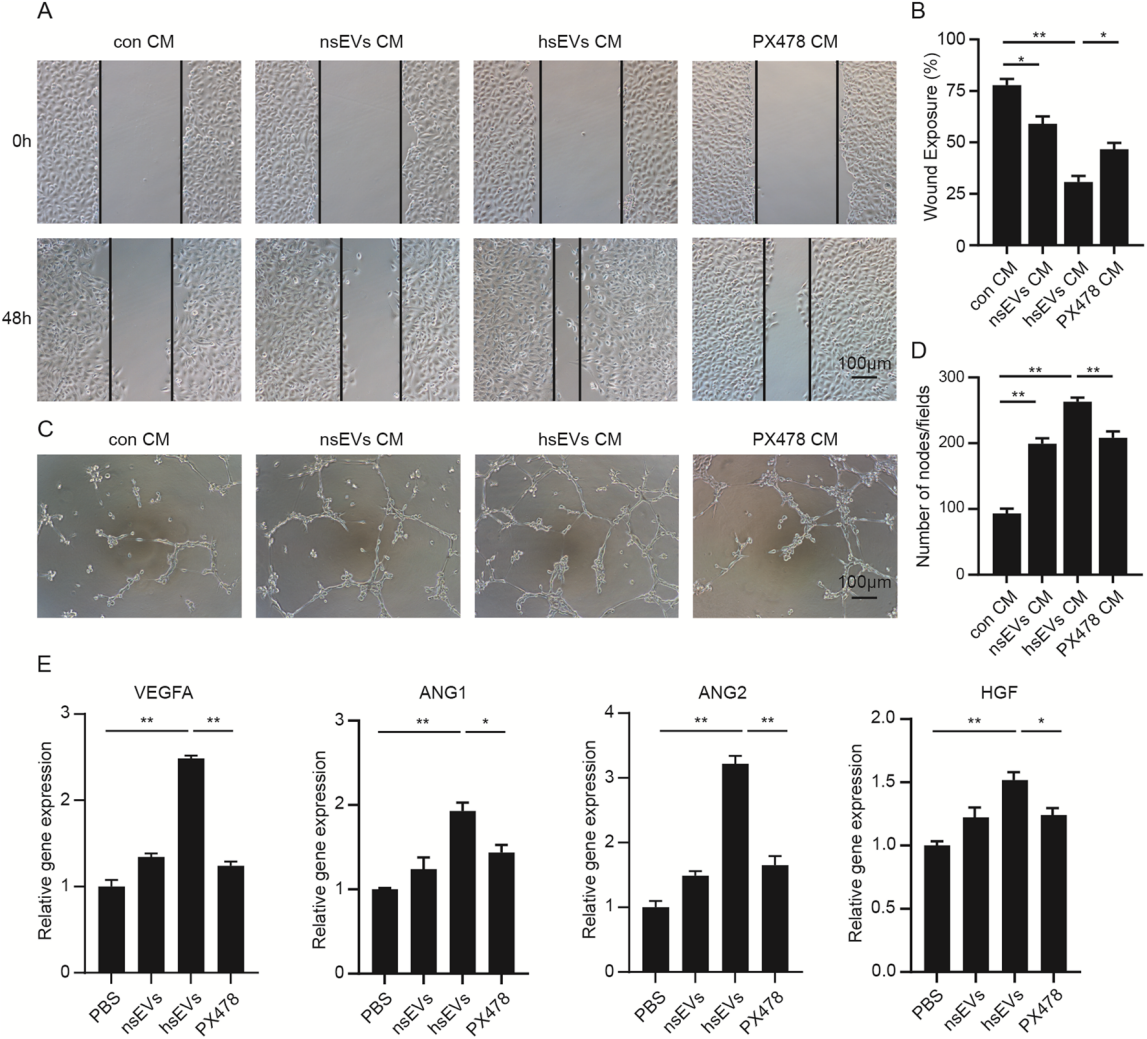
hsEVs promoted the proangiogenic potential of ADSCs. **A** A scratch assay was performed to assess the effects of different ADSCs CM on the migratory ability of endothelial cells. **B** Quantitative analysis of the scratch assay. **C** Representative images of the matrigel tube formation assay of endothelial cells treated with different groups of CM from ADSCs. **E** Quantitative analysis of the matrigel tube formation assay. **F** The expression of angiogenesis-related genes was examined by qRT–PCR. Relative gene expression was calculated by the 2^−ΔΔCt^ method and normalized to β-actin. All data are representative of three independent experiments and are shown as the mean ± SEM. (n = 3; *P < 0.05; **P < 0.01).

ADSCs can secrete angiogenic cytokines to promote angiogenesis of endothelial cells [44], and HIF-1α is a well-known transcription factor of a series of angiogenic factors. Hence, we decided to test the effects of hsEVs on the proangiogenic ability of ADSCs. The conditioned media from ADSCs (con CM), nsEVs (20 µg/mL)-primed ADSCs (nsEVs CM), hsEVs (20 µg/mL)-primed ADSCs (hsEVs CM), and hsEVs (20 µg/mL) + PX478 (20 nM)-primed ADSCs (PX478 CM) were collected. Then, we performed scratch and tube formation assays by culturing HUVECs with conditioned media. After culturing with conditioned media for 24 h, the nsEVs CM (59.0%) and hsEVs CM (30.7%) groups showed lower wound exposure ratio than the con CM (77.8%) group (Fig. 4A, B). However, PX478 partly reversed the pro-migratory ability of hsEVs CM. Similar results were also observed in the tube formation assay; endothelial cells cultured with nsEVs CM and hsEVs CM formed more capillary-like structures on matrigel (Fig. 4C, D), while PX-478 decreased the number of nodes (Fig. 4E). To further explore the underlying mechanism, the expression of proangiogenic cytokines in ADSCs was detected by qRT–PCR. As expected, hsEVs treatment significantly upregulated the expression of the proangiogenic genes vascular endothelial growth Factor A (VEGFA), angiopoietin 1 (ANG1), angiopoietin 2 (ANG2), and hepatocyte growth factor (HGF), while the upregulation of these genes was decreased by PX478 treatment (Fig. 4E). In summary, hsEVs enhanced the proangiogenic ability of ADSCs where HIF-1α accumulation increased the expression of angiogenesis-related cytokines.

### hsEVs conferred protection and proangiogenic effects by supplementing miR-486-5p

**Fig. 5:**
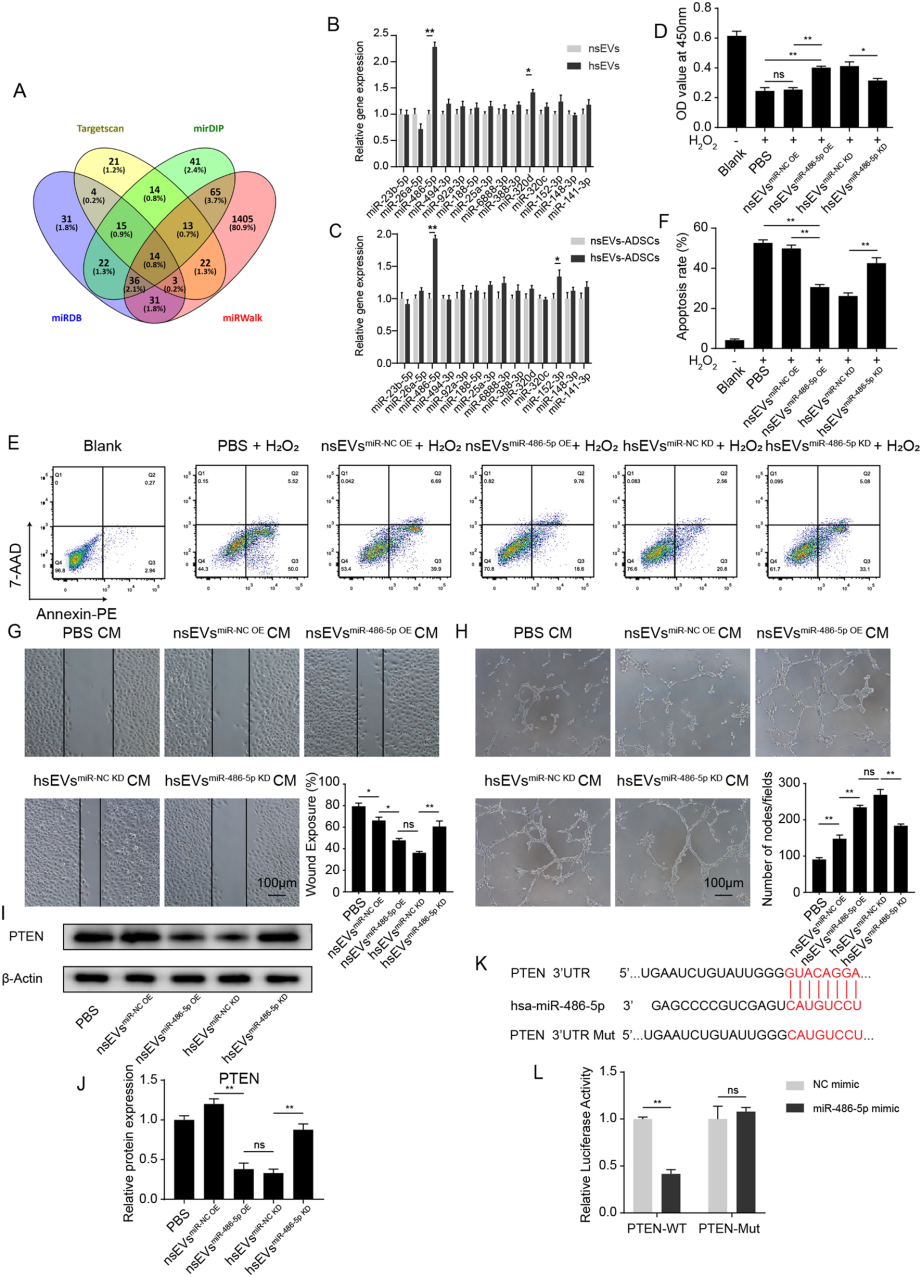
miR-486-5p meditated the cell protective and proangiogenic effects of hsEVs. **A** Venn diagram of the potential miRNAs targeting PTEN. **B** Quantification of a series of miRs in nsEVs and hsEVs by qRT–PCR. **C** Expression of miR in ADSCs treated with nsEVs or hsEVs was measured by qRT–PCR. **D** The viability of ADSCs was monitored by CCK8 assay. **E**, **F** Apoptosis rates of ADSCs under ROS exposure were measured by FCM. **G** A scratch assay of endothelial cells was conducted in the presence of different CM from ADSCs for 48 h. **H** Representative phase-contrast images of the matrigel tube formation assay. **I**, **J** Relative expression of PTEN in ADSCs was analyzed using western blotting. **K** MiR-486-5p regulated PTEN by directly targeting the 3′-UTR. **L** A dual Luciferase reporter containing wild-type or mutant PTEN 3′UTR and scramble microRNA (mimic-NC) or miR-486-5p mimic was co-transfected into HEK293 cells. All data are representative of three independent experiments and are shown as the mean ± SEM. (n = 3; *P < 0.05; **P < 0.01).

As sEVs may act as a source of exogenous microRNAs (miRNAs), we used several in silico tools (TargetScan, miRDB, mirDIP and miRWalk) to predict the miRNAs targeting PTEN. Fourteen miRNAs were identified (Fig. 5A). To identify whether hsEVs influenced the level of PTEN through shuffling miRNAs, the levels of these miRNAs in nsEVs and hsEVs were examined. qRT–PCR showed that a series of miRNAs were enriched in hsEVs, especially miR-486-5p and miR-320d (Fig. 5B). Next, we examined the difference in miRNAs in ADSCs after nsEVs or hsEVs treatment. Compared to the nsEVs group, hsEVs upregulated the expression of miR-486-5p and miR-152-3p (Fig. 5C). These results indicated the potential transfer of miR-486-5p from hsEVs to ADSCs.

Next, we investigated whether miR-486-5p contributed to the inhibition of PTEN and the protective effects of hsEVs. sEVs were isolated from HUVECs under normoxia that had been treated with either a scrambled oligonucleotide sequence (nsEVs^miR−NC OE^) or a miR-486-5p mimic (nsEVs^miR−486−5p OE^). In addition, sEVs were isolated from HUVECs under hypoxia that had been treated with either a scrambled oligonucleotide sequence (hsEVs^miR−NC KD^) or a miR-486-5p inhibitor (hsEVs^miR−486−5p KD^) (see Additional file 4: Fig. S3A). Additionally, the expression of miR-486-5p in sEVs-treated ADSCs was also measured by qRT–PCR (see Additional file 4: Fig. S3B). Then, in the presence of H_2_O_2_ (300 mM), the viability of ADSCs that were primed with PBS or nsEVs^miR−NC OE^, nsEVs^miR−486−5p OE^, hsEVs^miR−NC KD^, or hsEVs^miR−486−5p KD^ was tested (Fig. 5D). Compared to the blank group, H_2_O_2_ significantly decreased the cell viability of ADSCs. Similar to previous results, hsEVs^miR−NC KD^ instead of nsEVs^miR−NC OE^ showed cell protective effects in the CCK8 assay. Interestingly, protective effects were also observed in cells treated with nsEVs^miR−486−5p−OE^, but miR-486-5p knockdown attenuated the protective effects of hsEVs. The results from the FCM assay were consistent with the CCK8 assay, and nsEVs^miR−486−5p OE^ and hsEVs^miR−NC KD^ reduced the apoptotic rate of ADSCs (Fig. 5E, F). These data indicated that downregulation of miR-486-5p could significantly decrease the effect of hsEVs in conferring cell protection.

It is still unknown whether miR-486-5p is essential for mediating the proangiogenic ability of ADSCs. Therefore, we conducted scratch and tube formation assays by culturing HUVECs with conditioned medium from ADSCs that were treated with PBS, nsEVs^miR−NC OE^, nsEVs^miR−486−5p OE^, hsEVs^miR−NC KD^ or hsEVs^miR−486−5p KD^. We observed that wound exposure was significantly reduced in HUVECs cultured with medium from ADSCs treated with nsEVs^miR−486−5p OE^ or hsEVs^miR−NC KD^, and wound exposure increased when HUVECs were incubated with medium from ADSCs treated with hsEVs^miR−486−5p KD^ (Fig. 5G; Additional file 5: Fig. S4A). In the tube formation assay, the nsEVs^miR−486−5p OE^ and hsEVs^miR−NC^ groups formed more tube structures (Fig. 5H). The above results emphasized the importance of miR-486-5p in the proangiogenic effects in hsEVs.

In addition, Fig. 5I, J show that compared to the nsEVs^miR−NC OE^ group, miR-486-5p overexpression decreased the expression of PTEN. In contrast, miR-486-5p knockdown diminished the inhibitory effects of hsEVs on the expression of PTEN. These results indicated a negative correlation between miR-486-5p and PTEN. Next, we wanted to verify the direct interaction of miR-486-5p and the 3′ UTR of PTEN mRNA. Bioinformatics revealed a putative miR-486-5p binding site in the 3’ UTR of PTEN mRNA. Then, fragments of wild-type forms of the putative binding site of the 3’ UTR (WT) or their mutant forms (Mut) were synthesized and cloned into the pmirGLO vector (Fig. 5K). A dual luciferase reporter assay showed that the miR-486-5p mimic decreased the luminescence of the WT plasmid group (Fig. 5L). However, neither miR-486-5p nor the NC mimic interfered with the relative luminescence in the Mut plasmid transfection group. These results demonstrated that PTEN is a direct target of miR-486-5p.

### hsEVs enhanced the engraftment of ADSCs

**Fig. 6:**
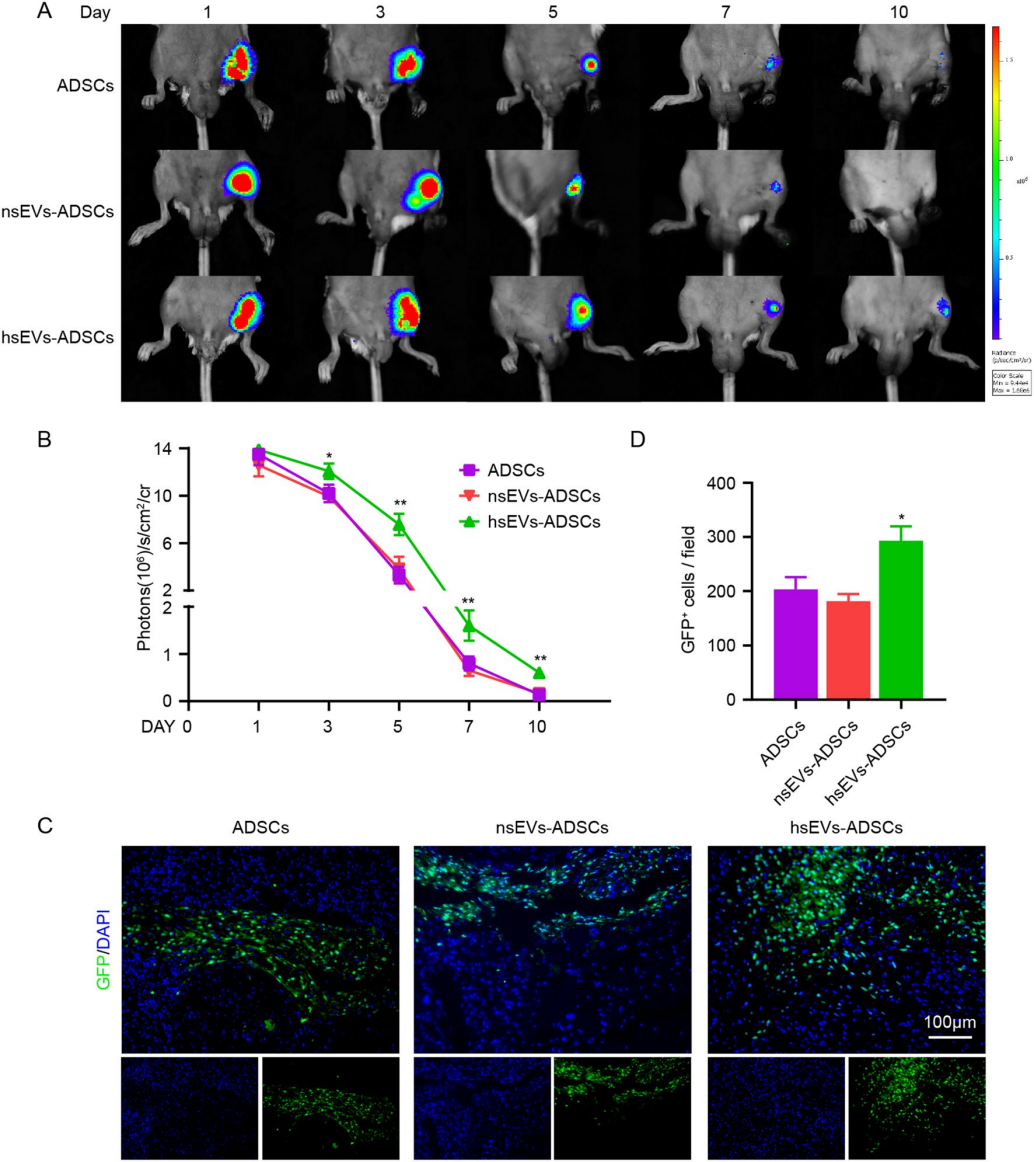
hsEVs improved the survival of ADSCs in ischemic muscles. **A** The survival of ADSCs^Fluc/eGFP^ primed with PBS, nsEVs and hsEVs was detected by BLI. D-Luciferin potassium salt was injected intraperitoneally. **B** Quantification of BLI signals. **C** ADSCs^Fluc/eGFP^ were injected into ischemic limbs; representative photomicrographs of ischemic GC muscle at Day 7. Scale bars, 100 µm. **D** Quantification of eGFP^+^ cells in photomicrographs of tissue sections. Data are expressed as the mean ± SEM. (n = 5. ^∗^P < 0.05 versus ADSCs; ^∗∗^P < 0.01 versus ADSCs.

To investigate whether hsEVs could improve the survival of ADSCs in vivo, ADSCs were transfected with a lentivirus that expresses firefly luciferase and eGFP. Fluorescence microscopy images showed that more than 95% of ADSCs were eGFP positive (eGPF +) (see Additional file 6: Fig. S5A). After the limb ischemia model was established, control, hsEVs-treated and nsEVs-treated ADSCs^Fluc/eGFP^ were injected into ischemic muscles. The survival of ADSCs^Fluc/eGFP^ was monitored longitudinally by BLI at Days 1, 3, 5, 7 and 10. BLI showed a robust signal in the ischemic limb region at Day 1 after ADSCs^Fluc/eGFP^ injection in all three groups (Fig. 6A, B). Serial BLI assays showed that hsEVs treatment improved the survival of ADSCs in vivo. Immunofluorescence staining of GC muscle on Day 7 postsurgery confirmed better engraftment of hsEVs-ADSCs (Fig. 6C, D), which was consistent with the results of the BLI assay. These results indicated that hsEVs treatment improved the cell viability and engraftment of ADSCs^Fluc/eGFP^ in ischemic muscle compared to PBS and nsEVs treatment.

### hsEVs augmented the therapeutic effects of ADSCs in a limb ischemia model

**Fig. 7:**
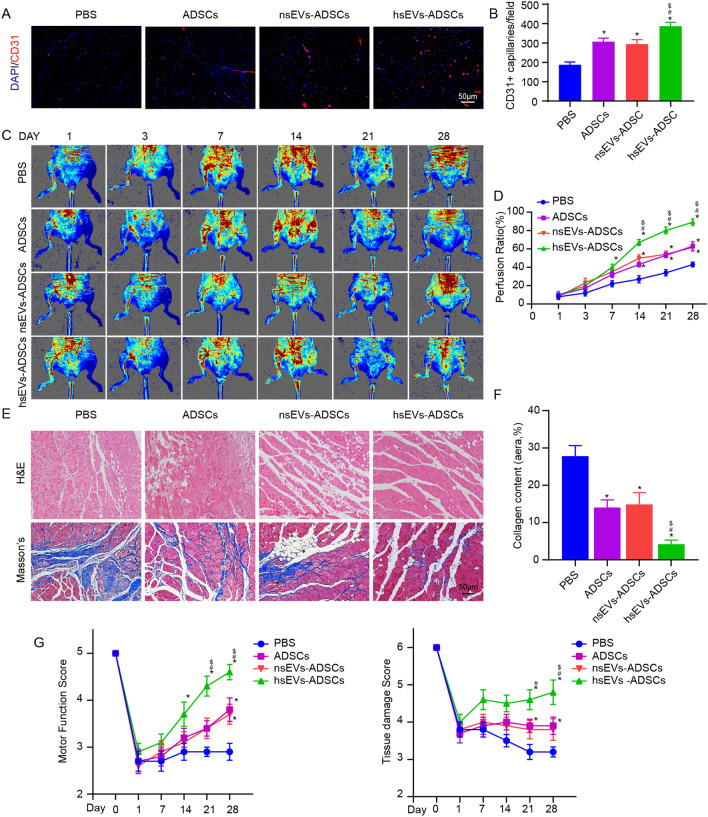
hsEVs enhanced the therapeutic effects of ADSCs in CLI. **A** Representative images of GC muscle stained for CD31 (red) and DAPI (blue). **B** Quantitative analysis of the capillary density in each group. **C** Laser Doppler perfusion image of ischemic hindlimb as indicated time after surgery. **D** Quantitation of perfusion recovery of the hindlimb. **E** Representative images of H&E staining and Masson’s staining of GC muscle sections in each group. **F** Collagen content (blue) via Masson’s trichrome staining of the GC muscle was calculated. **G** Tissue damage and motor function scores of the various treatment groups over time. Data are expressed as the mean ± SEM. (n = 5. *P < 0.05 versus PBS; ^#^P < 0.05 versus ADSCs; ^$^P < 0.05 versus hsEVs-ADSCs).

To observe angiogenesis in ischemic limbs, GC muscle was harvested from each group at 14 days postsurgery. CD31 immunofluorescence staining showed that hsEVs-ADSCs significantly increased the microvascular density in GC muscle (Fig. 7A, B). Serial LDPI monitored the blood flow recovery of ischemic limbs at Day 1, 3, 7, 14, 21 and 28 postsurgery (Fig. 7C). Blood flow of ischemic limbs was normalized to normal limbs. According to LDPI data, the hEV-ADSCs group showed the strongest blood perfusion signal at day 7, 14, 21 and 28 postsurgery, indicating that hsEVs increased the proangiogenic potential of ADSCs in the limb ischemia model (Fig. 7D).

To further assess the therapeutic effects of ADSCs, mice were sacrificed at Day 14 postsurgery. GC muscle was harvested to perform H&E staining. Light microscopic examinations illustrated that the muscle of the hsEVs-ADSCs group showed less multicellular infiltration and muscle fibers devoid of nuclei (Fig. 7E). Masson’s trichrome staining on GC muscle at Day 45 demonstrated that ADSCs and nsEVs-ADSCs significantly decreased the content of collagen fiber, while hsEVs-ADSCs further reduced the level of fibrosis (Fig. 7E, F).

Finally, serial semiquantitative assessment of tissue damage and motor function was performed on Day 1, 7, 14, 21 and 28 postsurgery. Both the tissue damage score and motor function score implied the best recovery from limb ischemia in the hsEVs-ADSCs group (Fig. 7G).

## Discussion

The present study employed sEVs isolated from hypoxic endothelial cells to stimulate ADSCs and improve the potential of ADSCs in the treatment of CLI. After incubation with hsEVs, ADSCs not only manifested stronger resistance to ROS stress, but also showed enhanced proangiogenic capacity, and these effects were partly attributed to the transfer of miR-486-5p from hsEVs. By targeting PTEN, miR-486-5p activated the AKT pathway and increased the synthesis of HIF-1α, leading to better cell survival and proangiogenic ability in vitro and in vivo. As a result, hsEVs-ADSCs presented superior neovascularization and tissue recovery in the limb ischemia model.

CLI is a chronic ischemic disease that occurs in the lower extremities and is characterized by ischemic rest pain, tissue loss and gangrene. Autologous ADSC therapy has emerged as a promising technique to improve pain scale and wound healing for “no-option” patients [45]. Early run-off of transplanted cells caused by a harsh microenvironment compromises the therapeutic effects of stem cells. Therefore, preconditioning strategies were used to improve the survival of ADSCs. For instance, IFN-γ-treated ADSCs exhibited enhanced immunosuppressive properties through IDO overexpression [18]. Here, we found that hsEVs of HUVECs, as a mediator, could make ADSCs better prepared for upcoming ROS exposure, and also enhance their proangiogenic ability.

A heart-on-chip study revealed that both hsEVs and nsEVs displayed a protective effect against ischemia/reperfusion damage in cardiomyocytes [23]. However, the present study discovered that only hypoxic sEVs of HUVECs made ADSCs more tolerant to ROS-induced apoptosis. The discrepancy indicated that sEVs exhibited different functionalities in different contexts. Other researchers found that sEVs of HUVECs produced under ischemia/reperfusion could protect SH-SYY nerve cells from apoptosis [26, 28, 46], but sEVs secreted from normal HUVECs did not influence cell apoptosis, which is similar to the present study. One study emphasized that stress of endothelial cells was reflected in the sEVs and altered the composition of the sEVs, which could result in different biological functions [47]. Additionally, different functions of endothelial sEVs produced under different environments have been reported extensively [27]. Moreover, we first demonstrated that both hsEVs and nsEVs were sufficient to increase the expression of angiogenesis-related cytokines in ADSCs, hence increasing their proangiogenic ability. The current study found that hsEVs induced PTEN inhibition and AKT activation. It is not surprising that hsEVs reduced the apoptosis of ADSCs, since PTEN and AKT are apoptosis-related proteins. We also noticed that it caused the activation of MTOR, therefore increasing the de novo synthesis of HIF-1α. HIF-1α is a master regulator of cellular adaptation to hypoxia, and its activation influences the expression of genes involved in angiogenesis, metabolism and survival [48, 49]. Using PX478, a HIF-1α inhibitor, our results provide compelling evidence that HIF-1α plays a pivotal role in protective and proangiogenic abilities. However, nsEVs also increased the proangiogenic potential of ADSCs without altering the expression of HIF-1α. Furthermore, compared to PBS treatment, PX478 did not completely abolish the proangiogenic ability of hsEVs. The underlying mechanism remains to be illuminated.

miRs are a kind of noncoding RNA. miRs can regulate the expression of genes by binding to the 3’UTR of target mRNAs and inducing the degradation of mRNAs [50, 51]. sEVs, as a cell-to-cell communication mode, transport miRs to recipient cells, where miRs act as gene expression mediators [52]. The current study revealed that miR-486-5p from hsEVs exerted anti-apoptosis and proangiogenic effects. miR-486-5p knockdown abolished the benefit of hsEVs treatment, while miR-486-5p overexpression in nsEVs partly captured these effects. A dual luciferase reporter assay showed that miR-486-5p inhibited the luciferase activity of HEK293 cell transfected with a plasmid containing the wild-type 3’UTR of PTEN mRNA, but did not influence cells transfected with mutated sites. The results indicated that PTEN is a direct target and effector of miR-486-5p. 

A recent study found that miR-486-5p downregulated the expression of PIK3R1, hence activating the PI3K/AKT pathway in human gastric cancer cells [53]. In another study, miR-486-5p inhibited inflammation and apoptosis of nucleus pulposus cells by targeting FOXO1, which is a downstream molecule of AKT [54]. Taken together, miR-486-5p might activate the PI3K/AKT pathway by interacting with PTEN, PIK3R1 and FOXO1 in different cellular contexts, therefore protecting cells from apoptosis. The importance of the PI3K/AKT pathway of MSCs in the treatment of ischemic diseases has been emphasized by other researchers [40]. In addition, we found that miR-486-5p promotes the synthesis of angiogenesis-related cytokines, such as VEGF-A, in ADSCs. Other researchers discovered that miR-486-5p increases the level of VEGF-C in pancreatic cancer cells [55]. In a myocardial infarction model, miR-486-5p reduced the production of MMP19 in fibroblasts, reducing the cleavage of VEGF-A and therefore promoting angiogenesis and cardiac repair [56]. Our study provided compelling evidence that miR-486-5p increases the production of VEGF-A through the AKT/MTOR/HIF-1α signaling pathway. These results imply that miR-486-5p might be involved in the regulation of physiological and pathological angiogenesis.

In this study, we first demonstrated that hsEVs promoted the proliferation of ADSCs and reduced the apoptosis caused by H_2_O_2_. Moreover, hsEVs treatment inhibited the expression of PTEN in ADSCs and caused AKT activation and HIF-1α accumulation. Additionally, our study suggests that both nsEVs and hsEVs improve the proangiogenic ability of ADSCs. Furthermore, we identified that miR-486-5p is upregulated in hsEVs compared to nsEVs and that miR-486-5p drives the effects of nsEVs by targeting PTEN. IN in vitro experiments, we used H_2_O_2_ to mimic the hostile microenvironment that ADSCs that encounter after transplantation. However, a series of detrimental factors lead to poor cell retention posttransplantation, including anoikis, ischemia, hypoxia and inflammation. H_2_O_2_ might not fully capture the complicated environment. However, evidence from the in vivo model showed that hsEVs-ADSCs still showed better survival in ischemic muscles and exhibited better proangiogenic effects. Therefore, we believe that hsEVs could work as a therapeutic booster of ADSCs in the treatment of CLI, even in other ischemic diseases.

Although our findings indicate that hsEVs increased the therapeutic efficacy in limb ischemia model, several challenges are non-negligible to translate such protocol into clinic. The culture conditions of HUVECs must be standardized, and the batch reproducibility has to be well defined. Also, differential centrifugation, as a conventional method to enrich EVs, is limited by the rotor sizes for clinical use. Therefore, other techniques, including size-exclusion liquid chromatography or ultrafiltration-based methods, may be more practical for the large-scale production of EVs. Since the functions and cargos of sEVs are susceptible to external disturbance, as a pharmaceutical, each batch of EVs needs to be tested in functional assays for quality control.

## Conclusion

Because the local ischemic environment leads to the exhaustion of injected ADSCs, we prepared hsEVs from HUVECs to reduce cell death and increase the proangiogenic ability of ADSCs in vitro and in vivo, possibly through miR-486-5p mediated PTEN repression. Taken together, hsEVs from HUVECs could be used as a new approach to enhance the therapeutic efficacy of ADSCs in the treatment of CLI.

## Supplementary Information

**Additional file 1**: Primers used in the qRT–PCR assay.

**Additional file 2: Fig. S1**. Identification of ADSCs (A)The morphology of ADSCs was observed under an optical microscope. (B) FCM was used to detect the surface markers of ADSCs, including CD34, CD45, CD44, CD90, CD29 and CD105.

**Additional file 3: Fig. S2**. hsEVs induced HIF-1α accumulation in ADSCs. (A) The level of HIF-1α mRNA was detected by qRT–PCR. (B) The inhibitory effect of PX478 was tested by western blotting. All data are representative of three independent experiments and are shown as the mean ± SEM. (n = 3; *P < 0.05; **P < 0.01).

**Additional file 4: Fig. S3**. Verification of miR-486-5p overexpression and knockdown. (A) The level of miR-486-5p in different sEVs was measured by qRT–PCR. (B) ADSCs were treated with different sEVs for 12 h, and the level of miR-486-5p in ADSCs was detected by qRT–PCR. Relative gene expression was normalized to U6, and data were analyzed via the 2^−ΔΔCt^ method. All data are representative of three independent experiments and are shown as the mean ± SEM. (n = 3; *P < 0.05; **P < 0.01).

**Additional file 5: Fig. S4**. Scratch assay of HUVECs treated with different CMs. (A) Images of HUVECs after scratching and treatment with CM for 0 h.

**Additional file 6: Fig. S5**. The transfection of dual-reporter genes (A) The transfection of the Fluc/eGFP gene was observed under a fluorescence microscope.

**Additional file 7**: The raw images of western blotting.

## Data Availability

The datasets generated/analyzed during the current study are available.

## Abbreviations

ADSC: Adipose derived mesenchymal stem cell
MSC: Mesenchymal stem cell
sEVs: Small extracellular vesicles
HUVEC: Human umbilical vein endothelial cell
CLI: Critical limb ischemia
ROS: Reactive oxygen species
CM: Conditioned media
GC: Gastrocnemius
BLI: Bioluminescence imaging
LDPI: Laser Doppler perfusion imager
HIF-1α: Hypoxia inducible factor 1 subunit alpha
PTEN: Phosphatase and tensin homolog
