# Supplementary material for: Small extracellular vesicles of hypoxic endothelial cells regulate the therapeutic potential of adipose-derived mesenchymal stem cells via miR-486-5p/PTEN in a limb ischemia model
Source: J Nanobiotechnology. 2022 Sep 24;20:422. doi: 10.1186/s12951-022-01632-1 (PMC9509557; doi:10.1186/s12951-022-01632-1)
Supplement: Supplementary file 1 — Additional file 1: Primers used in the qRT–PCR assay. [file 12951_2022_1632_MOESM1_ESM.docx]

| **Gene** | **Primer sequence 5’-3’** |
| --- | --- |
| *PTEN* forward | TTTGAAGACCATAACCCACCAC |
| *PTEN* reverse | ATTACACCAGTTCGTCCCTTTC |
| *HIF-1α* forward | CGTTCCTTCGATCAGTTGTC |
| *HIF-1α* reverse | TCAGTGGTGGCAGTGGTAGT |
| *VEGFA* forward | CGAAGTGGTGAAGTTCATGGATG |
| *VEGFA* reverse | TTCTGTTCAGTCTTTCCTGGTGAG |
| *ANG1* forward | GGTGTTTTACTAAAGGGAGGAA |
| *ANG1* reverse | TTGCAAAACACCTTTTTGGG |
| *ANG2* forward | CAGAGGCTGCAAGTGCTGGAGAACA |
| *ANG2* reverse | GAGGGAGTGTTCCAAGAGCTGAAGT |
| *HGF* forward | GGACAAAGGAAAAGAAG |
| *HGF* reverse | GATTGCTTGTGAAACACC |
| *ACTB* forward | CATGTACGTTGCTATCCAGGC |
| *ACTB* reverse | CTCCTTAATGTCACGCACGAT |
| miR-26a-5p | GCAGTTCAAGTAATCCAGGATAG |
| miR-23b-3p | AGATCACATTGCCAGGGA |
| miR-486-5p | TACTGAGCTGCCCCGAGAAA |
| miR-494-3p | TGAAACATACACGGGAAACCTC |
| miR-92a-3p | ACTTGTCCCGGCCTGTAAA |
| miR-188-5p | CATCCCTTGCATGGTGGA |
| miR-25-3p | CATTGCACTTGTCTCGGTCT |
| miR-6866-3p | GCAGATCCCTTTATCTGTCCT |
| miR-338-3p | GAGTCCAGCATCAGTGATTTTG |
| miR-320d | AAAAGCTGGGTTGAGAGGA |
| miR-320c | AAAAGCTGGGTTGAGAGGGT |
| miR-152-3p | TCAGTGCATGACAGAACTTGG |
| miR-148b-3p | GTCAGTGCATCACAGAACTTTGT |
| miR-141-3p | GTAACACTGTCTGGTAAAGATGG |
| *U6* forward | CTCGCTTCGGCAGCACA |
| *U6* reverse | AACGCTTCACGAATTTGCGT |

**Supplementary Table 1. Primers used in the qPCR assay**
