# Supplementary figures and images for: Small extracellular vesicles of hypoxic endothelial cells regulate the therapeutic potential of adipose-derived mesenchymal stem cells via miR-486-5p/PTEN in a limb ischemia model
Source: J Nanobiotechnology. 2022 Sep 24;20:422. doi: 10.1186/s12951-022-01632-1 (PMC9509557; doi:10.1186/s12951-022-01632-1)

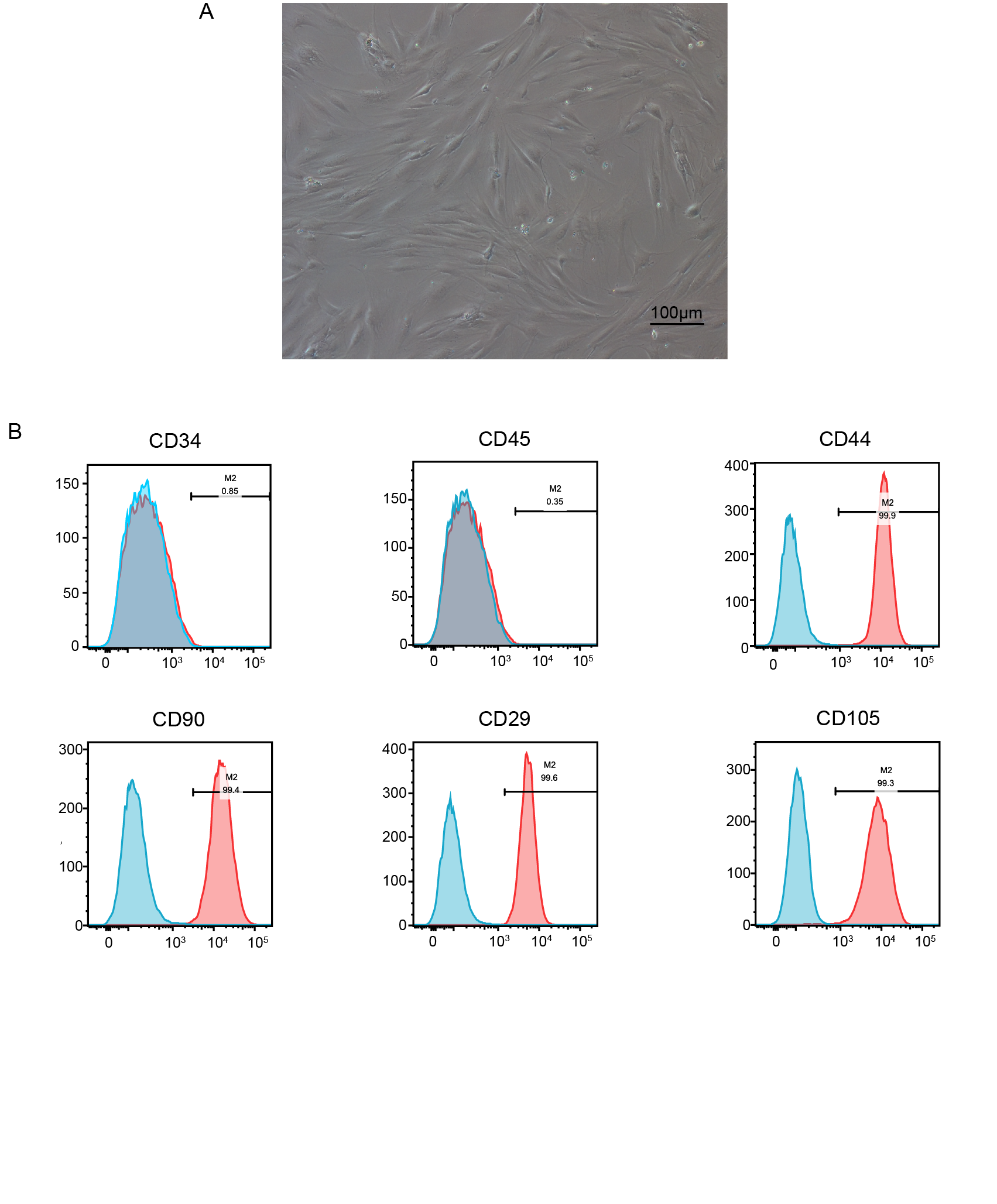

Supplement: Supplementary file 2 — Additional file 2: Fig. S1. Identification of ADSCs (A)The morphology of ADSCs was observed under an optical microscope. (B) FCM was used to detect the surface markers of ADSCs, including CD34, CD45, CD44, CD90, CD29 and CD105. [file 12951_2022_1632_MOESM2_ESM.tif]

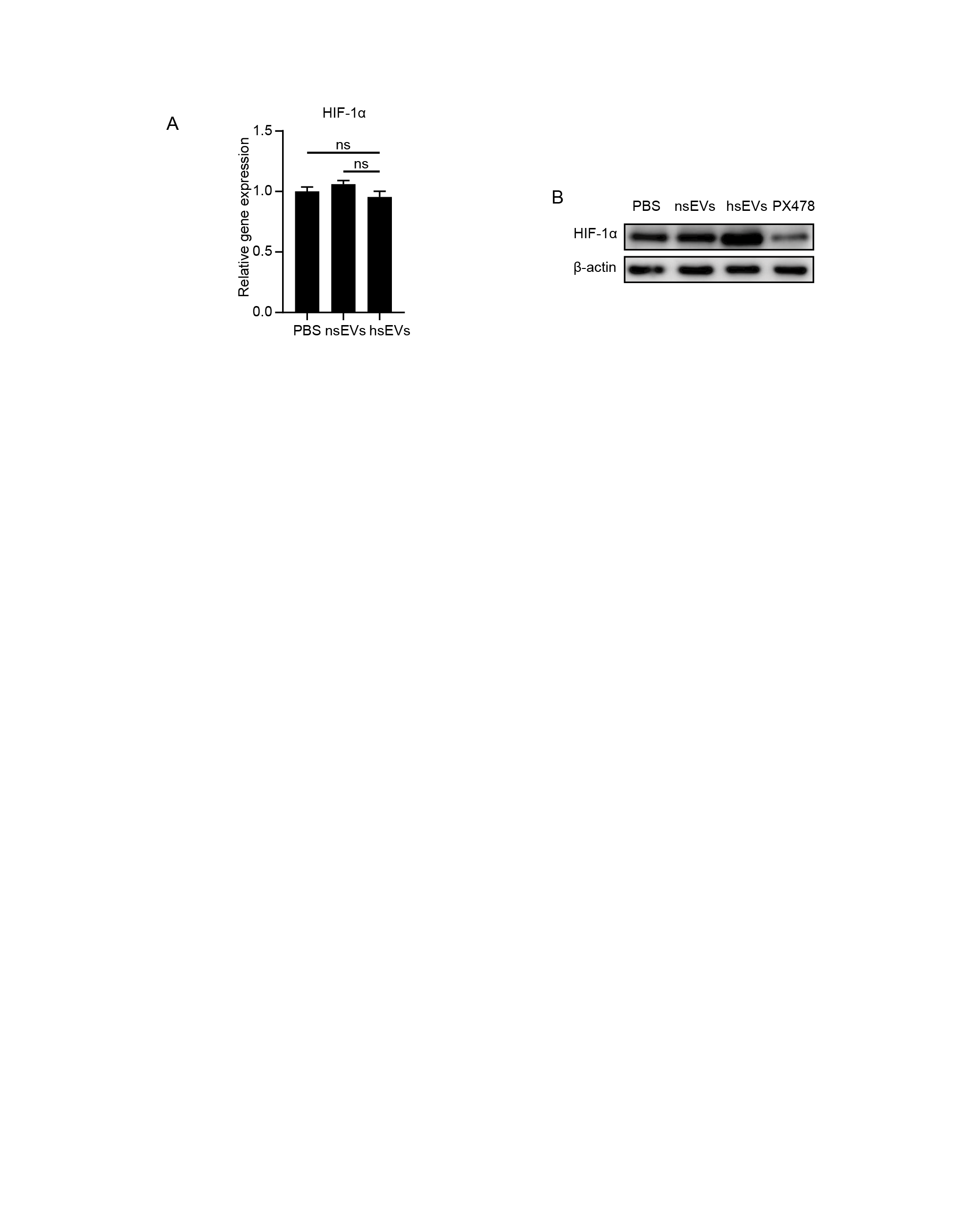

Supplement: Supplementary file 3 — Additional file 3: Fig. S2. hsEVs induced HIF-1α accumulation in ADSCs. (A) The level of HIF-1α mRNA was detected by qRT–PCR. (B) The inhibitory effect of PX478 was tested by western blotting. All data are representative of three independent experiments and are shown as the mean ± SEM. (n = 3; *P < 0.05; **P < 0.01). [file 12951_2022_1632_MOESM3_ESM.tif]

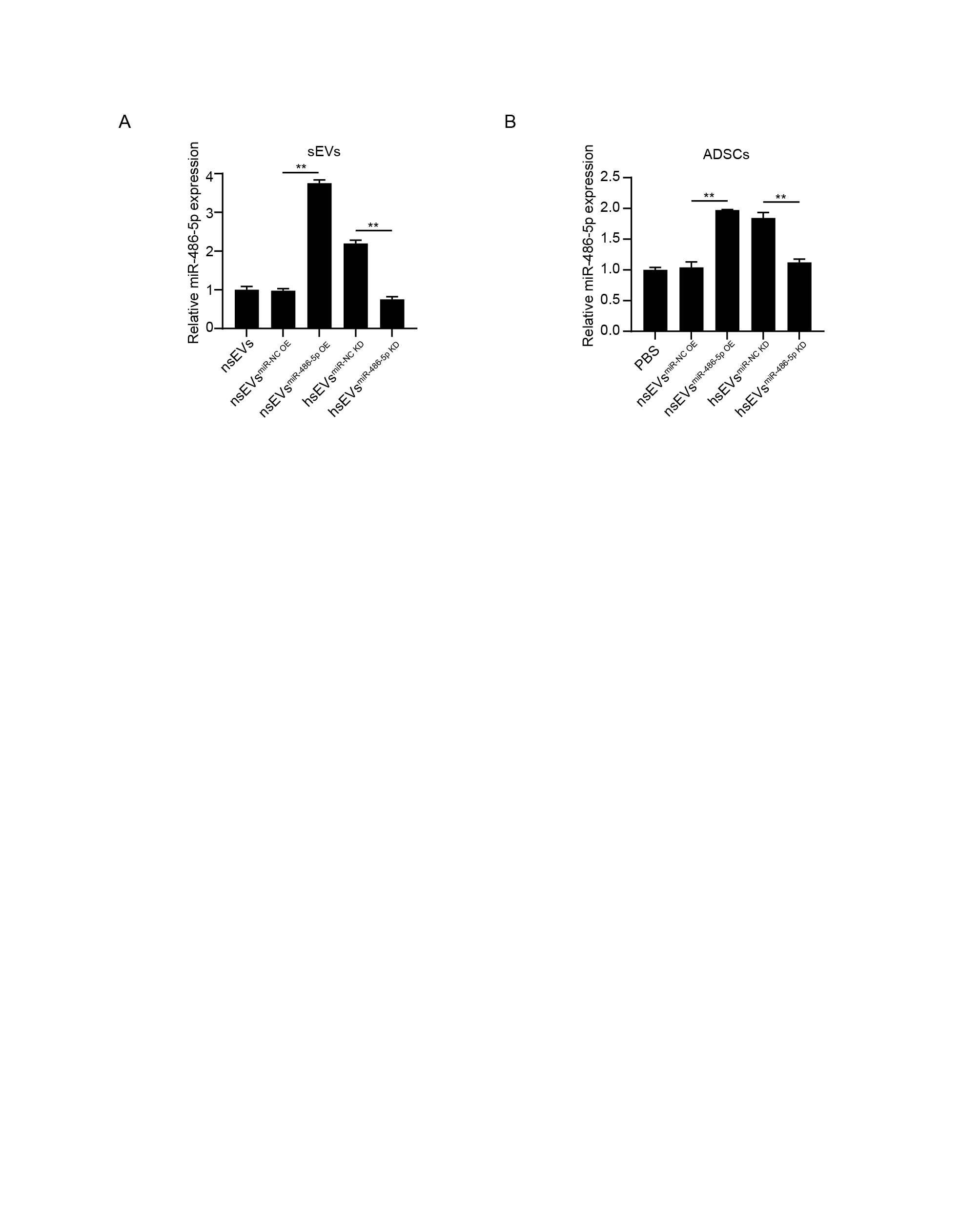

Supplement: Supplementary file 4 — Additional file 4: Fig. S3. Verification of miR-486-5p overexpression and knockdown. (A) The level of miR-486-5p in different sEVs was measured by qRT–PCR. (B) ADSCs were treated with different sEVs for 12 h, and the level of miR-486-5p in ADSCs was detected by qRT–PCR. Relative gene expression was normalized to U6, and data were analyzed via the 2−ΔΔCt method. All data are representative of three independent experiments and are shown as the mean ± SEM. (n = 3; *P < 0.05; **P < 0.01). [file 12951_2022_1632_MOESM4_ESM.tif]

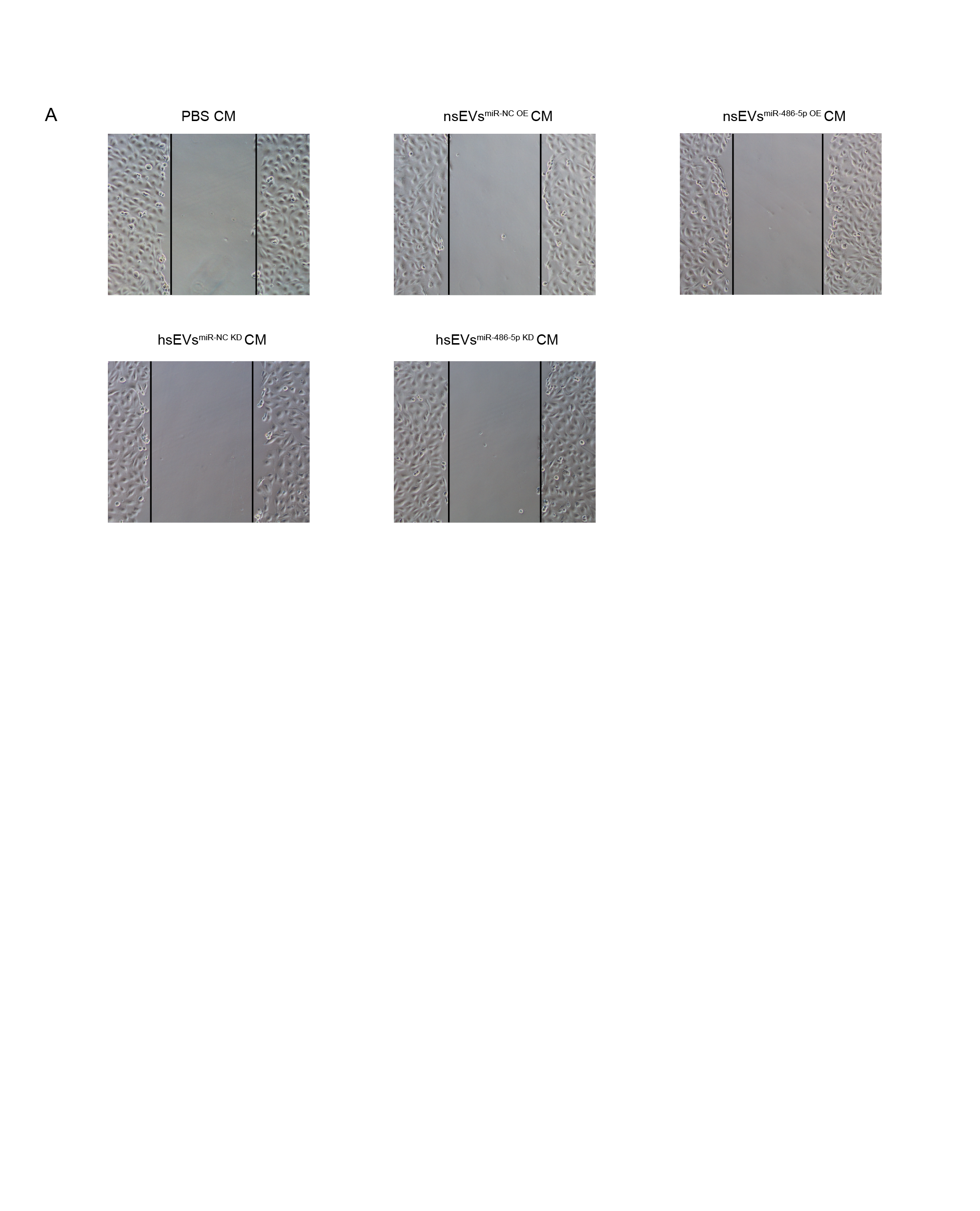

Supplement: Supplementary file 5 — Additional file 5: Fig. S4. Scratch assay of HUVECs treated with different CMs. (A) Images of HUVECs after scratching and treatment with CM for 0 h. [file 12951_2022_1632_MOESM5_ESM.tif]

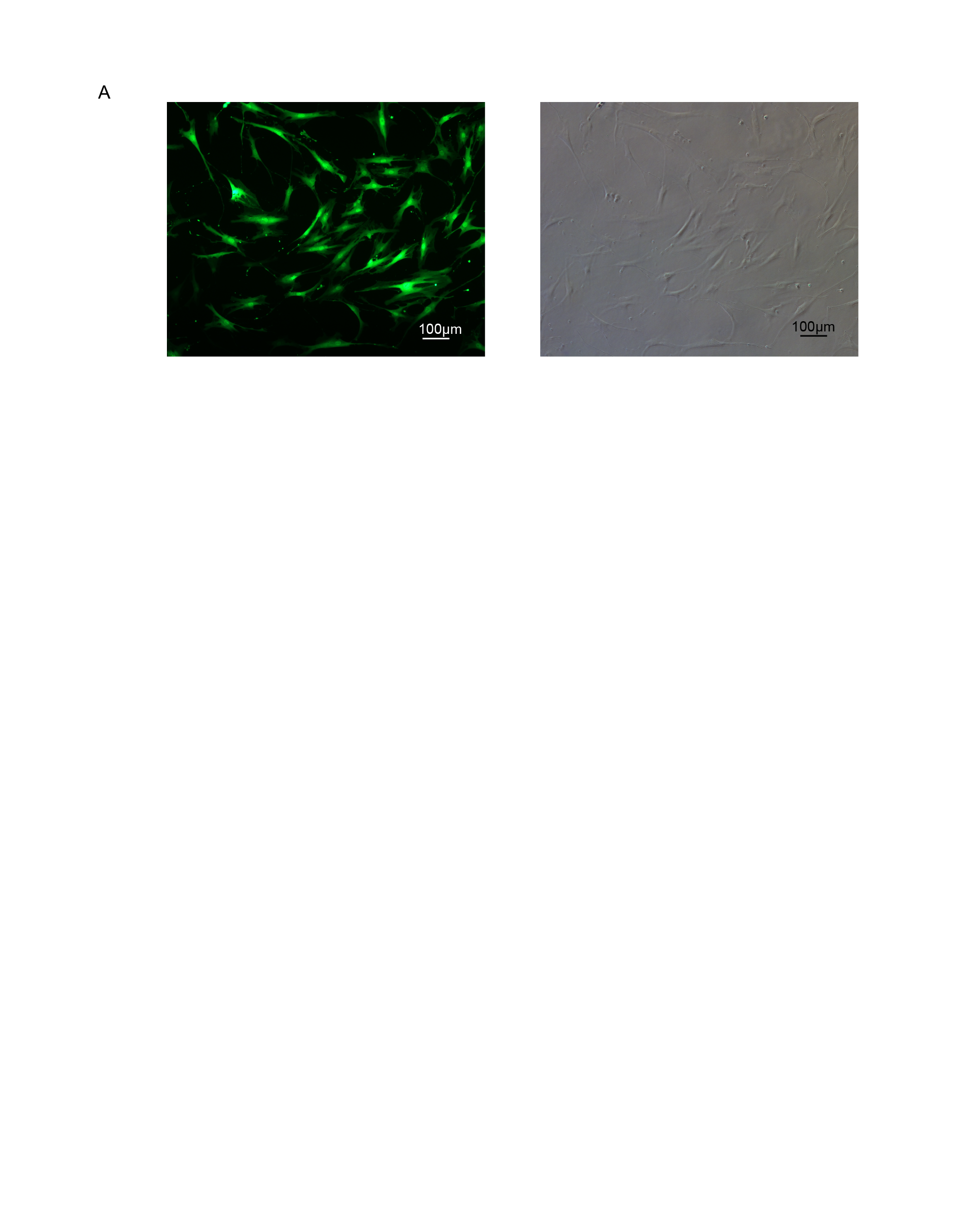

Supplement: Supplementary file 6 — Additional file 6: Fig. S5. The transfection of dual-reporter genes (A) The transfection of the Fluc/eGFP gene was observed under a fluorescence microscope. [file 12951_2022_1632_MOESM6_ESM.tif]
