# Supplementary material for: Small extracellular vesicles of hypoxic endothelial cells regulate the therapeutic potential of adipose-derived mesenchymal stem cells via miR-486-5p/PTEN in a limb ischemia model
Source: J Nanobiotechnology. 2022 Sep 24;20:422. doi: 10.1186/s12951-022-01632-1 (PMC9509557; doi:10.1186/s12951-022-01632-1)

The images of western blotting in Fig 1C.


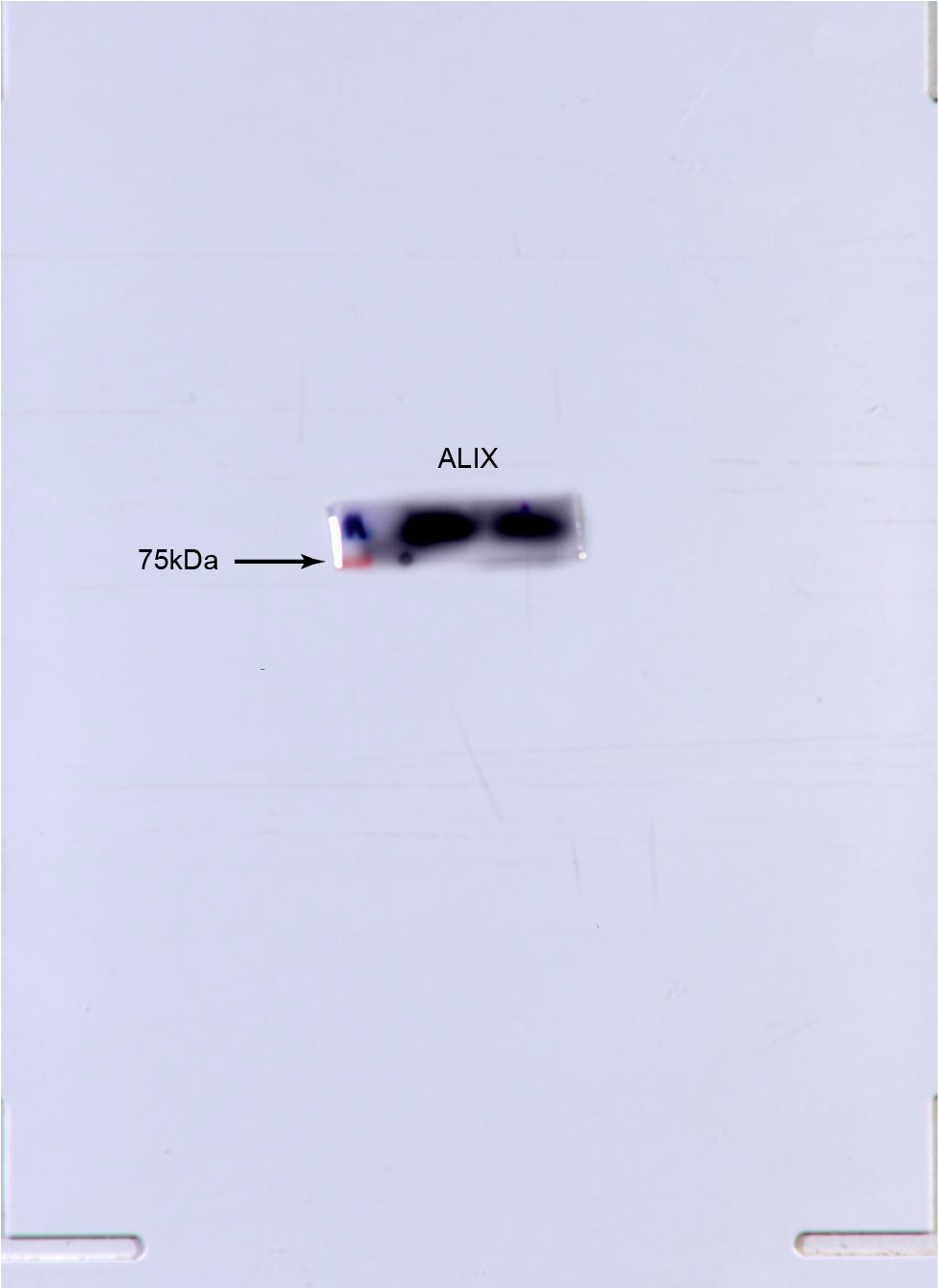

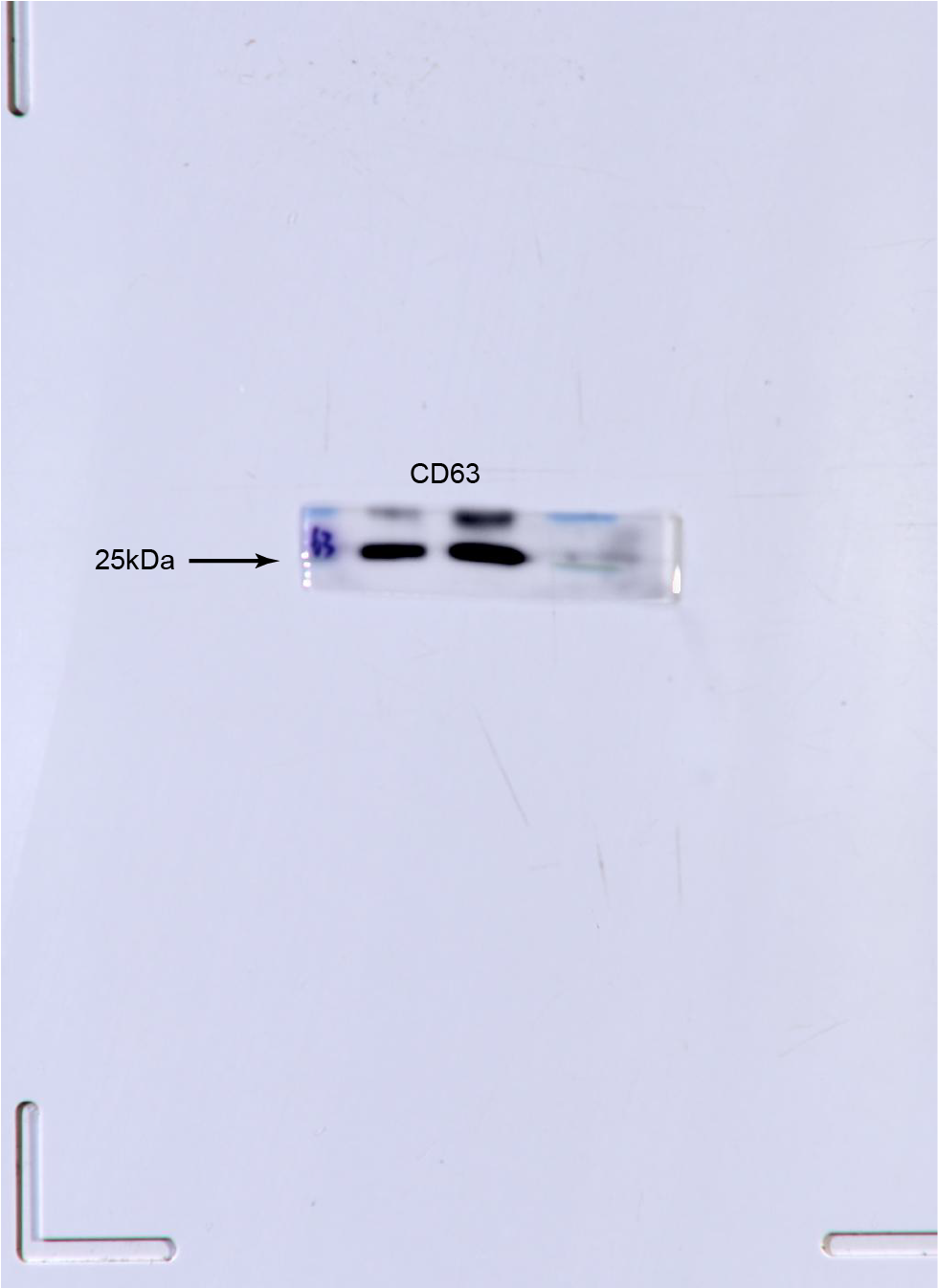

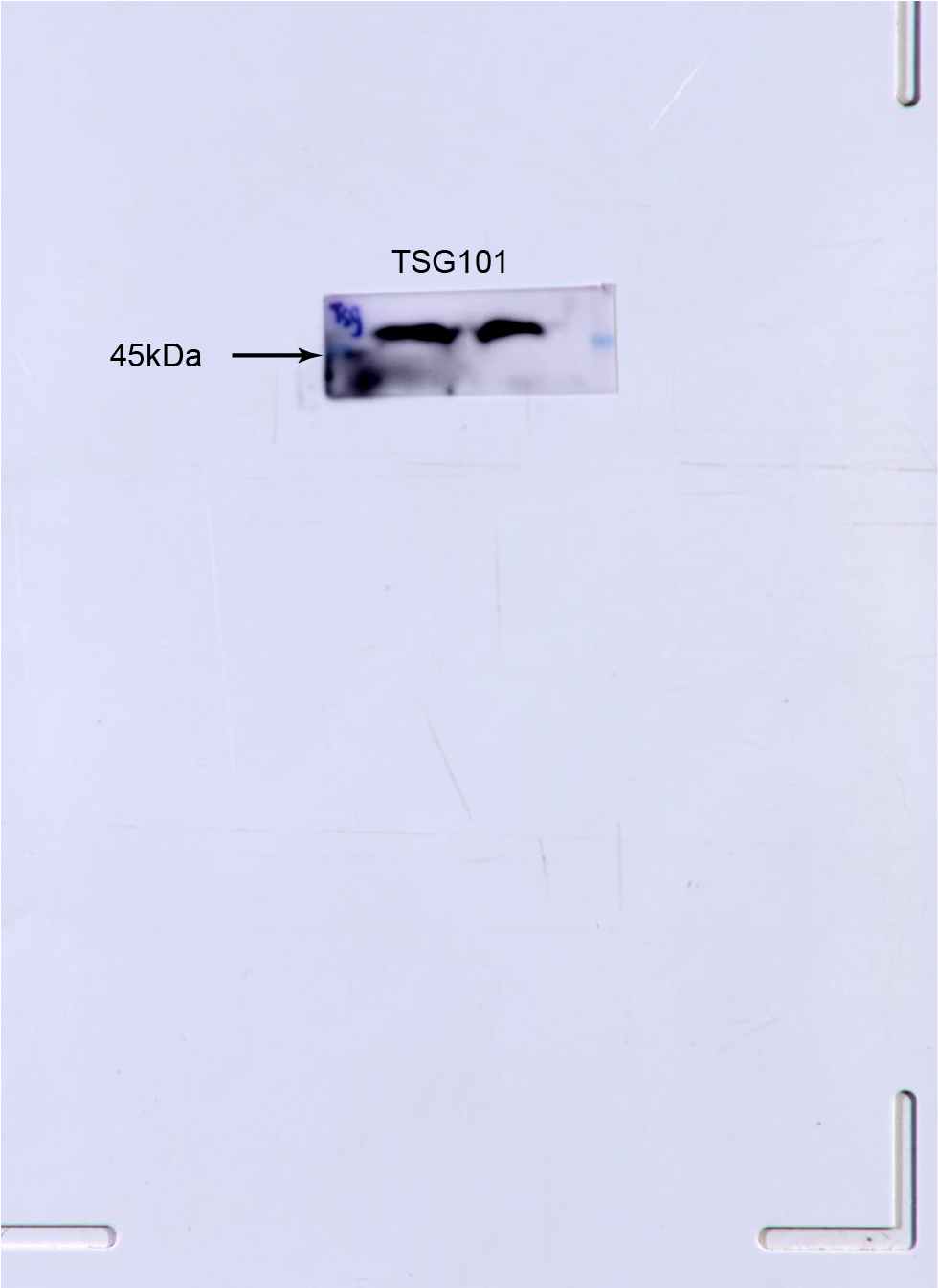

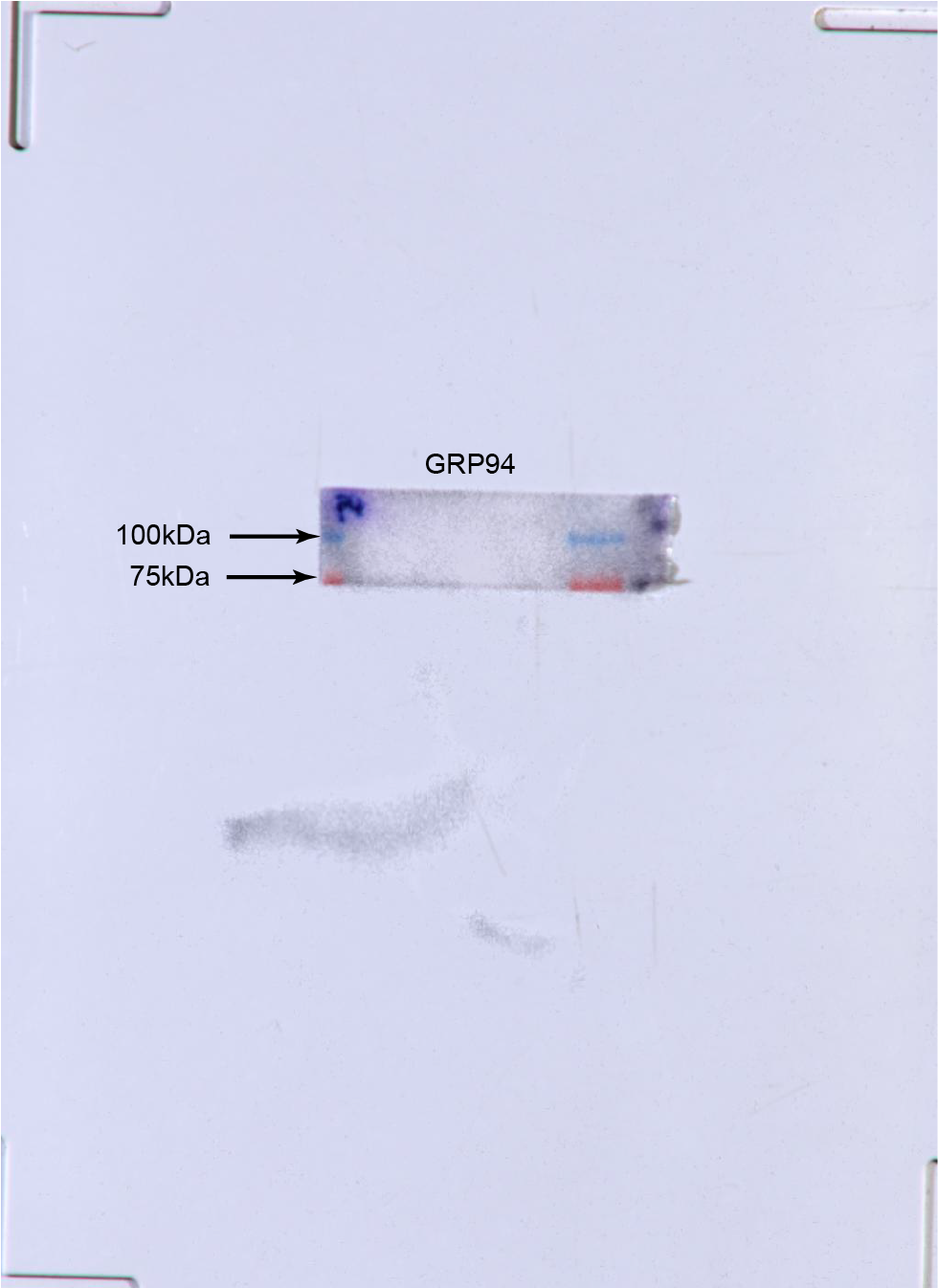


The images of western blotting in Fig 2D. Boxes indicate cropped regions.

.


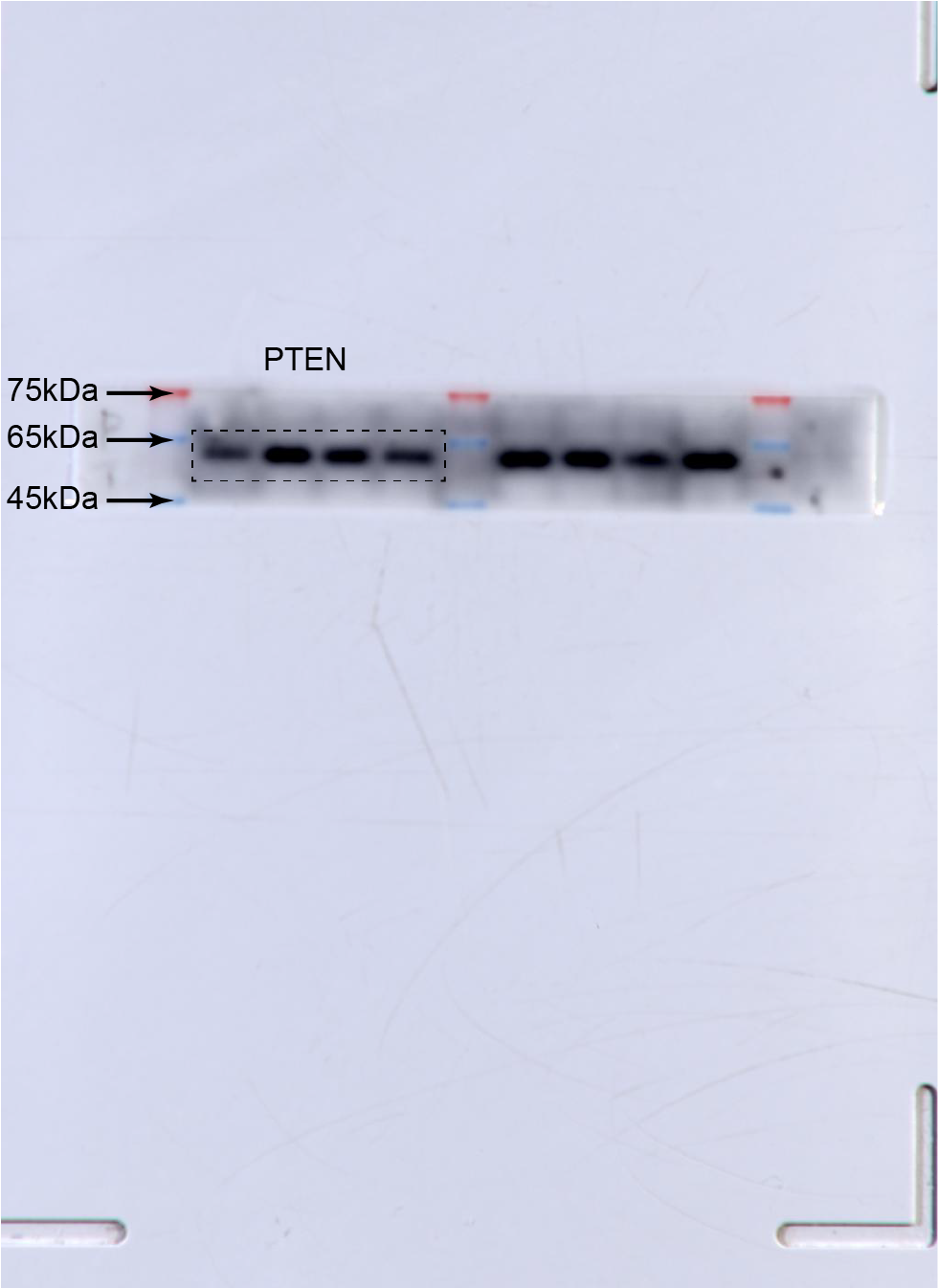

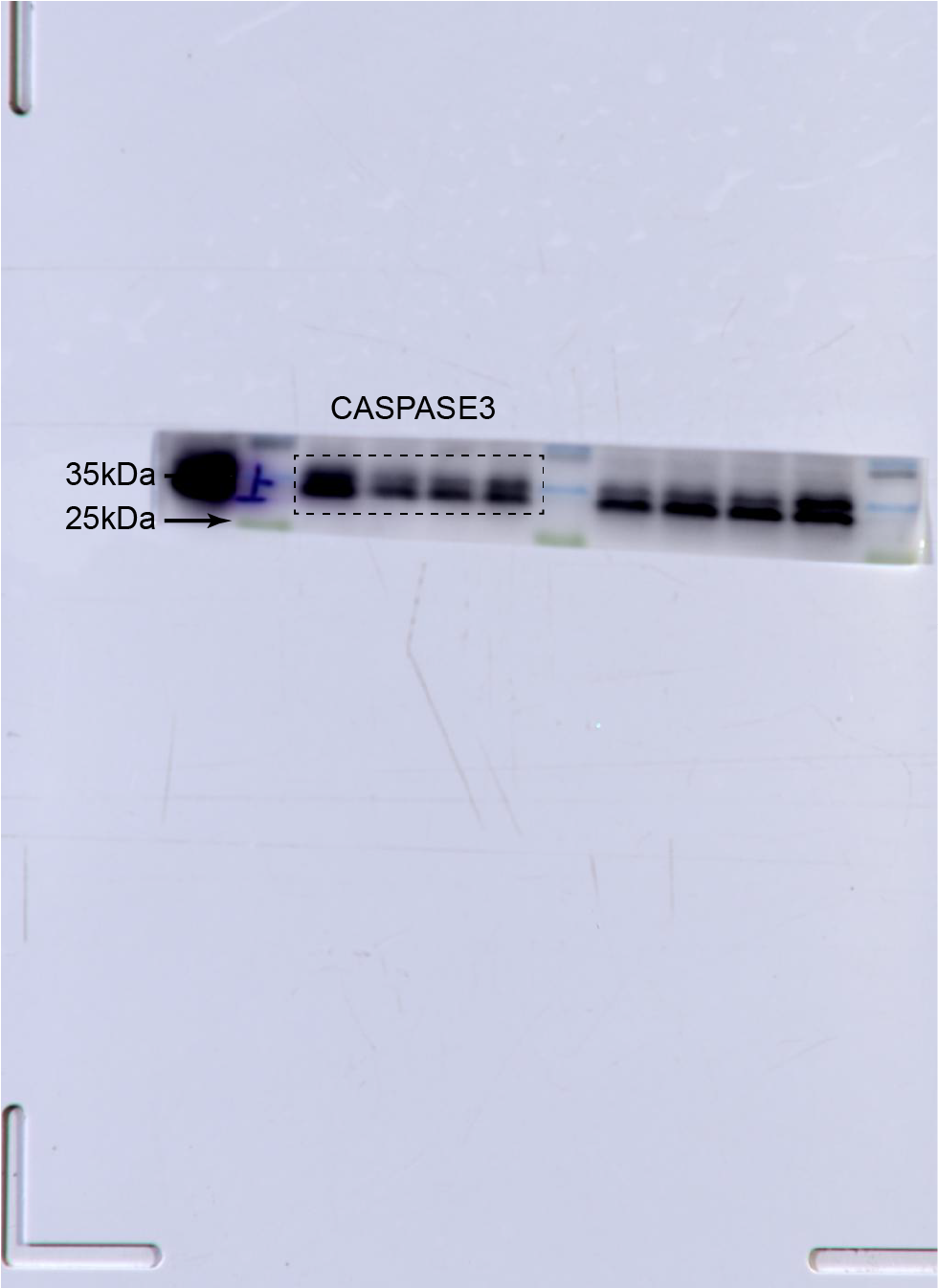

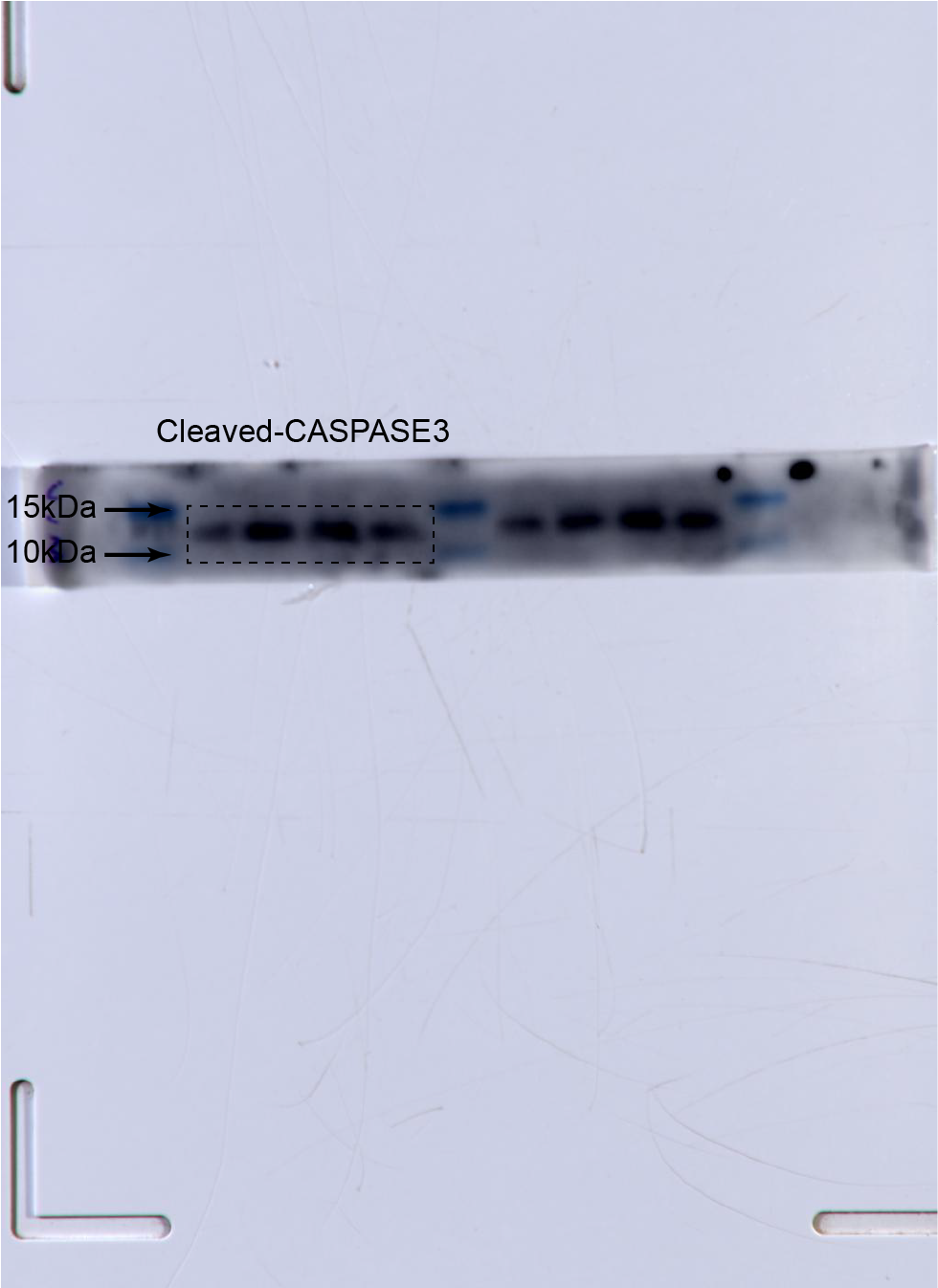

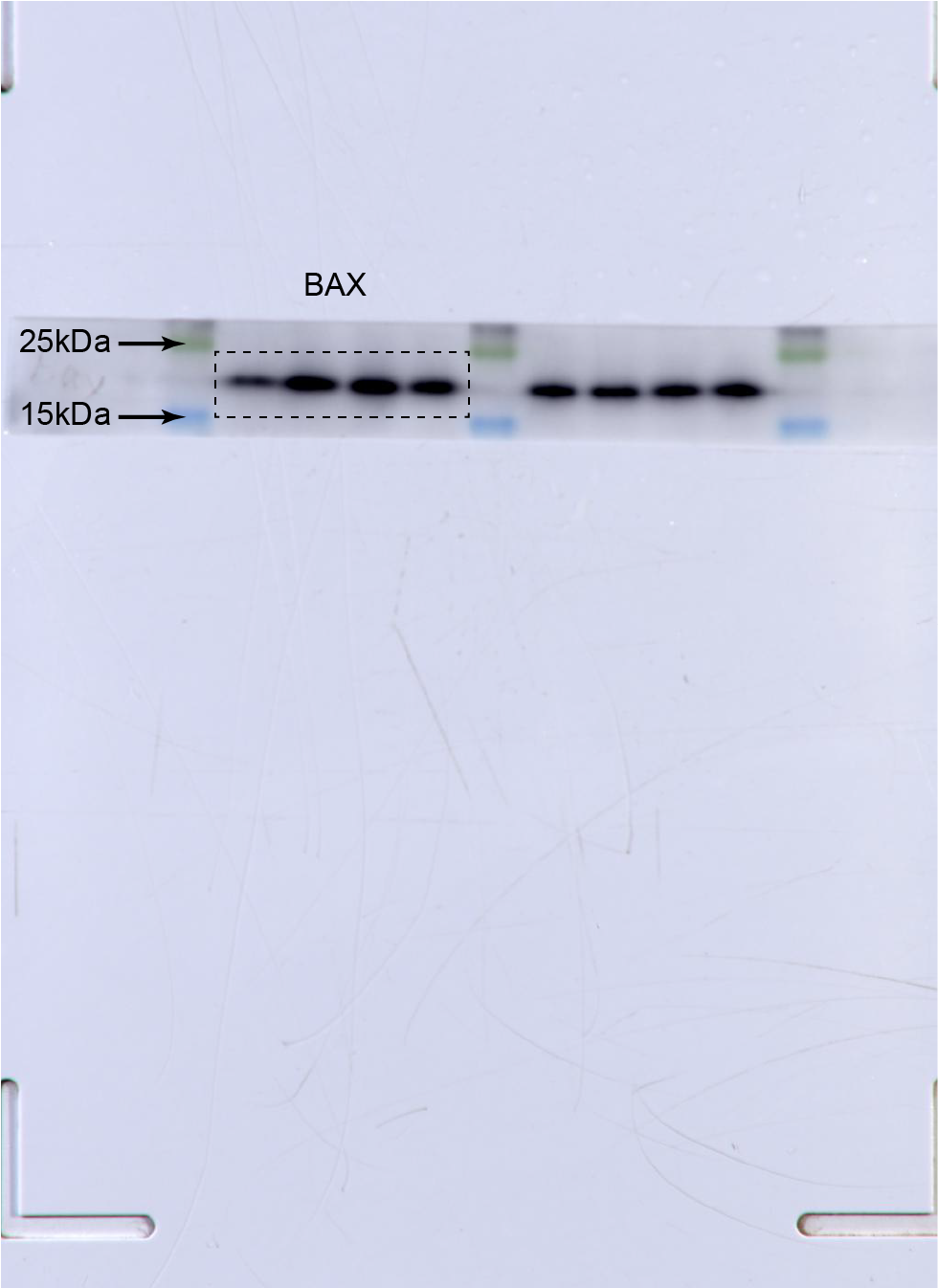

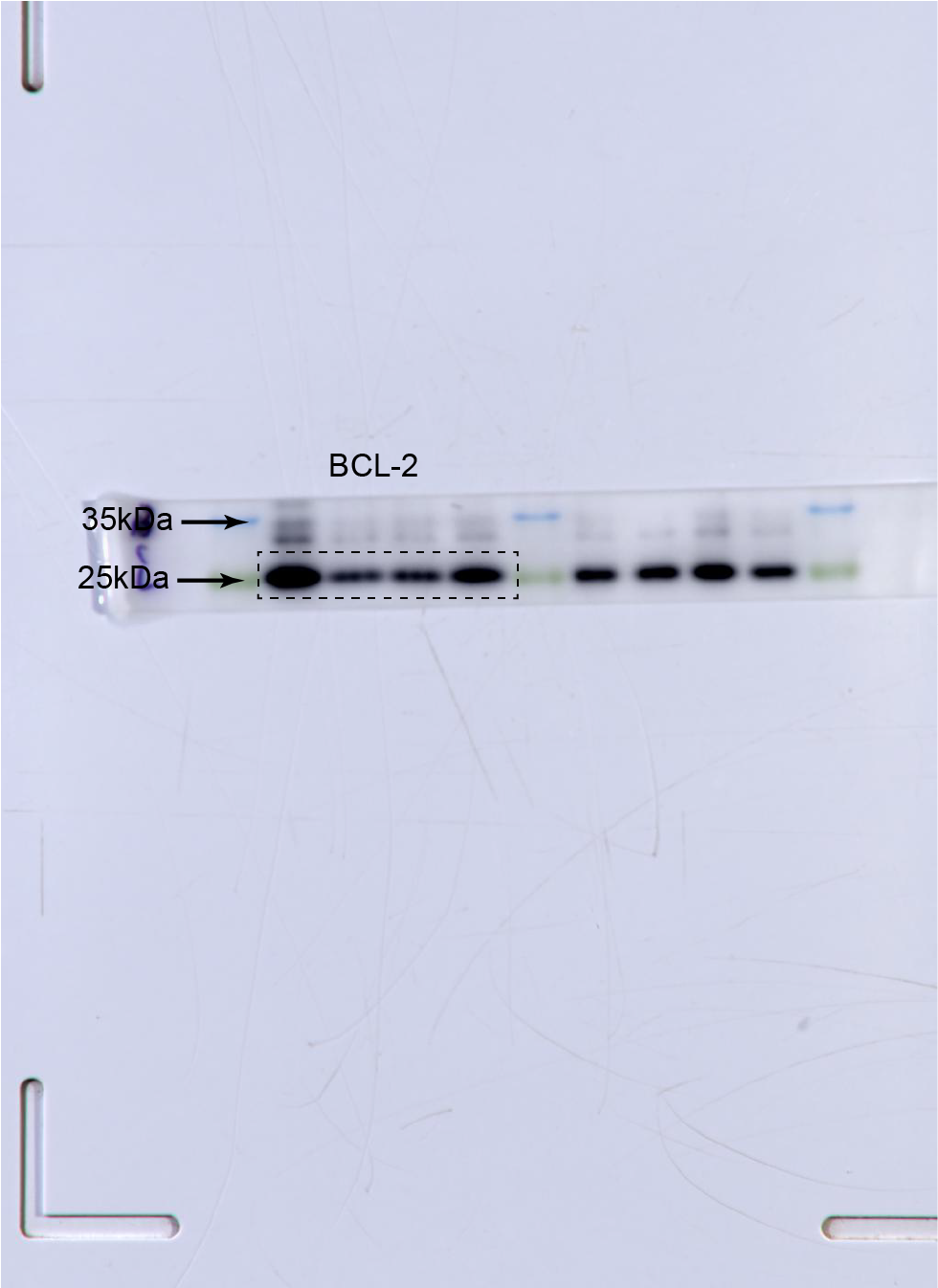

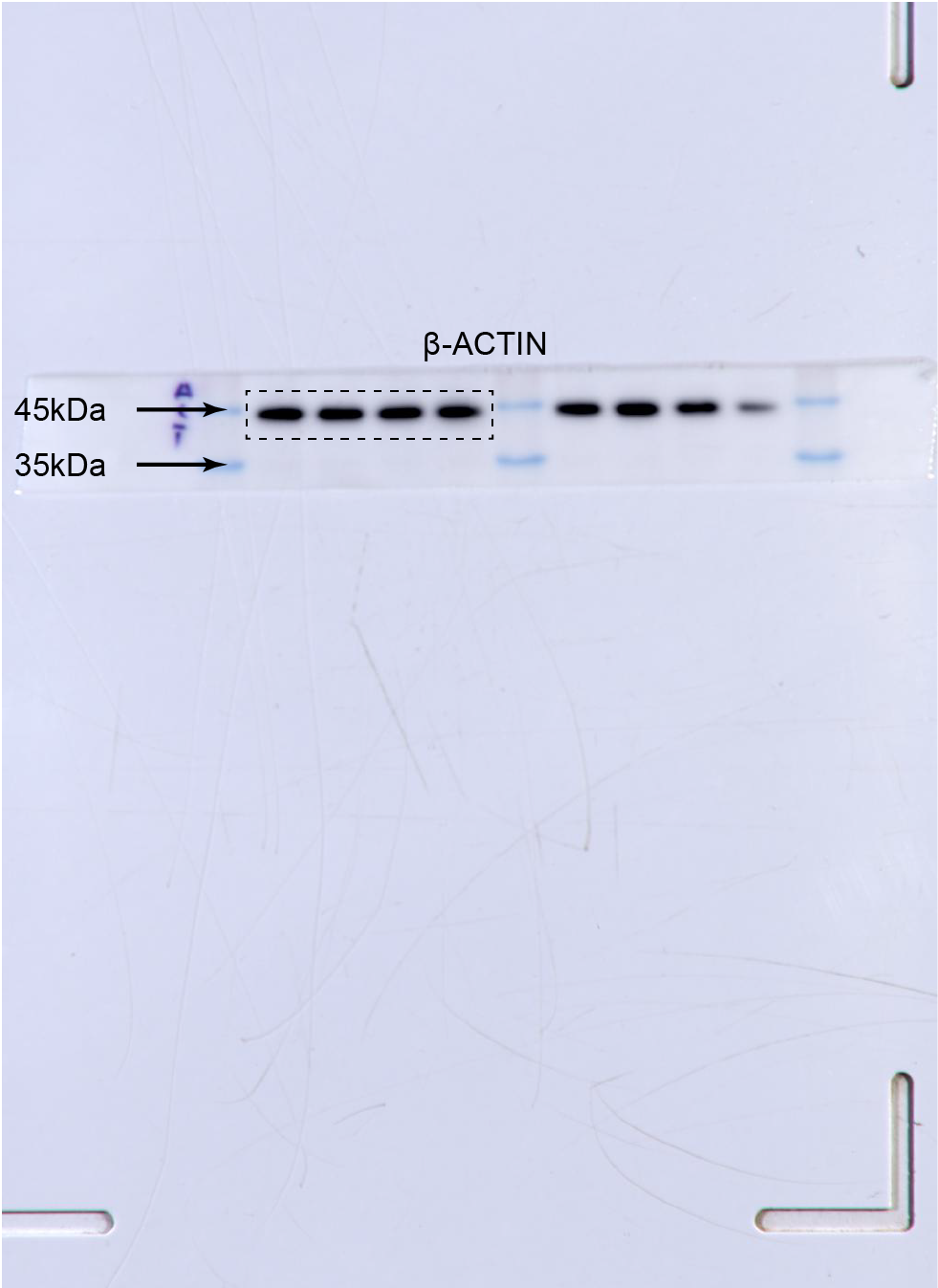


The images of western blotting in Fig 2E. Boxes indicate cropped regions.


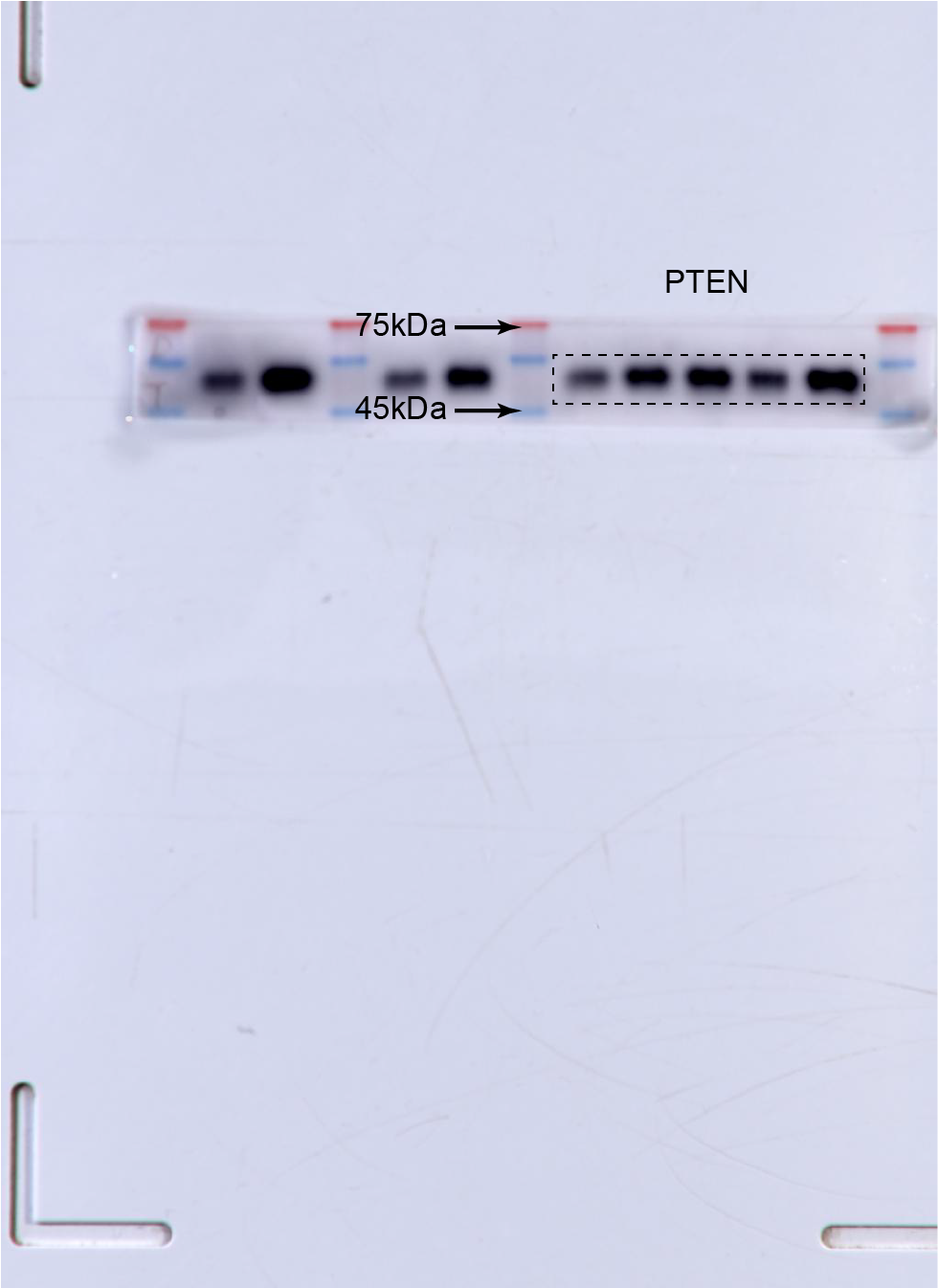

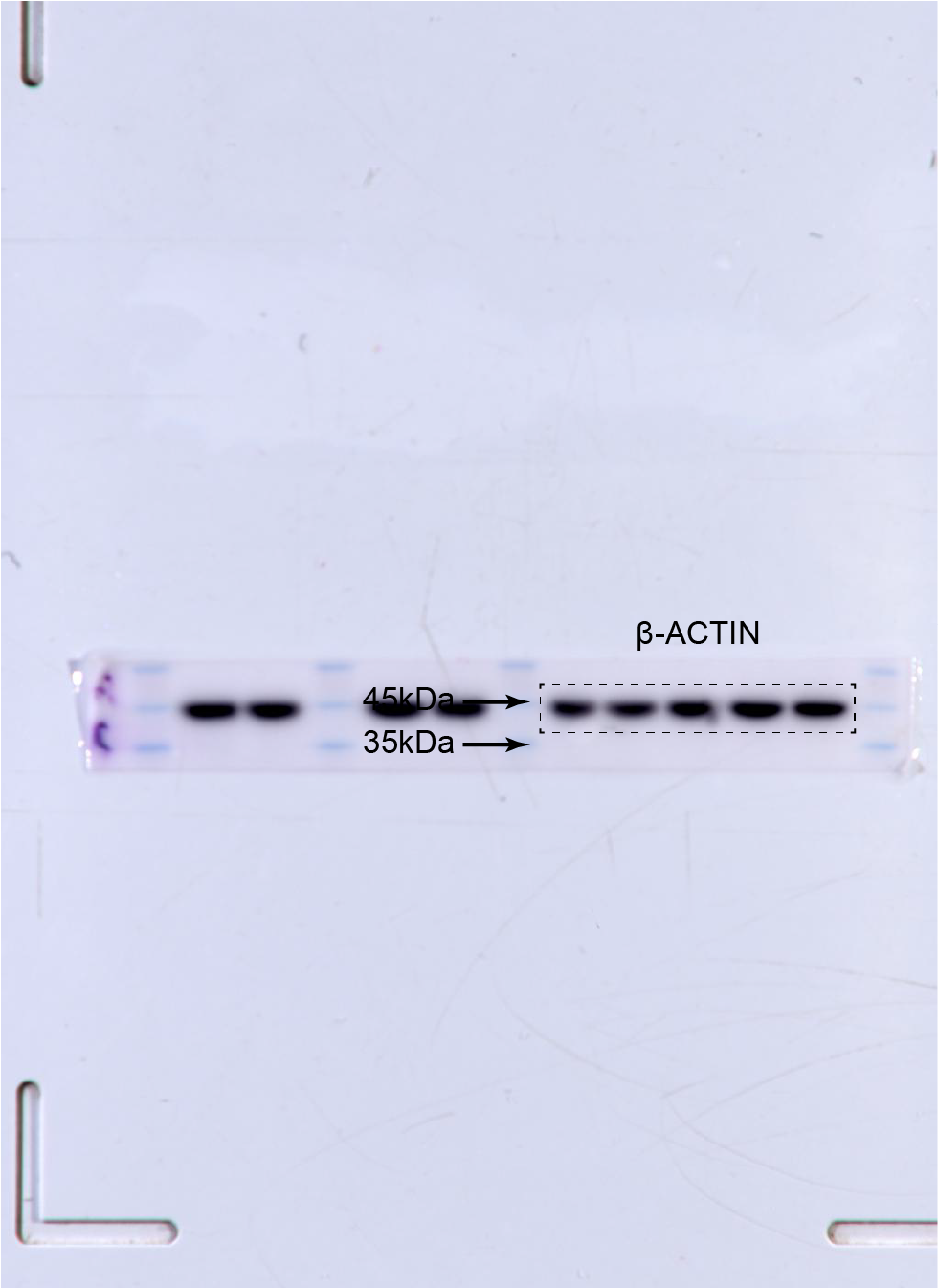


The images of western blotting in Fig 3A.


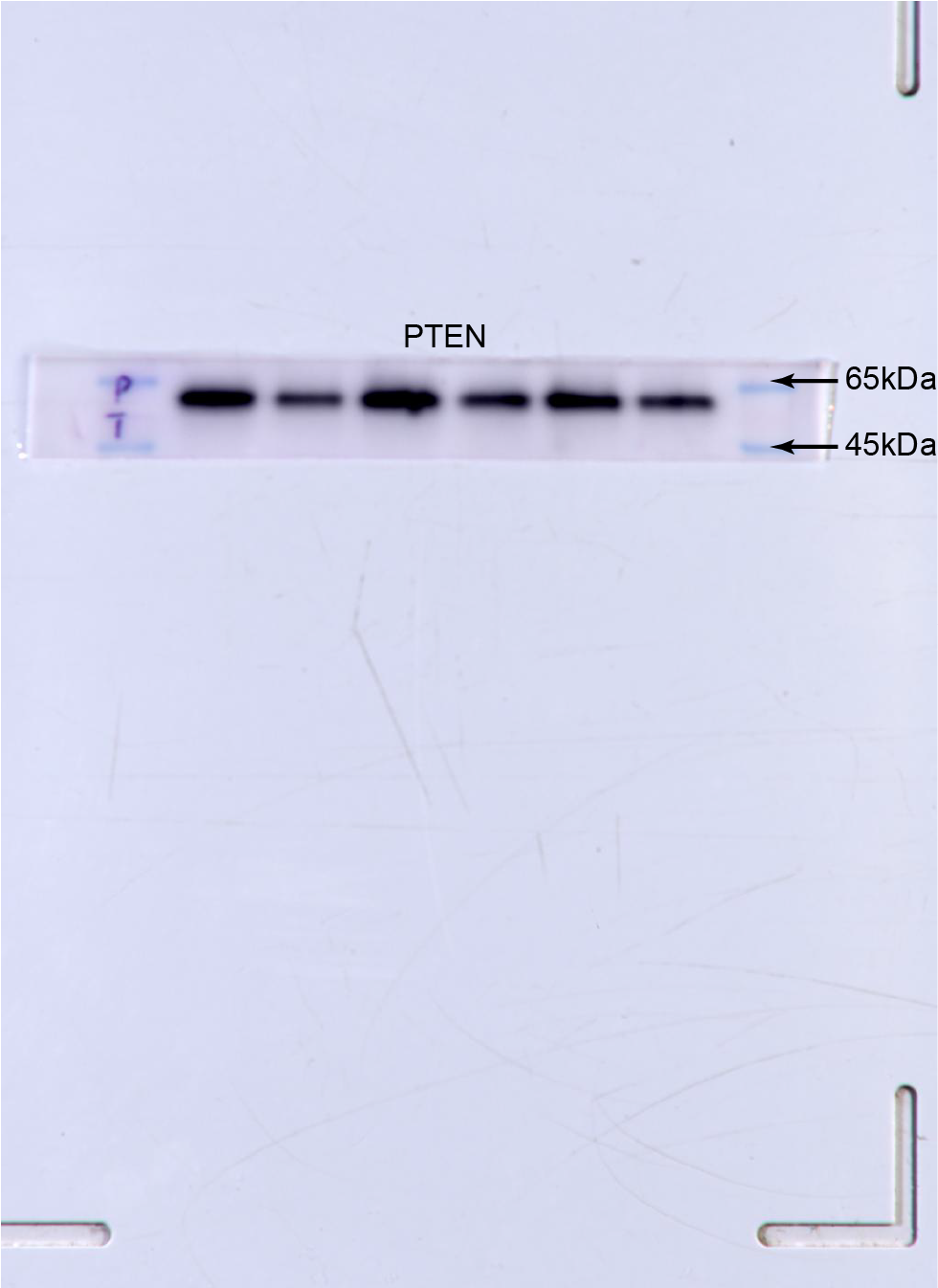

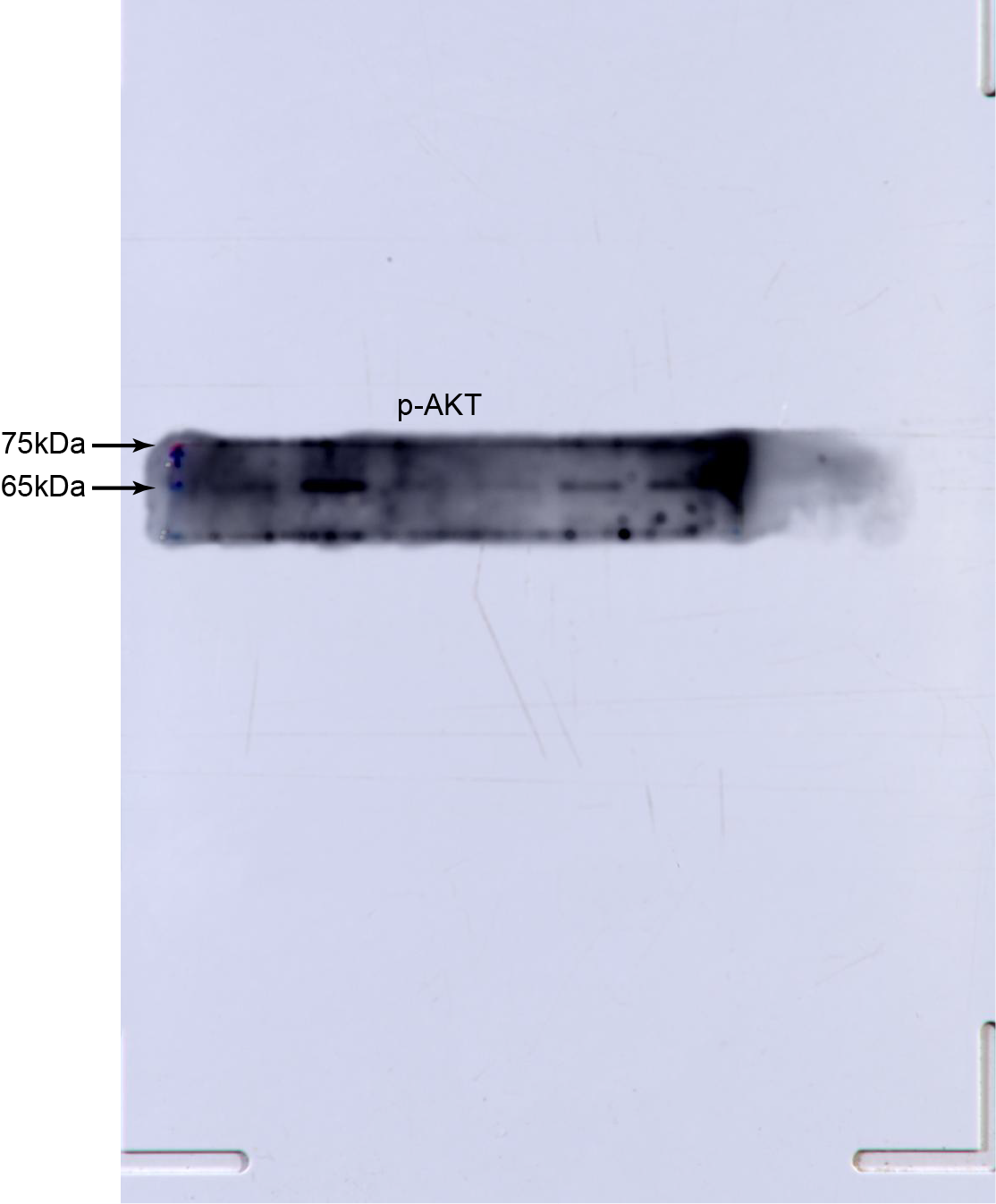

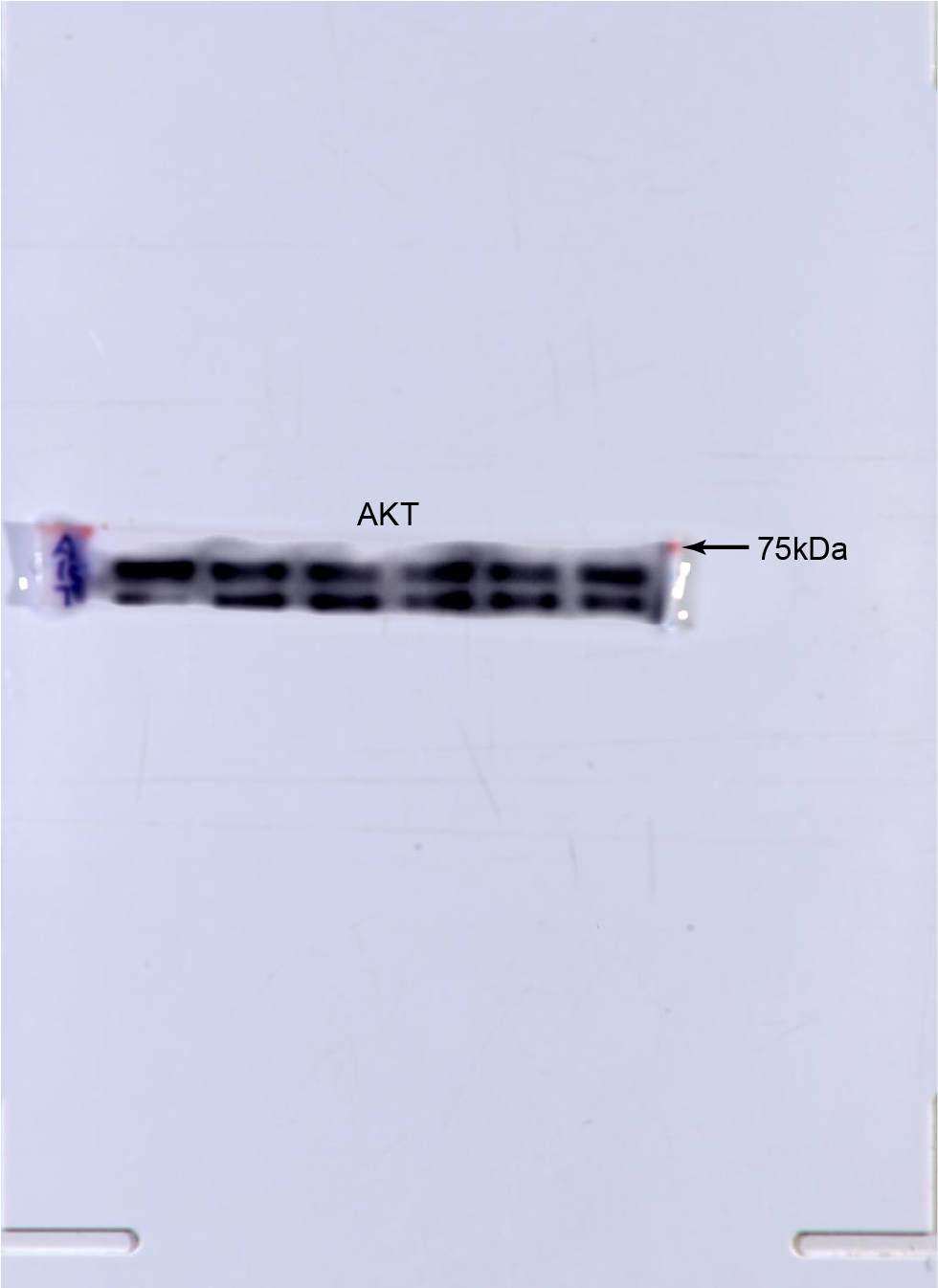

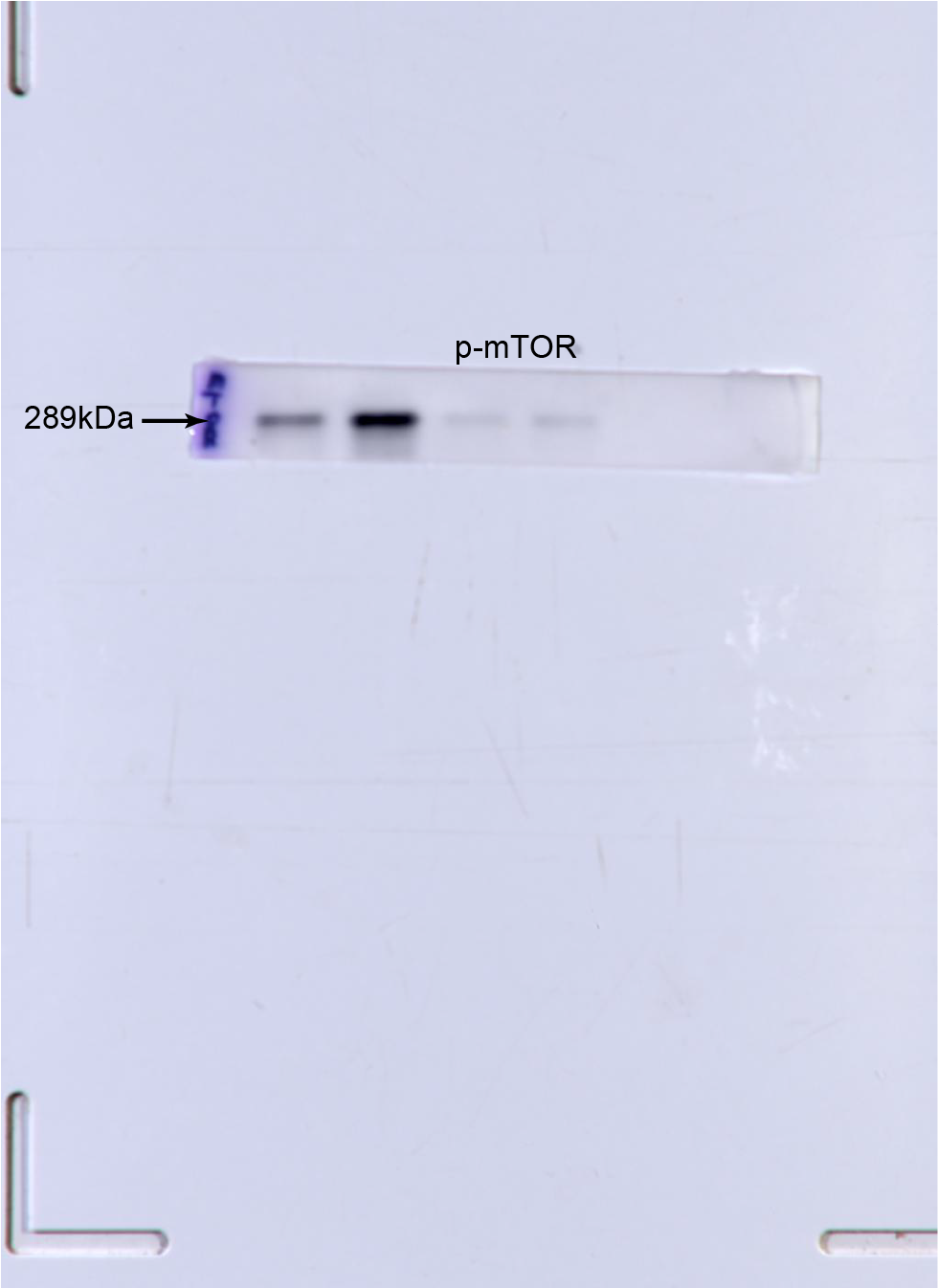

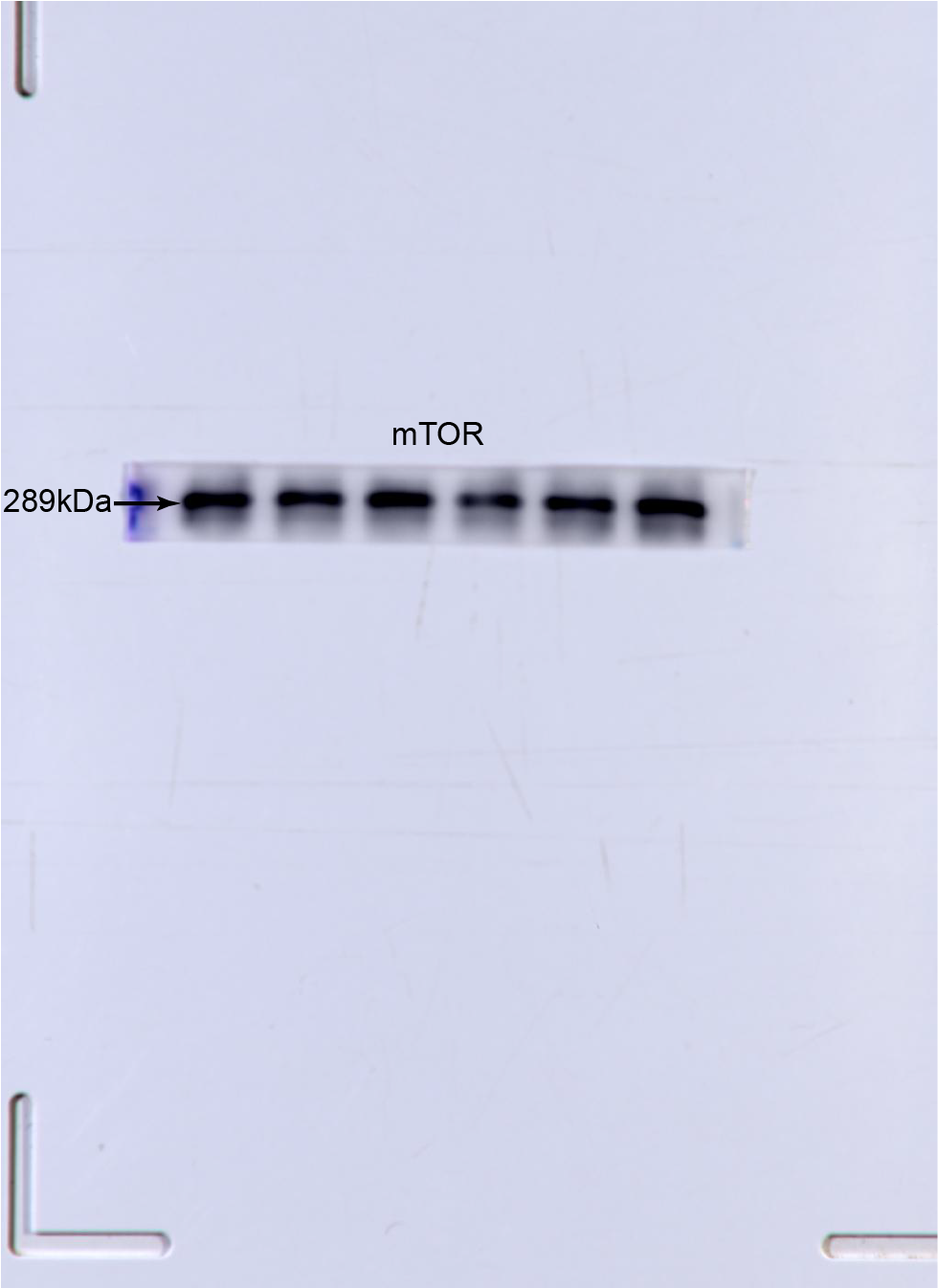

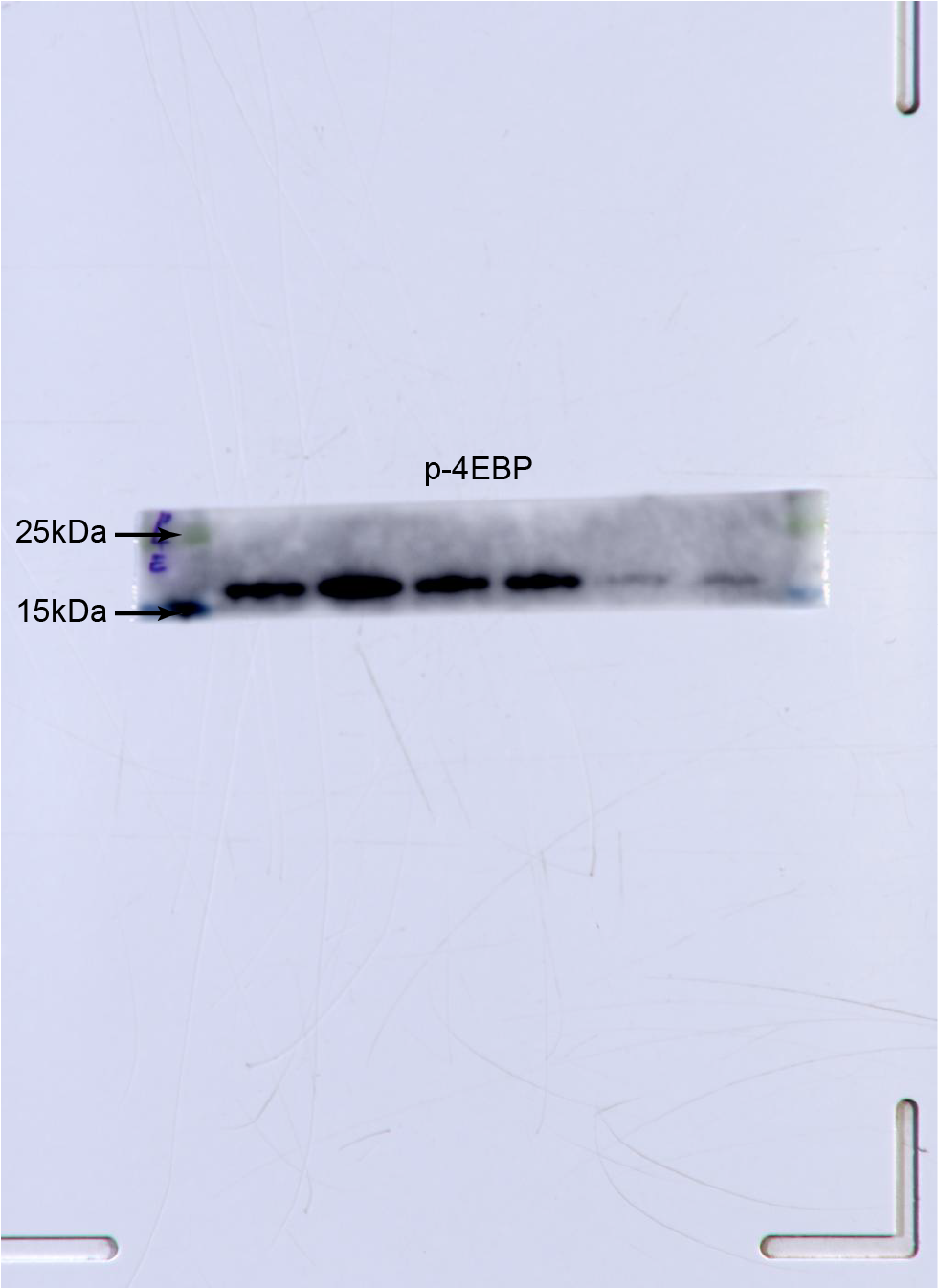

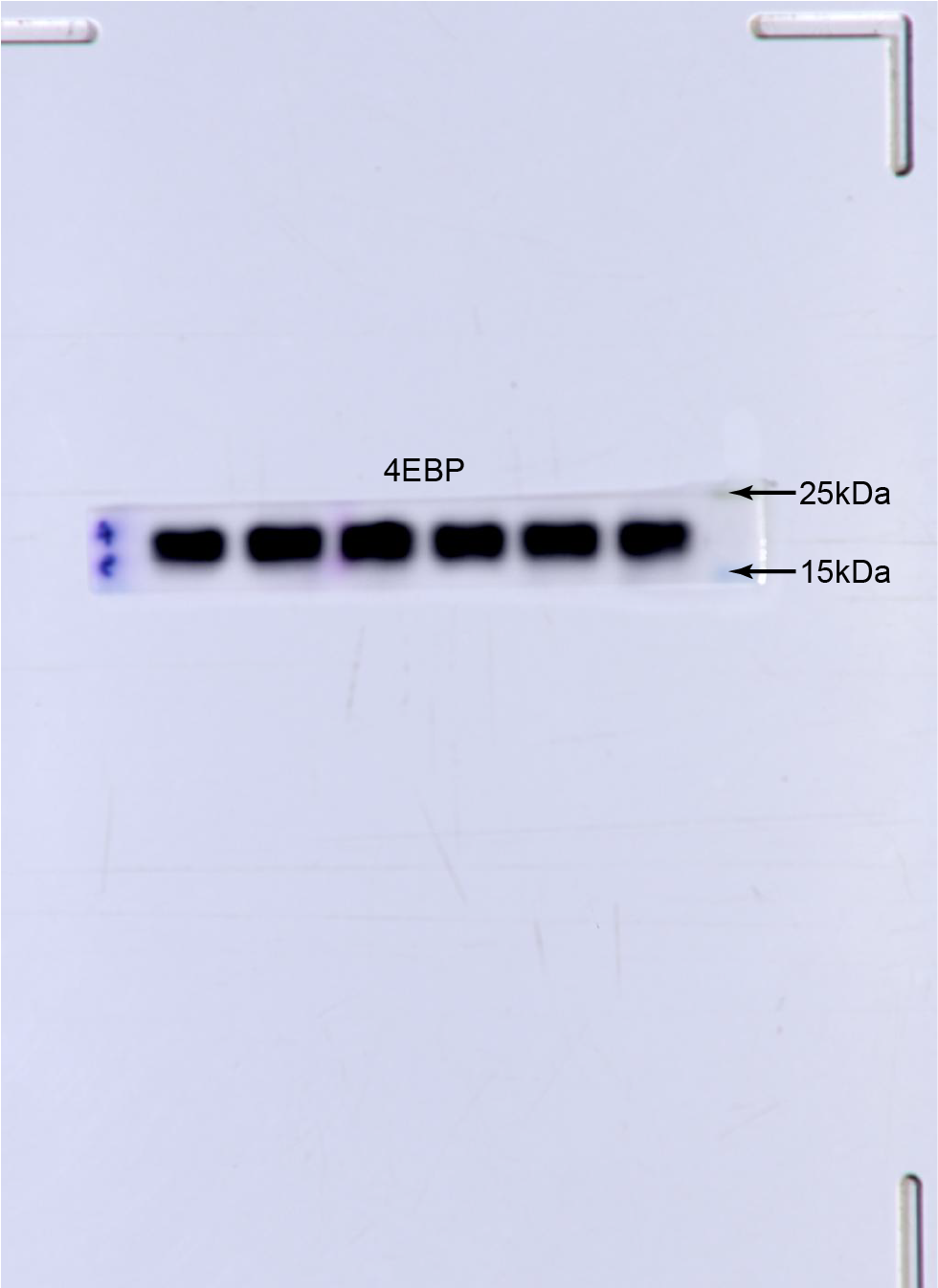

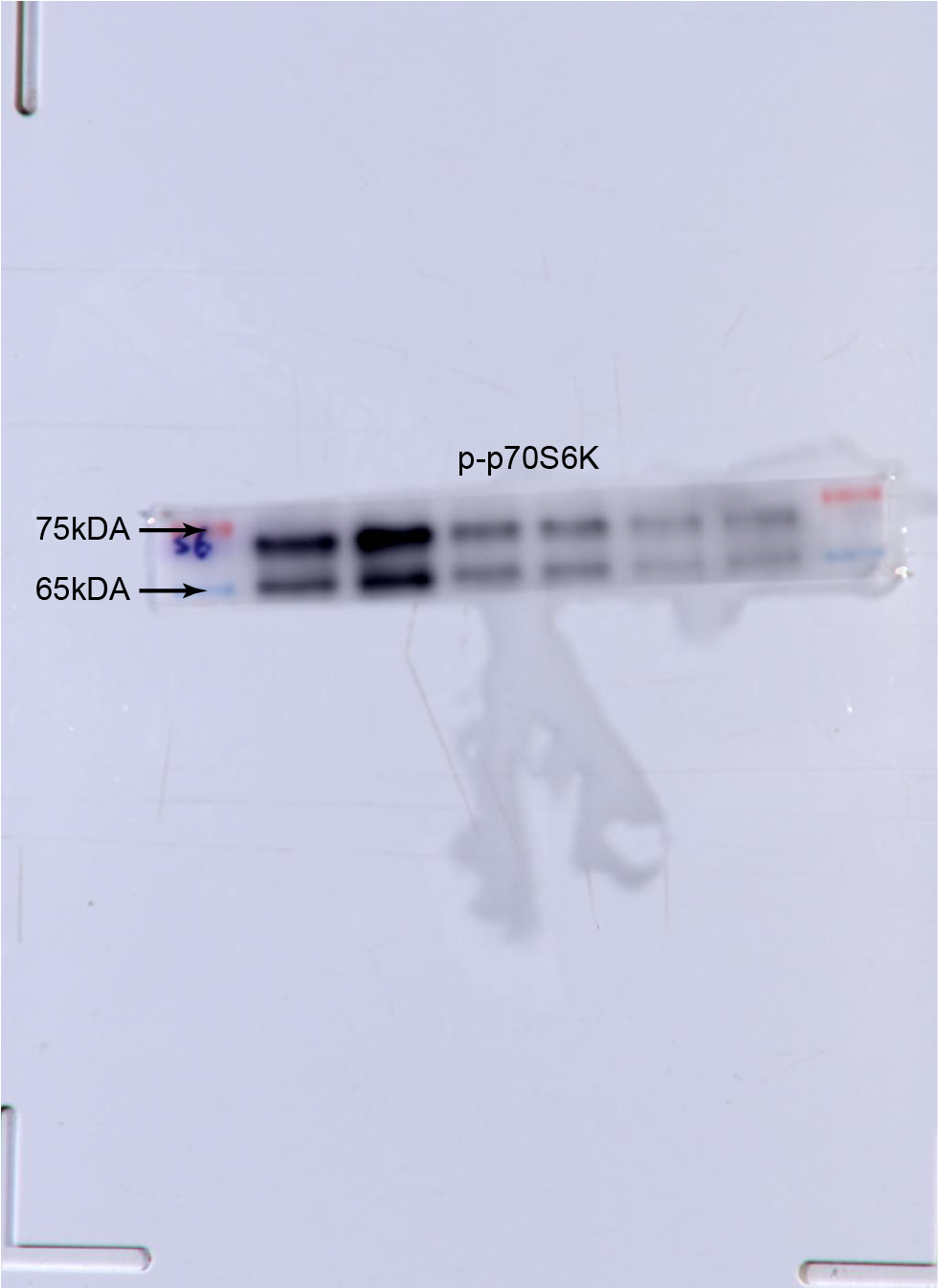

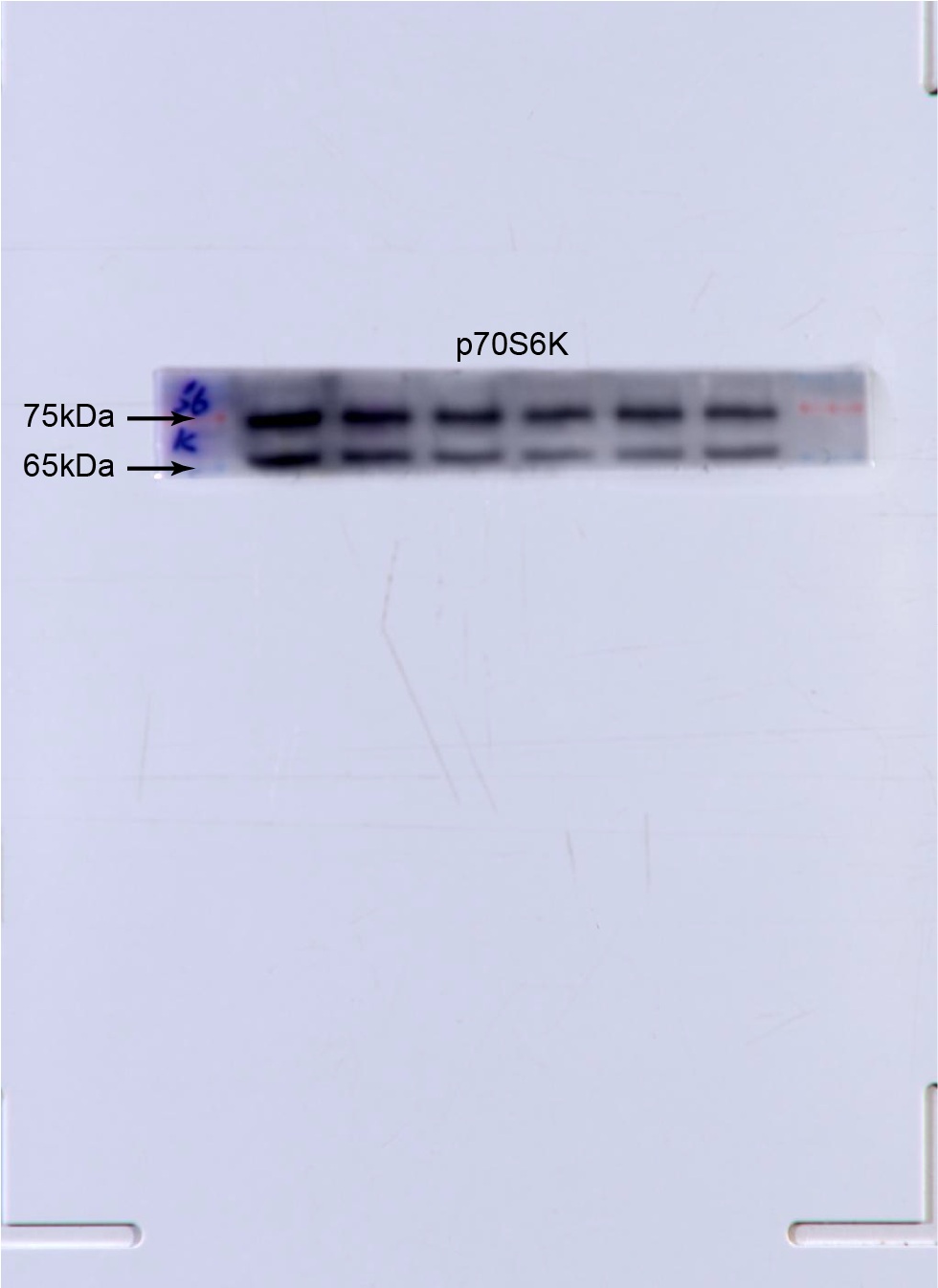

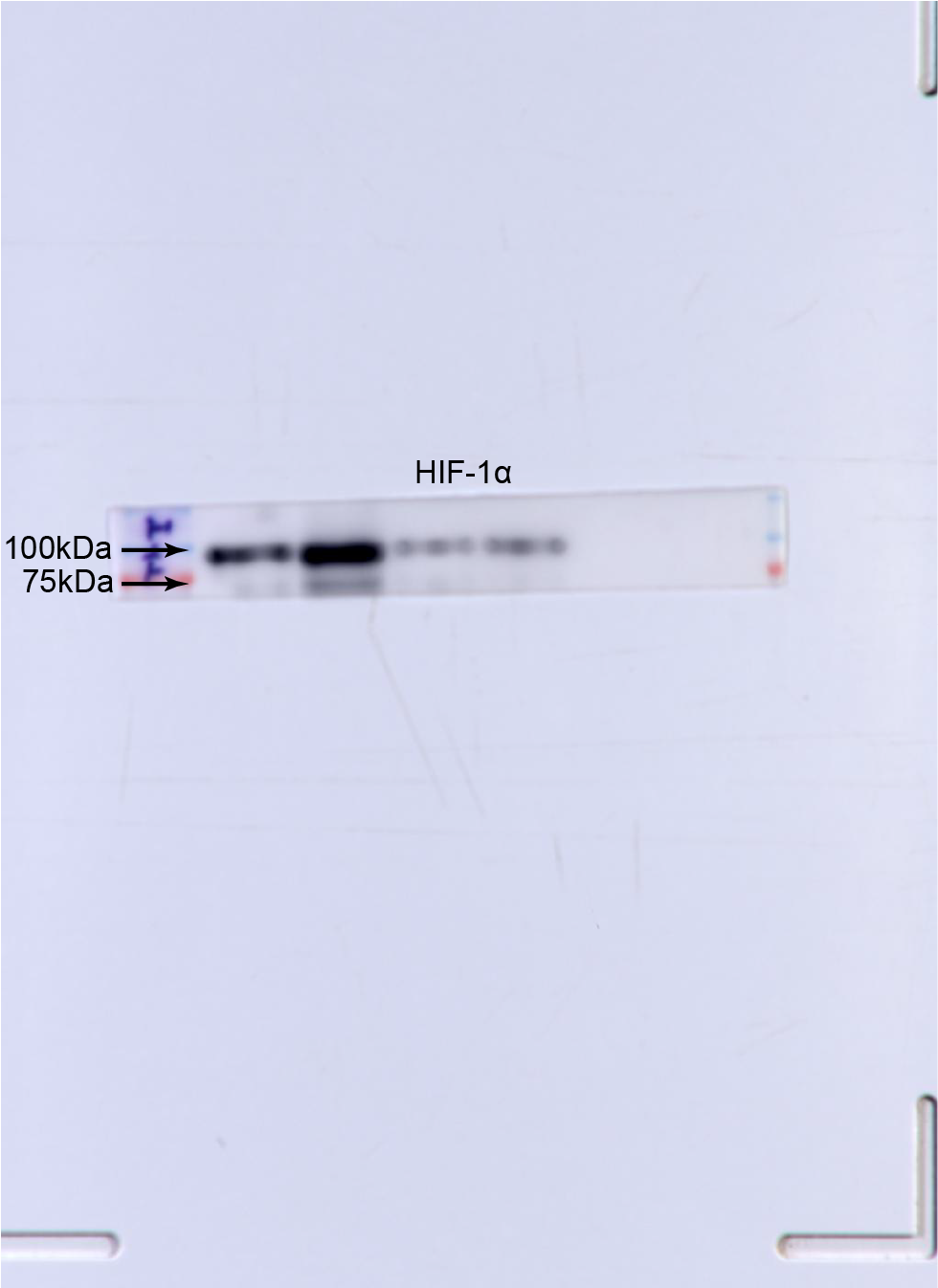

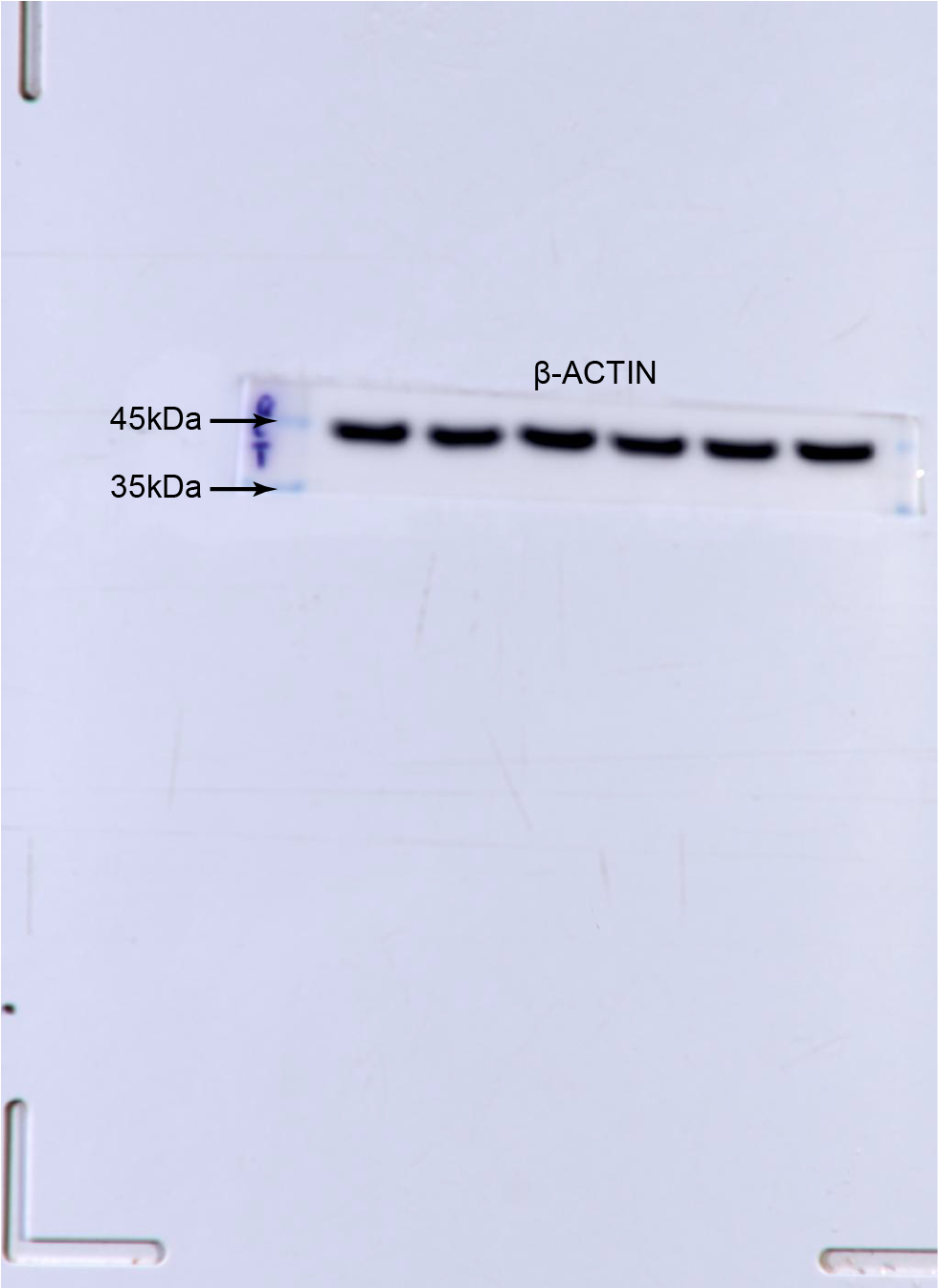


The images of western blotting in Fig 3C.


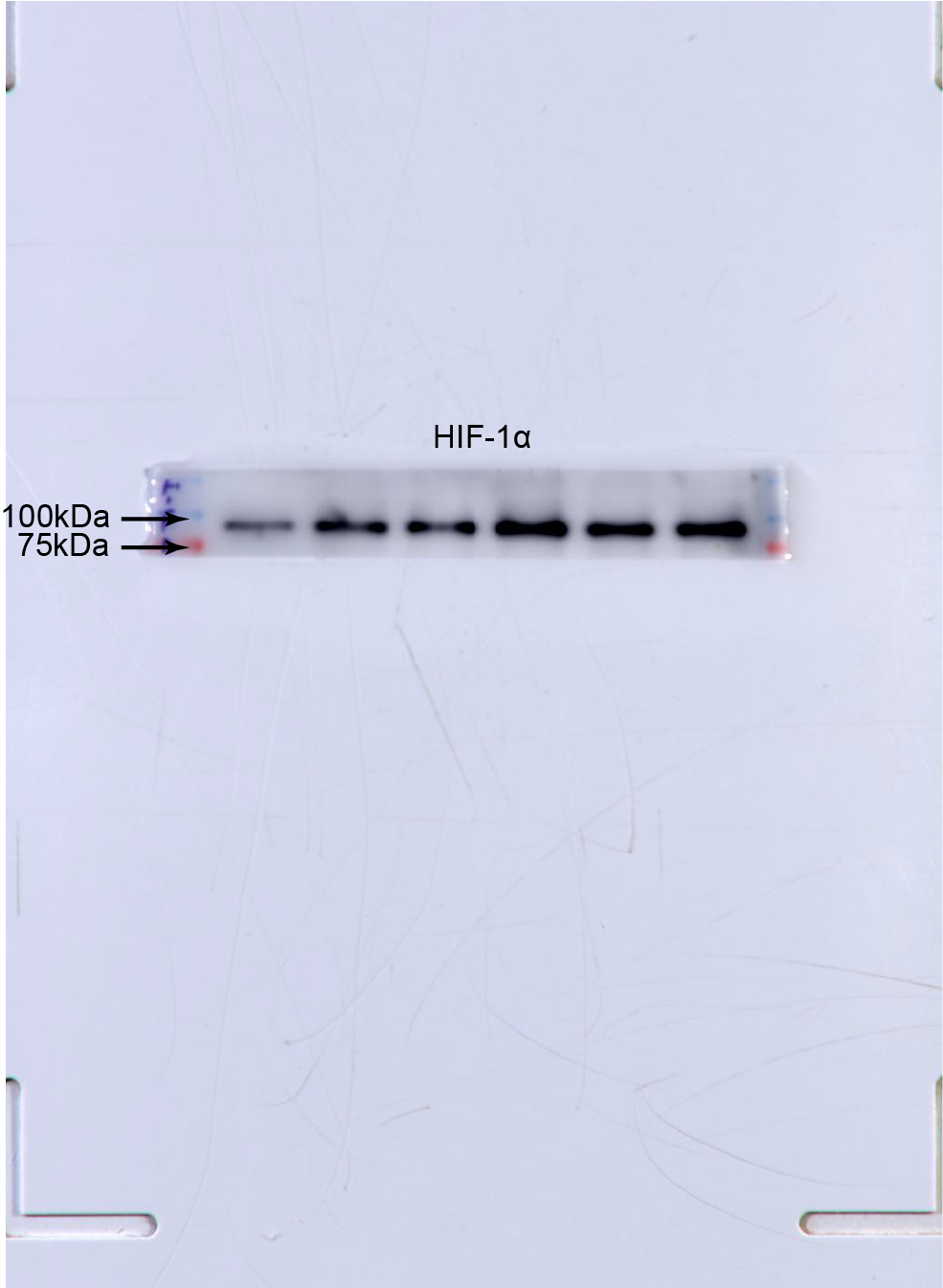

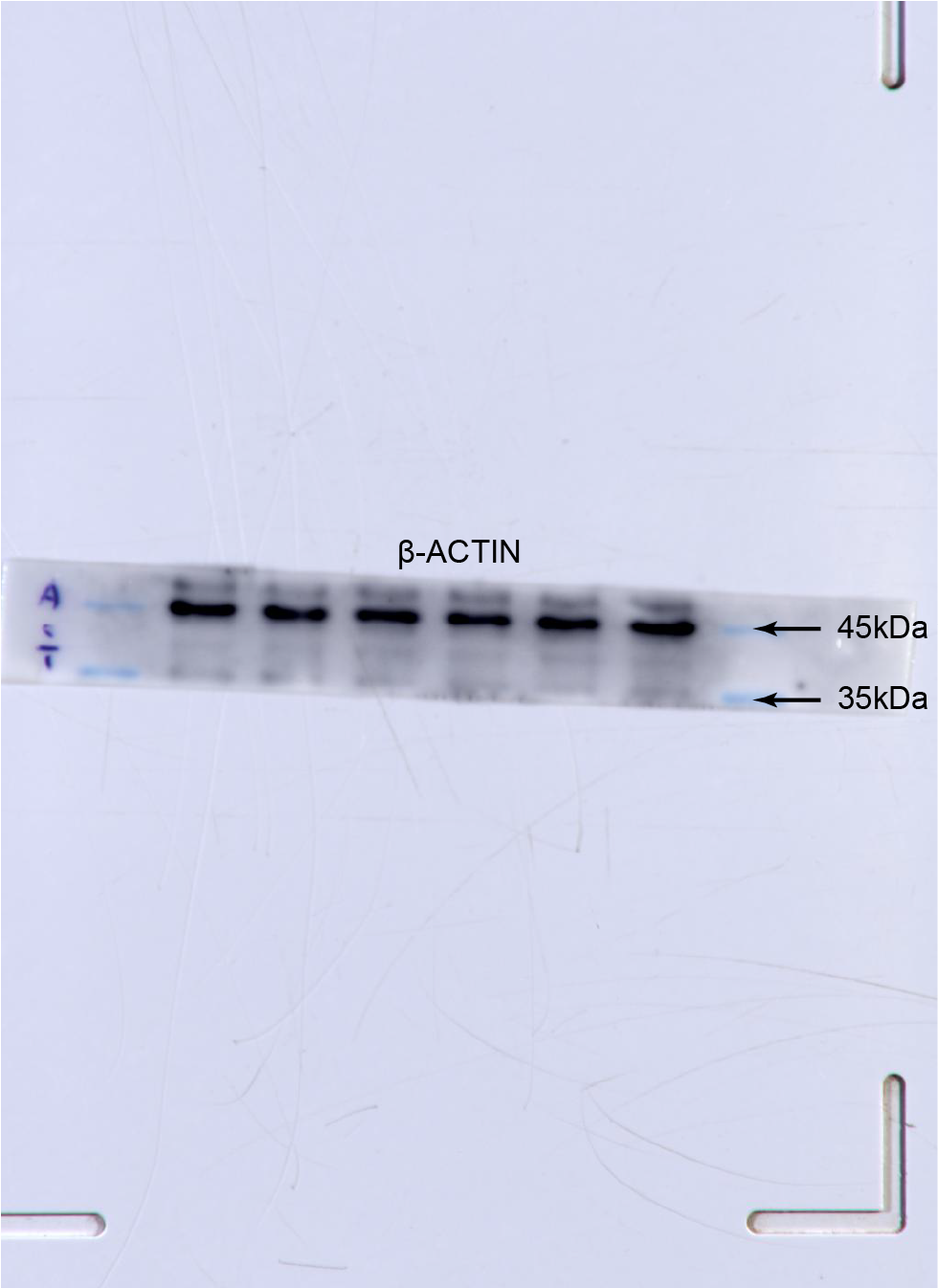


The images of western blotting in Fig 3E.


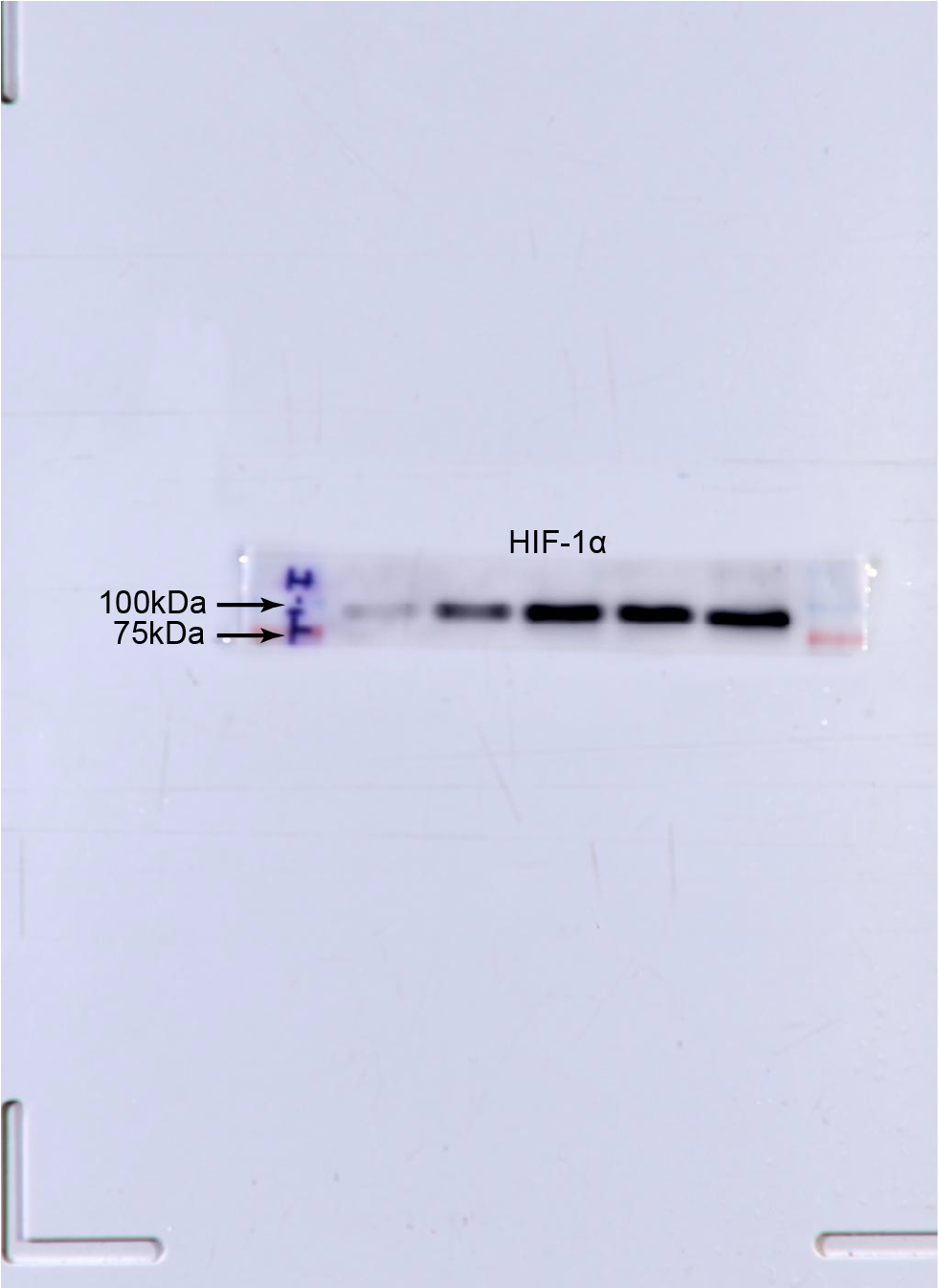

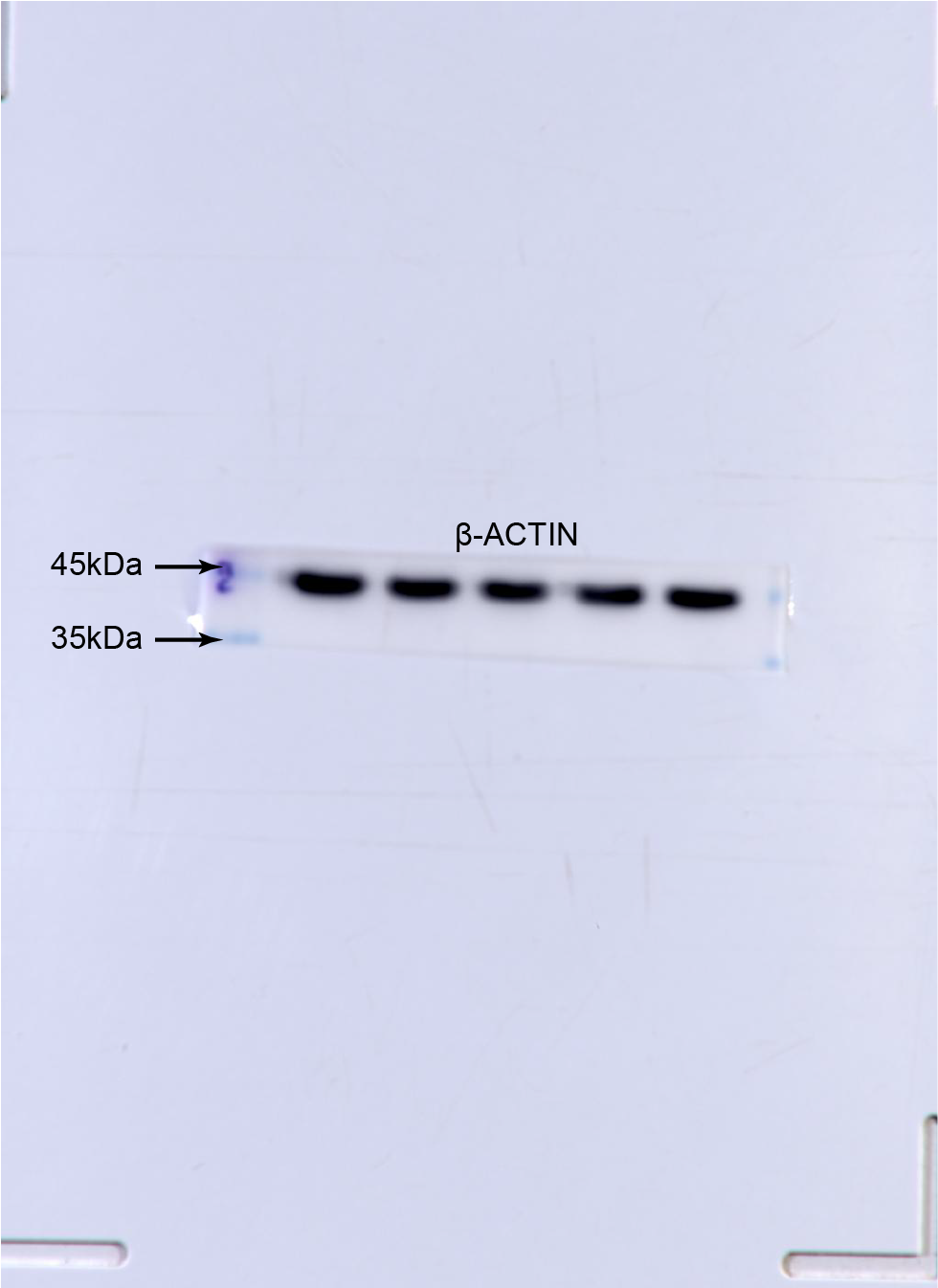


The images of western blotting in Fig 5I.


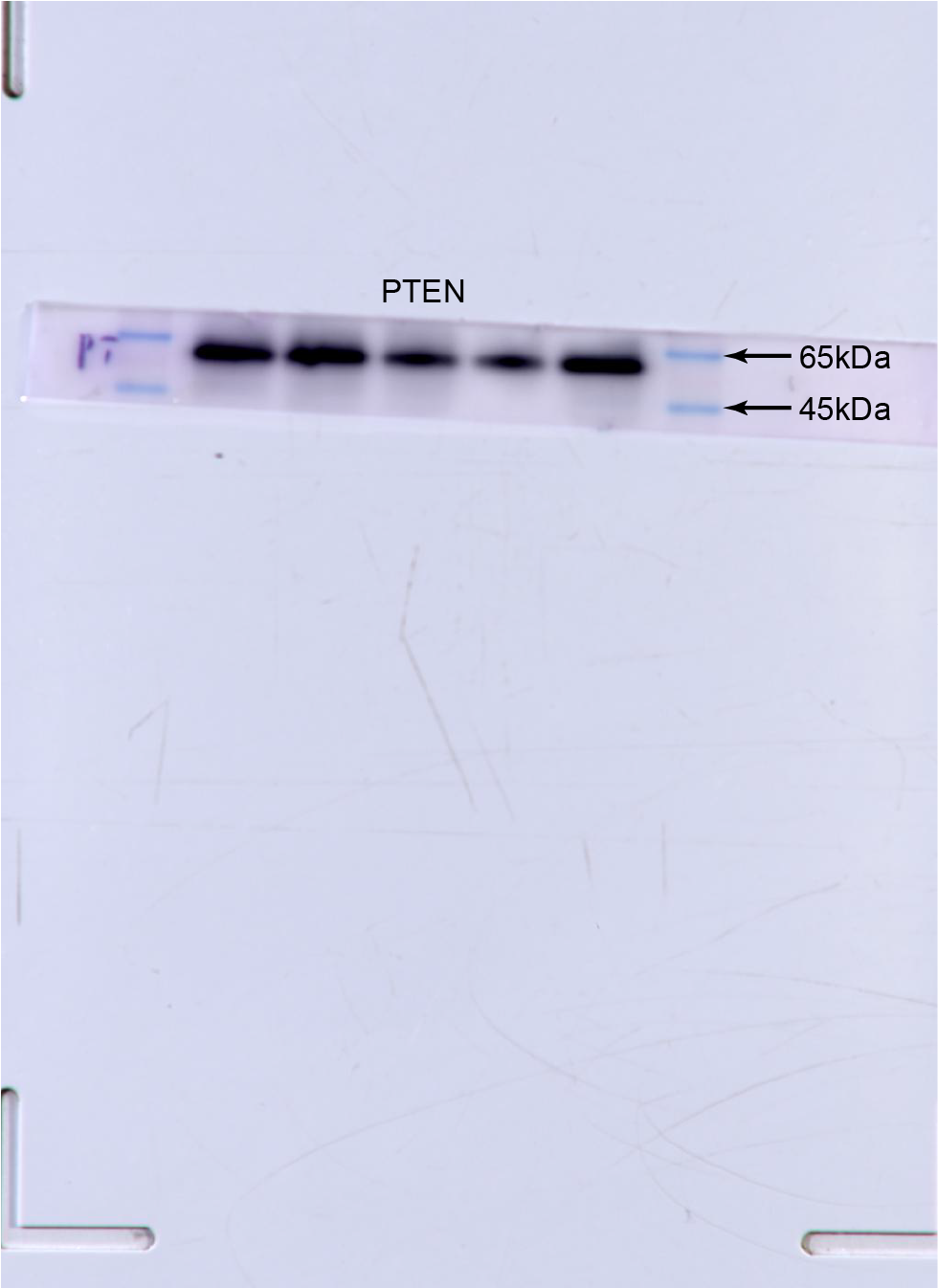

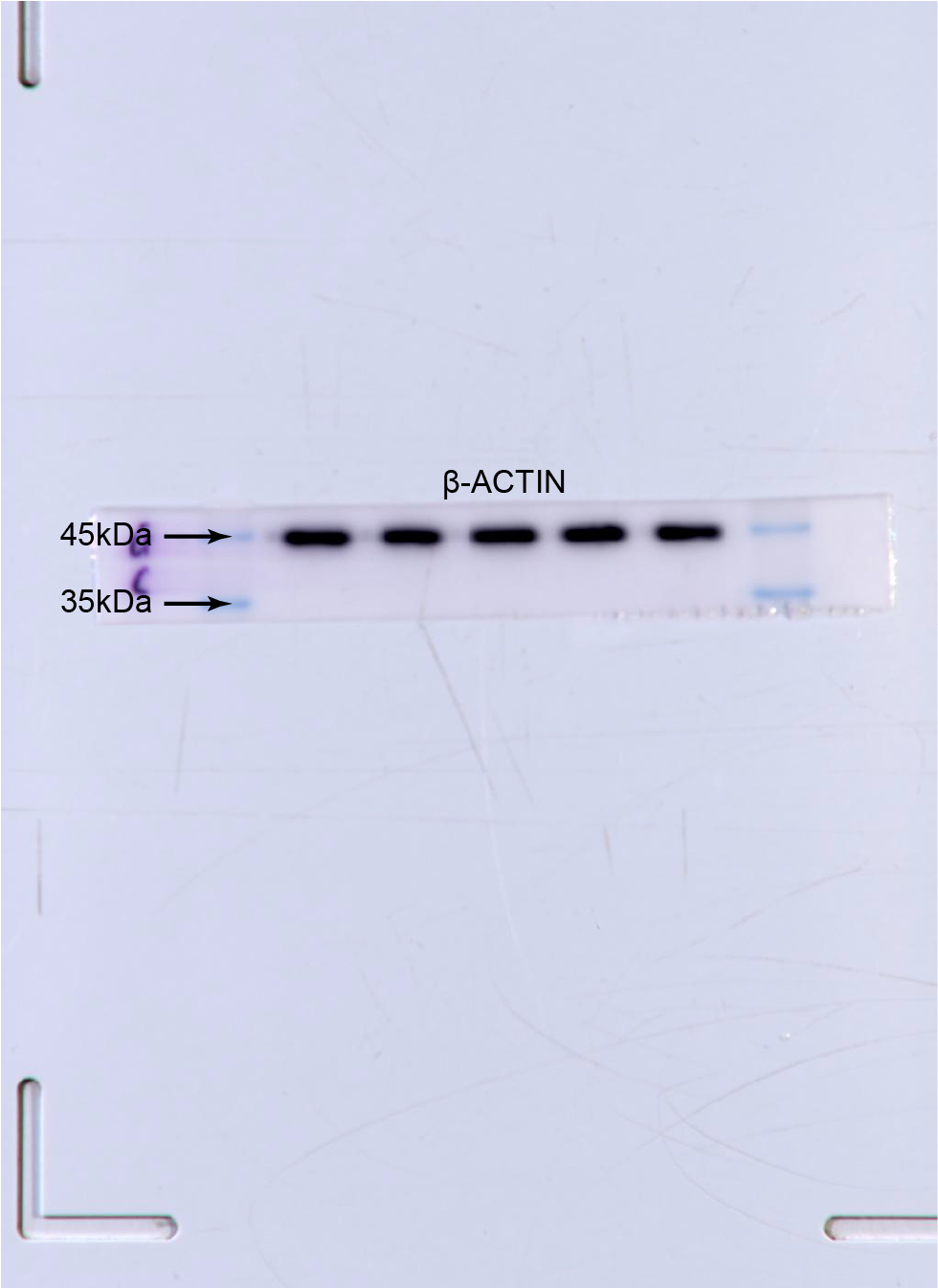


The images of western blotting in Additional File 3B.


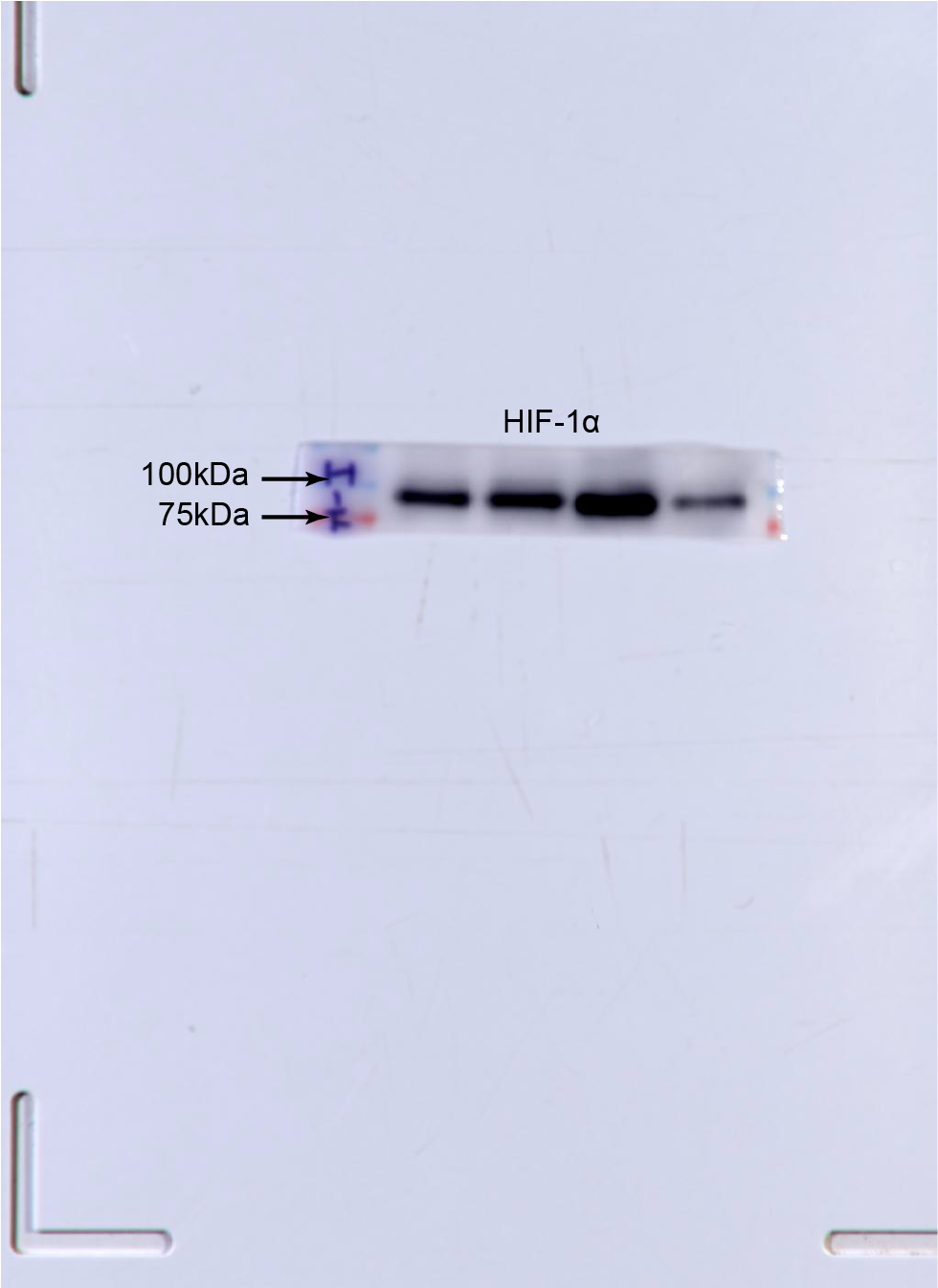

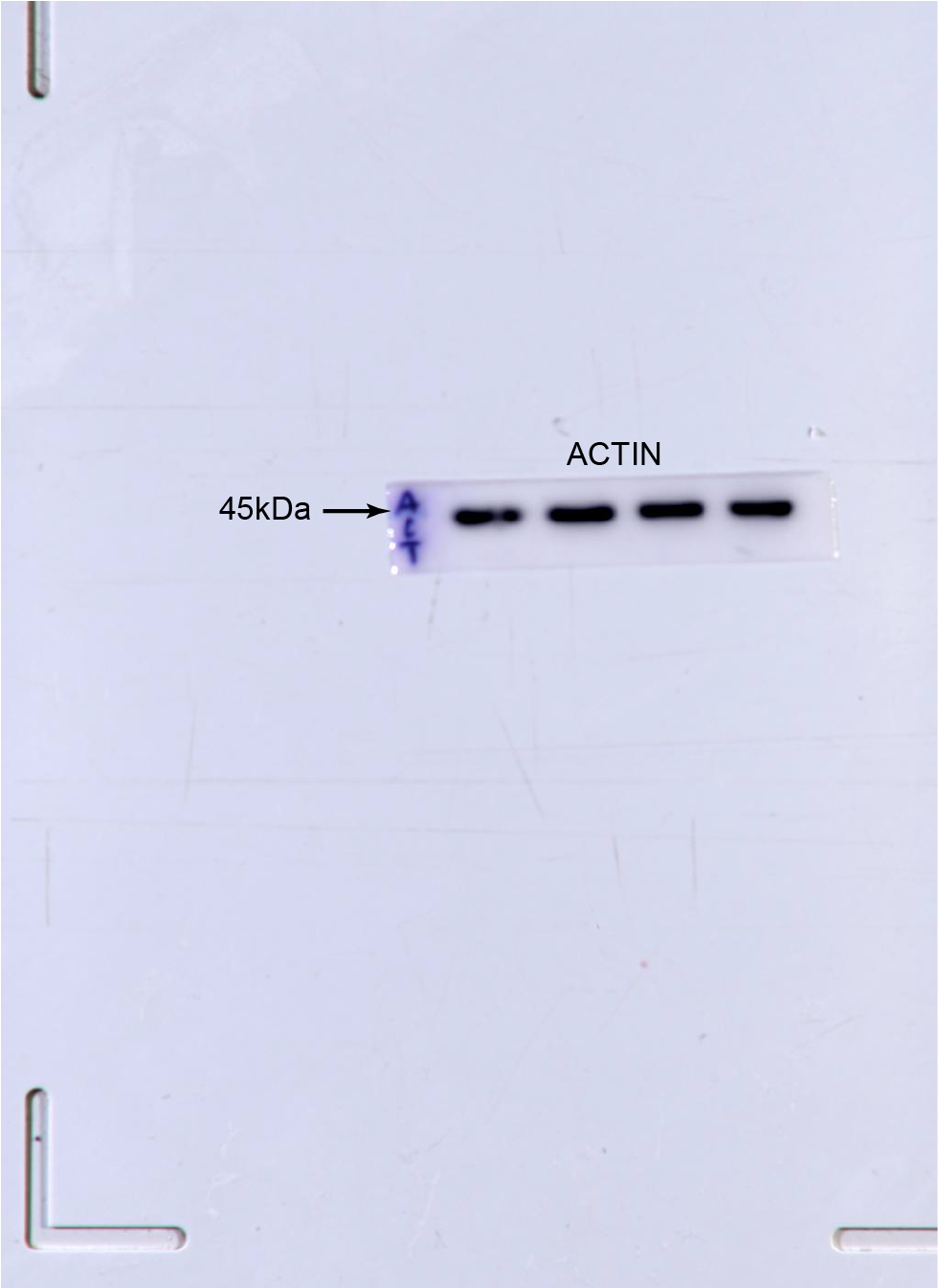

Supplement: Supplementary file 7 — Additional file 7: The raw images of western blotting. [file 12951_2022_1632_MOESM7_ESM.docx]
